# PARP1-catalyzed PARylation of YY1 mediates endoplasmic reticulum stress in granulosa cells to determine primordial follicle activation

**DOI:** 10.1038/s41419-023-05984-w

**Published:** 2023-08-15

**Authors:** Wei Chen, Qiukai E, Bo Sun, Pengxue Zhang, Nan Li, Shujia Fei, Yingnan Wang, Shuting Liu, Xiaoqiu Liu, Xuesen Zhang

**Affiliations:** 1 State Key Laboratory of Reproductive Medicine, Nanjing Medical University, Nanjing, 211166 China; 2 Department of Obstetrics and Gynecology, the Affiliated Jiangning Hospital of Nanjing Medical University, 211166 Nanjing, China; 3 Department of Pathogen Biology, College of Basic Medical Sciences, China Medical University, Shenyang, 110122 China; 4 Key Laboratory of Human Functional Genomics of Jiangsu Province, Nanjing Medical University, Nanjing, 211166 China; 5 College of Basic Medical Science, China Medical University, Shenyang, 110122 China

**Keywords:** Oogenesis, Mechanisms of disease

## Abstract

Although only a small number of primordial follicles are known to be selectively activated during female reproductive cycles, the mechanisms that trigger this recruitment remain largely uncharacterized. Misregulated activation of primordial follicles may lead to the exhaustion of the non-renewable pool of primordial follicles, resulting in premature ovarian insufficiency. Here, we found that poly(ADP-ribose) polymerase 1 (PARP1) enzymatic activity in the surrounding granulosa cells (GCs) in follicles determines the subpopulation of the dormant primordial follicles to be awakened. Conversely, specifically inhibiting PARP1 in oocytes in an in vitro mouse follicle reconstitution model does not affect primordial follicle activation. Further analysis revealed that PARP1-catalyzed transcription factor YY1 PARylation at Y185 residue facilitates YY1 occupancy at *Grp78* promoter, a key molecular chaperone of endoplasmic reticulum stress (ERS), and promotes *Grp78* transcription in GCs, which is required for GCs maintaining proper ERS during primordial follicle activation. Inhibiting PARP1 prevents the loss of primordial follicle pool by attenuating the excessive ERS in GCs under fetal bisphenol A exposure. Together, we demonstrate that PARP1 in GCs acts as a pivotal modulator to determine the fate of the primordial follicles and may represent a novel therapeutic target for the retention of primordial follicle pool in females.

## Introduction

For mammalian females, the pool of primordial follicles provides developing follicles and fertilizable ova resources throughout the reproductive lifespan [1, 2]. Primordial follicles are established perinatally and remain quiescent until activated. In each cycle, only a small number of primordial follicles are selectively activated through a process termed primordial follicle activation [2]. Primordial follicles are consisting of a central primary oocyte enveloped by a single layer of flattened pregranulosa cells (preGCs) [3]. Once activated, primordial follicles undergo rapid growth, leading to the enlargement of oocytes and proliferation of GCs [4, 5]. In particular, GCs show the most obvious morphological changes, from monolayer flattened to cuboidal in shape [6]. Accordingly, GCs are accompanied by a transition from a relatively transcriptionally inactive state to an active state [7]. Extensive studies have focused on the molecular interaction between oocytes and the surrounding GCs, and this bidirectional signaling is required to properly control primordial follicle activation [8, 9]. However, the mechanisms that trigger the selective activation of particular primordial follicles remain largely uncharacterized [10]. Misregulated activation of primordial follicles may lead to the exhaustion of the non-renewable pool of primordial follicles, resulting in premature ovarian insufficiency (POI) or early menopause [7, 11, 12]. Therefore, better understanding of these molecular systems governing the timely recruitment of primordial follicles into the growing population will help provide novel therapeutic targets for the retention of primordial follicle pool in females.

During the primordial follicle activation, both expanding oocytes and proliferating GCs undergo rapid growth, a process that may induce protein unfolding/misfolding [13, 14]. These unfolded/misfolded proteins accumulate in the endoplasmic reticulum (ER) lumen in the cells and trigger a cytoprotective response known as ER stress (ERS). In order to attenuate ERS and restore ER homeostasis, cells will activate the unfolded protein response (UPR) [15, 16]. The ERS triggers UPR by activating three main UPR sensors, including inositol-requiring enzyme 1α (IRE1α), PKR-like ER kinase (PERK) and activating transcription factor 6 (ATF6), on the ER membrane [17]. Under cellular homeostasis, these sensors are occupied by molecular chaperone Glucose-Regulated Protein 78 (GRP78) to maintain an inactive state. However, under ERS condition, GRP78 instead dissociates from these sensors, and activates UPR sensors to enhance protein folding and attenuate protein translation [18, 19]. Consistent with the function of UPR reported in other cell types, the UPR also plays a critical role during both oocyte and follicle development, to meet the increasing demand of protein synthesis, folding, modification, and transport [20, 21]. Though ERS is closely related to ovarian physiological process and pathogenesis, little is known about ERS in primordial follicle activation.

It has been reported that Poly-ADP-ribose polymerase (PARP)-mediated poly(ADP-ribosyl)ation (PARylation) can regulate protein biosynthetic process [22]. PARP1 is the most abundant member of the PARP family that is considered to be the “prototypical” PARP enzyme, catalyzing PARylation by utilizing nicotinamide adenine dinucleotide (NAD^+^) as a co-substrate [23, 24]. PARP1-catalyzed PARylation has been reported to be involved in a variety of biological processes, such as DNA damage repair, chromatin organization, chemoresistance, transcriptional control, mRNA stability, and DNA methylation [25, 26]. Especially, accumulated studies have revealed that PARP1 can catalyze PARylation of multiple transcription factors, including C/EBPβ [27], STAT1α [28], STAT3 [29], HIF-1α [30], AP-2 [31], p-TEFb [32], Smad3/4 [33], and modulate their transcriptional regulatory activities in different cells. Although PARP1 is essential for oogenesis and folliculogensis [34], and a recent clinical POI study even identified that ovarian GCs proliferation was inhibited by repressing PARP1 expression [35], whether PARP1 is involved in the activation of primordial follicles in still unclear. In the current study, we show that PARP1-catalyzed YY1 PARylation at Y185 residue facilitates YY1 occupancy to the *Grp78* promoter and promotes *Grp78* transcription in GCs during primordial follicle activation. PARP1-mediated ERS in GCs determines the subpopulation of the dormant primordial follicles to be awakened. Excessive ERS in GCs under fetal environment exposure to bisphenol A (BPA) accelerates primordial follicle activation, and inhibiting PARP1 catalytic activity can protect the loss of primordial follicle pool from the excessive ERS in GCs due to fetal BPA exposure.

## Results

### PARP1 enzymatic activity is indispensable for promoting primordial follicle activation in neonatal mice

**Fig. 1:**
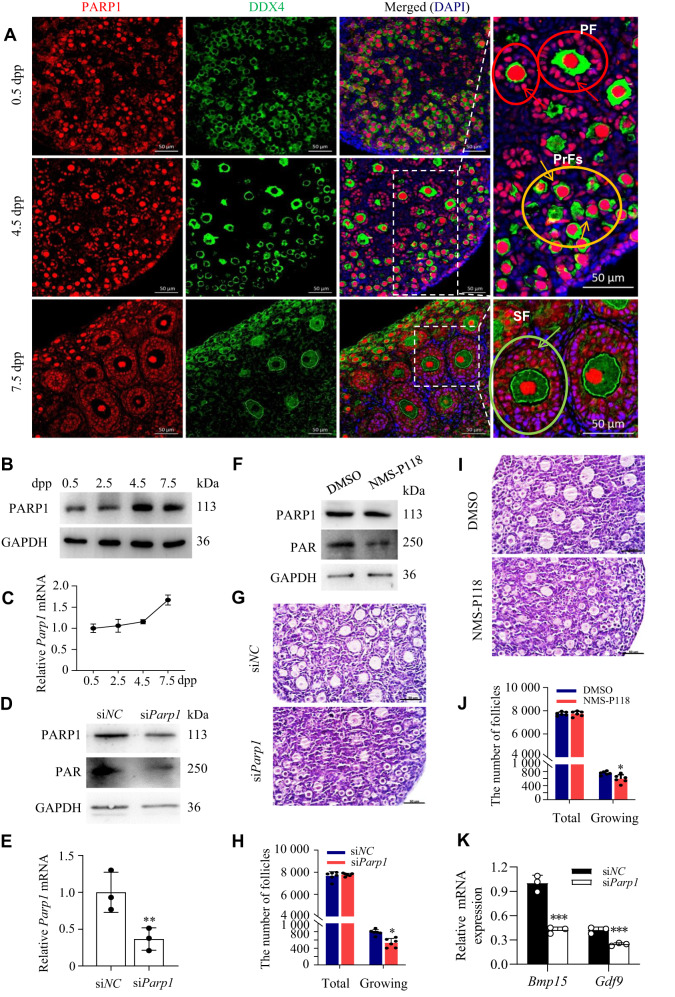
PARP1 enzymatic activity is indispensable for promoting primordial follicle activation in neonatal mice. **A** Representative immunofluorescence images of PARP1 (red) and oocyte specific marker DDX4 (green) in newborn ovaries. The nuclei were counter-stained with DAPI (blue). Scale bars: 50 μm. Yellow, red, and green circles represent primordial follicles (PrFs), primary follicles (PF), and secondary follicles (SF), separately. Yellow arrows pointing to the PARP1-positve preGCs in primordial follicles, red arrows pointing to the PARP1-positve GCs in primary follicles, and green arrows pointing to the PARP1-positve GCs in growing follicles. Scale bars: 50 μm in the zoomed-in images. Western blot analysis of PARP1 protein expression (**B**) and qRT-PCR analysis of *Parp1* mRNA level (**C**) in the neonatal mouse ovaries. **D** Relative PARP1 and PAR protein levels in 2.5 dpp mouse ovaries transfected with si*Parp1* for 3 days, and si*NC* used as a control. **E** Relative *Parp1* mRNA expression level in 2.5 dpp mouse ovaries transfected with si*Parp1* for 3 days, and si*NC* used as a control. Each bar represents the mean ± S.D. (*n* = 3). Asterisks indicate significant differences from si*NC* control (Student’s *t* test; **P < 0.01). **F** Western blot analysis of PARP1 and PAR expression in 2.5 dpp mouse ovaries treated with NMS-P118 for 3 days. GAPDH was used as a loading control. Representative images (**G**, **I**) and quantification of follicles (**H**, **J**) in the 2.5 dpp mouse ovaries transfected with si*Parp1* (**G**, **H**) or treated with NMS-P118 (**I**, **J**) for 3 days. Scale bar, 50 μm. Student’s *t* test: *P < 0.05. **K** qRT-PCR analysis of *Gdf9* and *Bmp15* mRNA levels in the 2.5 dpp mouse ovaries transfected with si*Parp1* and si*NC* groups. Student’s *t* test: **P < 0.01. ***P < 0.001.

Primordial follicles in mice form shortly after birth and some begin to develop into primary follicles by 5 dpp [36]. To determine the effects of PARP1 on primordial follicle activation, we examined its spatiotemporal expression in the neonatal mouse ovaries on 0.5, 4.5, and 7.5 dpp. Using immunofluorescence, we found that PARP1 was stained positively in the nuclei of both oocytes and preGCs in primordial follicles. Interestingly, with the development of follicles, PARP1 seemed to clearly accumulate in the nuclei of surrounding cuboidal GCs (white arrows) in the growing follicles on 4.5 dpp and 7.5 dpp ovaries, while the expression of PARP1 in oocytes did not show significant changes (Fig. 1A). Western blot and RT-PCR analyses also confirmed the increased expression of PARP1 in the 4.5 and 7.5 dpp ovaries compared to that in the 0.5 or 2.5 dpp (Fig. 1B, C). These results suggested that PARP-1 upregulation in GCs is closely associated with the activation of primordial follicles.

To further confirm this hypothesis, we knocked down PARP1 with *Parp1* siRNA (*si-Parp1*) in the 2.5 dpp mouse ovaries and cultured them for 3 days. Western blot and RT-PCR results demonstrated an obvious decrease of PARP1 expression upon *Parp1* knockdown (Fig. 1D, E). Furthermore, either depletion of PARP1 or inhibiting PARP1 enzymatic activity with NMS-P118 in the 2.5 dpp mouse ovaries followed by 3 days of culture led to an obvious decrease of PARP1 catalytic activity (Fig. 1D, F), together with a significant decrease of the number of growing follicles, compared to the relative controls, whereas the total number of oocytes was comparable across all groups (Fig. 1G–J). However, when we tested the expression of the genes essential for oocytes, including *Gdf9* and *Bmp15*, in si*Parp1*-treated neonatal mouse ovaries, we found that depletion of PARP1 significantly decreased the mRNA levels of these markers (Fig. 1K). These data indicated that inhibiting PARP1 enzymatic activity may sabotage the development potential of oocytes in the growing follicles.

### PARP1 enzymatic activity in GCs determines the activation of primordial follicles

**Fig. 2:**
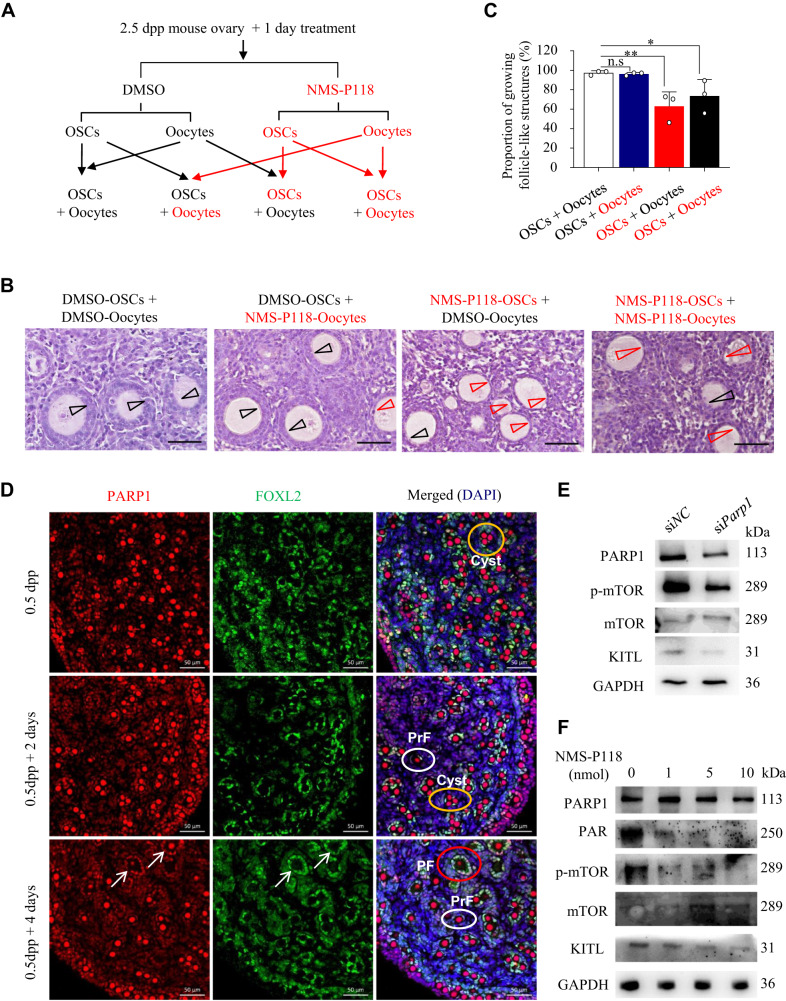
PARP1 enzymatic activity in GCs determines the activation of primordial follicles. **A** The experimental arrangement for the in vitro follicle reconstitution system. Oocytes and ovarian somatic cells (OSCs) derived from 2.5 dpp mouse ovaries pretreated with NMS-P118 or DMOS (control) for one day were separated, respectively, and then reconstituted as indicated. Representative images (**B**) and proportion of growing follicle-like structures (**C**) in the in vitro mouse follicle reconstitution system as indicated in **A**. Black arrow heads: activated follicular structure surrounded by multiple layers of granulosa cells; red arrow heads: Unactivated follicular structure surrounded by a single layer of flat granulosa cells. Scale bars: 50 µm. One-way ANOVA: n.s represents no statistical significance, *P < 0.05, **P < 0.01. **D** Representative immunofluorescence images of 0.5 dpp mouse ovaries after 2 or 4 days of culture, staining for PARP1 (red) and FOXL2 (green). The nuclei were counter-stained by DAPI (blue). Scale bars: 50 μm. Yellow, white, and red circles represent cyst, primordial follicles (PrF), and primary follicles (PF). Arrows pointing to the GCs in the PFs with positive co-localization signals of PARP1 and FOXL2. **E**, **F** Western blot analysis of PARP1, PAR, p-mTOR, mTOR and KITL expression in 2.5 dpp mouse ovaries transfected with si*Parp1* (**E**), or treated with NMS-P118 (**F**) for 3 days. GAPDH was used as a loading control. GAPDH was used as a loading control.

As PARP1 was also constitutively expressed in oocytes in the neonatal mouse ovaries, an in vitro follicle reconstitution system was thus applied to determine whether PARP1 may play a role in oocytes to regulate early follicle activation (Fig. 2A). Results showed that inhibiting PARP1 in oocytes with NMS-P118 did not affect the formation of growing follicle-like structures, whereas inhibiting PARP1 in preGCs did arrest the preGCs transformation from the flattened to cuboidal in shape and significantly inhibited the activation of primordial follicles (Fig. 2B, C). These results suggested that it is in GCs rather than in oocytes that PARP1 regulates primordial follicle activation. To further prove this hypothesis, we first co-stained PARP1 and FOXL2 in the cultured neonatal mouse ovaries, as FOXL2 is specifically expressed in GCs in ovaries [37, 38]. Results showed that, with the activation of primordial follicles, PARP1-positive GCs co-localized with FOXL2 (arrows in Fig. 2D). Given that the activation of mammalian target of rapamycin (mTOR) signaling and KIT ligand (KITL) in preGCs are necessary for primordial follicle activation [2, 7, 39, 40]. we next tested the effect of depleting or inhibiting PARP1 on the activation of mTOR and the expression of KITL in the neonatal mouse ovaries. As shown in Fig. 2E, F, either si*Parp1* or NMS-P118 treatment (resulting in a significant reduction of PAR) on 2.5 dpp mouse ovaries obviously inhibited the mTOR phosphorylation and the KITL expression in GCs. Taken together, PARP1 may promote the primordial follicle activation mainly through stimulating GC proliferation, in a mTOR/KITL-dependent manner.

### Downregulation of PARP1 or inhibiting its enzymatic activity prevents primordial follicle activation through inhibiting ERS signaling pathway in GCs

**Fig. 3:**
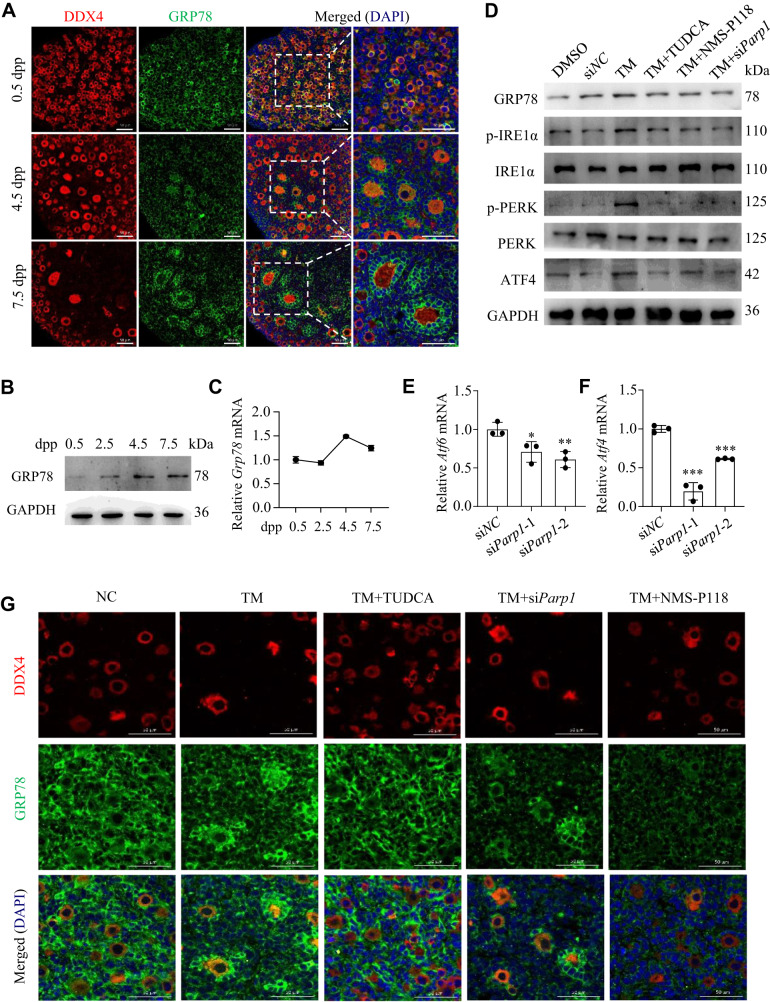
Downregulation of PARP1 or inhibiting its enzymatic activity prevents primordial follicle activation through inhibiting ERS signaling pathway. **A** Representative immunofluorescence images of GRP78 (green) and DDX4 (red) staining in newborn ovaries. The nuclei were counter-stained with DAPI (blue). Scale bars: 50 µm. The protein and mRNA levels of GRP78 analyzed by western blot (**B**) and qRT-PCR (**C**). **D** Western blot analysis of GRP78, p-IRE1α, IRE1α, p-PERK, PERK and ATF4 expression in 2.5 dpp mouse ovaries treated under indicated conditions for 3 days. GAPDH was used as a loading control. qRT-PCR analysis of *Atf6* (**E**) and *Atf4* (**F**) mRNA levels in 2.5 dpp mouse ovaries transfected with si*Parp1* for 3 days. One-way ANOVA: *P < 0.05, **P < 0.01, ***P < 0.001. **G** Representative immunofluorescence images of GRP78 (green) and DDX4 (red) in 2.5 dpp mouse ovaries treated under indicated conditions for 3 days. The nuclei were counter-stained with DAPI (blue). Scale bars: 50 µm.

Activation of ER stress in GCs, oocytes/embryos, and cumulus-oocyte complexes during folliculogenesis has been widely accepted [41]. As one of the master regulator in ERS, GRP78 is found in all eukaryotes on the membrane of ER under both physiological and pathological conditions [42]. To test the hypothesis that ERS is also playing a role in regulating primordial follicle activation, immunofluorescence and western blot assays were employed to detect the cellular localization and expression dynamics of GRP78 in neonatal mouse ovaries. Results showed that GRP78 was primarily expressed in the cytoplasm of oocytes in 0.5 dpp ovaries. Interestingly, its expression started to accumulate into the GCs in the growing follicles from 4.5 dpp to 7.5 dpp, with a similar expression pattern as that of PARP1 (Fig. 3A). Accordingly, the protein and mRNA levels of GRP78 also showed an obvious increase from 0.5 dpp to 7.5 dpp ovaries (Fig. 3B, C). The similar spatiotemporal expression profiling of these two proteins raised a possibility that PARP1 may regulate GRP78 expression, given that PARP1 has a well-established role in gene regulation [25, 43], and thus participate in ERS during primordial follicle activation.

Since low doses of tunicamycin (TM) treatment can induce moderate ERS in multiple cells or tissues [44], we treated 2.5 dpp mouse ovaries with 50 nM of TM and then examined the expression of ERS-related molecules. As shown in Fig. 3D, low doses of TM treatment for 3 days significantly triggered the expression of ERS-related molecules, including the upregulation of GRP78 and ATF4, as well as the phosphorylation of IRE1α and PERK. However, depleting PARP1 or inhibiting its enzymatic activity antagonized the TM-induced expression of these markers, an effect similar to that of TUDCA treatment, the classic ERS-inhibiting drug (Fig. 3D). Besides, PARP1 downregulation reduced the mRNA levels of *Atf6* and *Atf4* (Fig. 3E, F). Furthermore, depleting or inhibiting PARP1 could dampen the stimulatory effect of TM on primordial follicle overactivation, as well as the expression pattern of GRP78 (Fig. 3G). These results further confirmed our hypothesis that PARP1 may regulate GRP78 expression and mediate ERS in GCs in the neonatal mouse ovaries.

### PARP1 neither regulates GRP78 expression through direct PARylation of GRP78 nor functions as a transcription cofactor to regulate *Grp78* transcription

**Fig. 4:**
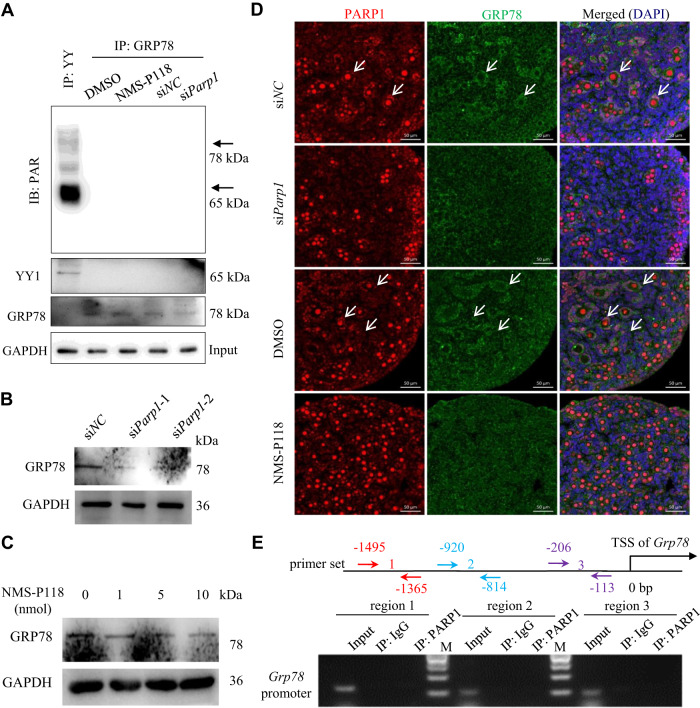
PARP1 neither regulates GRP78 expression through direct PARylation of GRP78 nor functions as a transcription cofactor to regulate *Grp78* transcription. **A** 2.5 dpp mouse ovaries were treated under indicated conditions for 3 days. The ovarian lysates were immunoprecipitated with the anti-YY (positive control), or anti-GRP78 antibody, respectively, and detected by anti-PAR. GAPDH as a loading control. Western blot analysis of GRP78 expression in 2.5 dpp mouse ovaries transfected with si*Parp1* (**B**) or treated with NMS-P118 (**C**) for 3 days. GAPDH was used as a loading control. **D** Representative immunofluorescence images of PARP1 (red) and GRP78 (green) staining in 2.5 dpp mouse ovaries treated under indicated conditions for 3 days. The nuclei were counter-stained with DAPI (blue). Scale bars: 50 µm. Arrows pointing to GCs in the growing follicles. **E** Schematic presentation for primer sets (3 different colors) to amplify the potential PARP1 binding region within the proximal promoter region of *Grp78* (top). Bottom: ChIP-PCR analysis of PARP1 binding at the proximal promoter region of *Grp78* in 4.5 dpp mouse ovaries. IgG was used as a negative control.

One recent study has identified that, during the ERS response, GRP78 can be ADP-ribosylated paralleling translational inhibition [45]. Given that PARP1 contains the ability to catalyze PARylation of various protein substrates, we wondered whether PARP1 may also catalyze the PARylation of GRP78 in neonatal mouse ovaries. However, we barely detected the PARylation of GRP78 (Note: PARylation of YY1 was used as a positive control) (Fig. 4A). To further test the effect of PARP1 on GRP78 expression in the neonatal mouse ovaries, we transiently transfected the neonatal mouse ovaries with si*Parp1* or added NMS-P118 to the cultured ovaries, followed by the detection of GRP78 expression. As shown in Fig. 4B, C, either si*Parp1* or NMS-P118 treatment significantly decreased GRP78 expression. Consistently, either treatment prevented the activation of primordial follicles (Fig. 4D). Since PARP1 can serve as a transcriptional cofactor to regulate its downstream target gene expression [46, 47], we then performed chromatin immunoprecipitation (ChIP) experiment in the 4.5 dpp mouse ovaries with the anti-PARP1 antibody and tested whether PARP1 could occupy the *Grp78* gene promoter. Using specific primer sets spanning different proximal promoter regions of *Grp78* that contain ER stress response element, we clearly demonstrated that PARP1 did not bind to the *Grp78* promoter (Fig. 4E). These results suggested that PARP1 may regulate GRP78 transcription via a mechanism independent of direct transcriptional regulation.

### PARP1-catalyzed PARylation of YY1 on *Grp78* promoter accelerates *Grp78* transcription

**Fig. 5:**
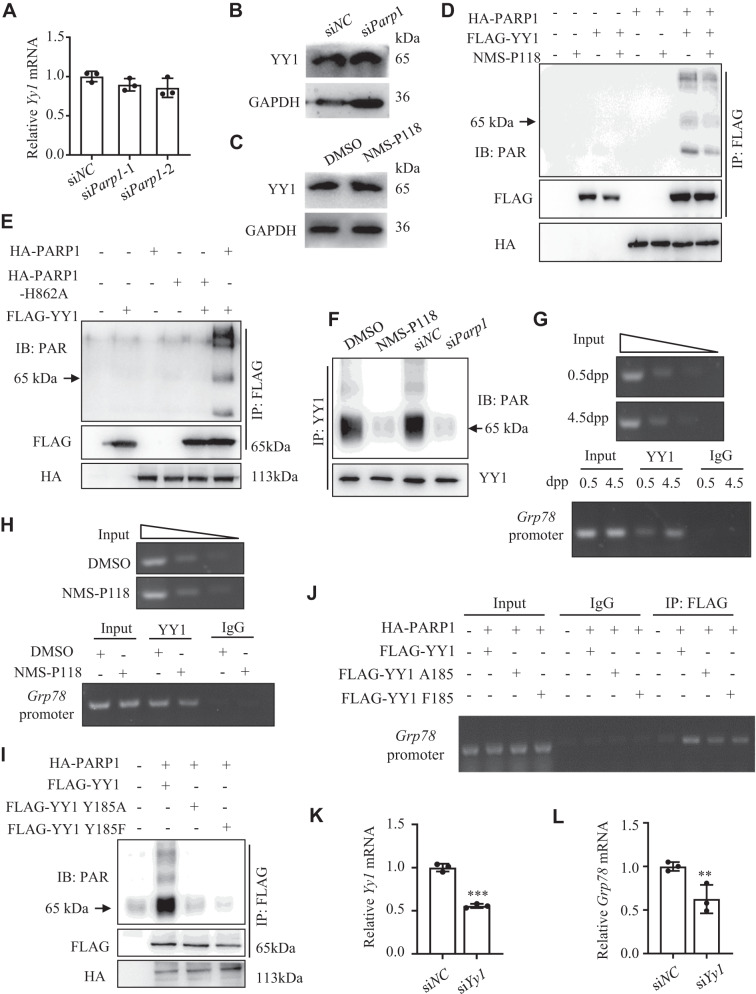
PARP1-catalyzed PARylation of YY1 on *Grp78* promoter regulates *Grp78* transcription. Relative *Yy1* mRNA (**A**) and protein (**B**) expression in 2.5 dpp mouse ovaries transfected with si*Parp1* for 3 days. **C** Western blot analysis of YY1 expression in 2.5 dpp mouse ovaries treated with NMS-P118 for 3 days in vitro. **D** Western blots showing the levels of YY1-PAR on immunoprecipitated YY1 (IP: FLAG) from HEK293T cells co-transfected with PARP1 and YY1, followed by NMS-P118 treatment for 36 h. **E** Western blots showing the levels of YY1-PAR on immunoprecipitated YY1 (IP: FLAG) from HEK293T cells co-transfected with YY1 and wild-type PARP1 or with catalytically inactive PARP1-H862A. **F** Western blots showing the levels of YY1-PAR on immunoprecipitated YY1 (IP: YY1) from 2.5 dpp mouse ovaries treated with NMS-P118 or transfected with si*Parp1* for 3 days. ChIP-PCR analysis of YY1 binding within the promoter region of *Grp78* in newborn mouse ovaries at 0.5 dpp and 4.5 dpp (**G**), or in 2.5 dpp mouse ovaries treated with NMS-P118 for 3 days (**H**). IgG was used a negative control. **I** Western blots showing the levels of YY1-PAR on immunoprecipitated YY1 (IP: FLAG) from HEK293T cells co-transfected with PARP1 and wild-type YY1, or with Y185 mutated YY1. **J** ChIP-PCR analysis of YY1 binding within the promoter region of *Grp78* in 2.5 dpp mouse ovaries co-transfected with PARP1 and wild-type YY1, or with Y185 mutated YY1. IgG was used as a negative control. qRT-PCR analysis of *Yy1* (**K**) and *Grp78* (**L**) mRNA expression levels in the 2.5 dpp mouse ovaries transfected with si*Yy1* and si*NC* for 3 days. Student’s *t* test: **P < 0.01, ***P < 0.001.

Accumulated studies have shown that PARP1-catalyzed PARylation of some transcription factors can affect their specific DNA binding abilities or modulate their activities to regulate gene transcription [48–50]. Given the facts that transcription factor Yin Yang 1 (YY1) can selectively regulate GRP78 transcription by occupying the *Grp78* promoter under ERS conditions [51], that PARylation of YY1 has been observed under DNA damage in tumor cells [52], and that YY1 has been implicated in an important role in oocyte maturation and GC expansion [53], we therefore asked whether PARP1 may target YY1 for PARylation, which then affects *Grp78* promoter occupation and *Grp78* transcription in GCs during primordial follicle activation. To test this hypothesis, we first showed that neither si*Parp1* treatment nor inhibiting PARP1 enzymatic activity altered the expression of YY1 (Fig. 5A–C). However, inhibiting PARP1 enzymatic activity obviously decreased PARylation of YY1 (Fig. 5D). Since PARP1 histidine 862 (H862) is the residue responsible for the polymerase activity of PARP1 [54], we then mutated H862 into alanine (H862A) and compared the catalytic activity of the mutant form with the wild type PARP1. Results showed that H862A mutant failed to catalyze the PARylation of YY1 (compared lane 5 to lane 6, Fig. 5E). To observe this effect in vivo, we then treated the 2.5 dpp mouse ovaries with NMS-P118 or si*Parp1* for 3 days. Results showed that either treatment nearly completely abolished YY1 PARylation compared to the respective controls (Fig. 5F). Next, we performed ChIP assay in the newborn mouse ovaries, and found that YY1 occupancy at the *Grp78* promoter region showed an obvious enrichment in 4.5 dpp ovaries than 0.5 dpp, coinciding with the timing of activation primordial follicles in the newborn mouse ovaries (Fig. 5G). Inhibiting PARP1 activity with NMS-P118 in 2.5 dpp mouse ovaries for 3 days obviously reduced YY1 occupancy at the *Grp78* promoter (Fig. 5H). Given that YY1 can be PARylated on Y185 residue by PARP1 [55], we then generated Y185A or Y185F mutant form of YY1 and transfected them with PARP1 into the HEK293 cells and cultured ovaries, respectively. Compared to the controls, these two mutants dramatically decreased the PARylation of YY1 in cells (Fig. 5I). And these two mutants also obviously decreased YY1 occupancy at the *Grp78* promoter in the ovaries (Fig. 5J). Finally, knockdown of YY1 in 2.5 dpp ovaries significantly reduced both *Yy1* and *Grp78* mRNA levels (Fig. 5K, L). Together, our findings indicated that PARP1-catalyzed PARylation of YY1 at *Grp78* promoter in GCs is required for *Grp78* transcription and may dominate the primordial follicle activation in newborn mice, mainly through inducing ERS in GCs.

### Melatonin protects the fetal mouse ovaries from gestational BPA exposure and reduces hyperactivation of primordial follicles in the offspring mice

**Fig. 6:**
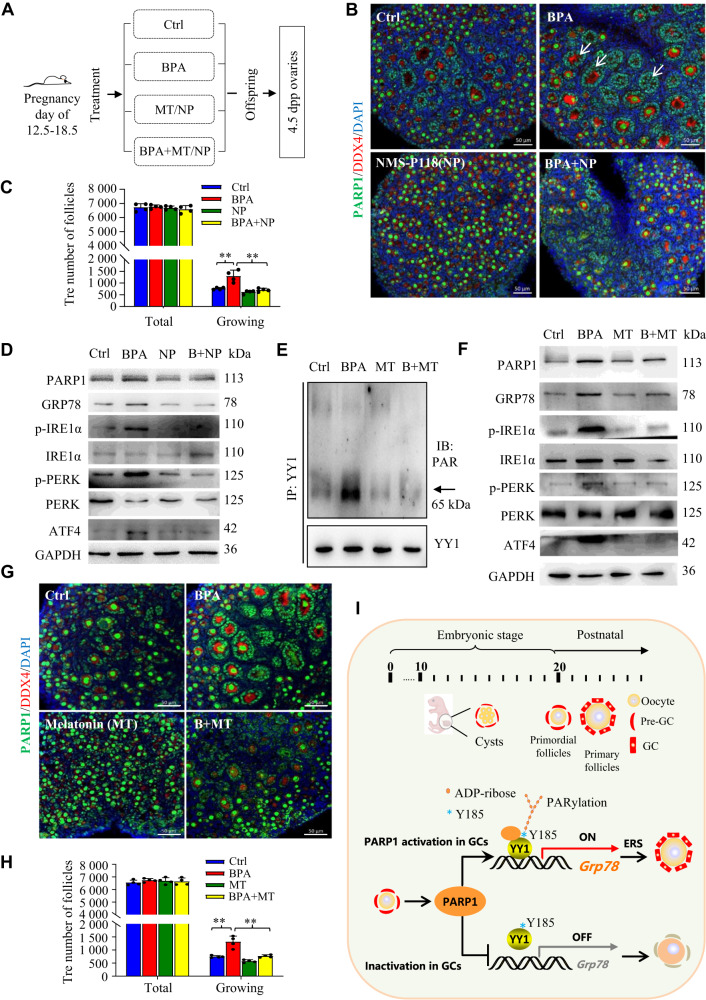
Melatonin protects the fetal mouse ovaries from gestational BPA exposure and reduces hyperactivation of primordial follicles in the offspring mice. **A** Schematic diagram showing the experimental arrangement. MT, Melatonin; NP, NMS-P118. Representative immunofluorescence images of PARP1 (green) and DDX4 (red) (**B**), and quantification of follicles (**C**) in the 4.5 dpp mouse ovaries of newborn offspring under fetal exposure to PBS (Control), BPA, NMS-P118 (NP), or BPA + NP. The nuclei were counterstained with DAPI (blue). Scale bars: 50 μm. Data in **C** was expressed as mean ± S.D. (*n* = 4). One-way ANOVA: **P < 0.01. **D** Western blots showing the levels of PARP1, GRP78, p-IRE1α, IRE1α, p-PERK, PERK and ATF4 in the 4.5 dpp mouse ovaries of offspring as detailed in **B**. **E** Western blots showing the levels of YY1-PAR on immunoprecipitated YY1 (IP: YY1) from the 4.5 dpp mouse ovaries of offspring under fetal exposure to PBS (Control), BPA, Melatonin (MT), or BPA + MT. **F** Western blots showing the levels of PARP1, GRP78, p-IRE1α, IRE1α, p-PERK, PERK and ATF4 in the 4.5 dpp mouse ovaries of offspring as described in **E**. Representative immunofluorescence images of PARP1 (green) and DDX4 (red) (**G**), and quantification of follicles (**H**) in the 4.5 dpp mouse ovaries of offspring as described in **E**. The nuclei were counter-stained with DAPI (blue). Scale bars: 50 μm. Data in **H** was expressed as mean ± S.D. (*n* = 4). One-way ANOVA: **P < 0.01. **I** Working model showing the proposed role of PARP1 in the selective activation of primordial follicle. See details in Discussion.

Bisphenol A (BPA) is an endocrine-disrupting chemical with ubiquitous human exposure on reproduction [56]. Previous studies have shown that either neonatal exposure to BPA or maternal exposure during pregnancy reduces the pool of primordial follicles in the offspring ovary by stimulating the neonatal initial primordial follicle recruitment [57, 58]. Given that low-dose of BPA has been reported to induce excessive ERS in testicular Sertoli cells [59], we wondered whether BPA environmental exposure in fetal period will induce the excessive activation of primordial follicles through abnormal ERS in GCs of the newborn mice. To test this hypothesis, we first subcutaneously injected pregnant mice with BPA (10 μg/kg/day) from day 12.5 to 18.5 of gestation. The experimental arrangement was outlined in Fig. 6A. Results showed that fetal exposure to BPA resulted in an obvious encapsulation of activated oocytes with PARP1-positive GCs in the primary follicles in 4.5 dpp mouse ovaries of offspring (arrows in Fig. 6B). Of note, this treatment seemed to over-activate the dormant primordial follicles in the offspring ovaries (Fig. 6B, C), while the administration of NMS-P118 to the pregnant mice during the gestation day of 12.5 to 18.5 protected the hyperactivation of primordial follicles from BPA exposure (Fig. 6B, C). We then tested the expression of ERS-associated molecular markers in our experimental conditions, and found that these molecules were obviously increased in the 4.5 dpp mouse ovaries of offspring under fetal BPA exposure (Fig. 6D). Interestingly, PARP1 expression was also upregulated in BPA-treated group, compared to the controls (Fig. 6D), and suppression of PARylation with NMS-P118 treatment markedly impeded the upregulated expression of those ERS-associated molecules upon BPA exposure (Fig. 6D). These results suggested that fetal BPA exposure may accelerate PARP1-catalyzed protein PARylation and therefore induce ERS in the neonatal mouse ovaries, leading to the overactivation of primordial follicles in the offspring mice.

Melatonin is produced by the pineal gland and has been reported to be an ERS alleviator [60]. Interestingly, melatonin could effectively inhibit protein PARylation [61]. We thus hypothesized that melatonin may relieve the excessive ERS caused by fetal BPA exposure through inhibiting PARP1 enzymatic activity. As shown in Fig. 6E, F, melatonin treatment reversed BPA-triggered YY1 PARylation, and attenuated the upregulated ERS-related molecules under fetal BPA exposure. Accordingly, melatonin treatment restored the number of primordial follicles to that of the control group (Fig. 6G, H). Together, inhibiting the enzymatic activity of PARP1 can alleviate the overactivation of primordial follicles under fetal exposure to BPA.

## Discussion

It is generally accepted that the activation of primordial follicles is finely regulated by the ovarian microenvironment [7, 62]. And a couple of key molecules and signaling pathways have been identified in this process [2, 9]. Pioneering studies have reported that the surrounding somatic preGCs within the primordial follicles may initiate the activation of primordial follicles [7, 63], followed by the dramatic growth of the oocytes [63, 64]. Regarding the detailed molecular interaction between the somatic preGCs and the centered oocytes within the primordial follicles, the mTORC1-induced KITL expression in preGCs is believed to be the initial mediator, which then binds to KIT on the surface of the oocytes, activates the phosphatidylinositol 3-kinase (PI3K) signaling cascade, and awakens the dormant oocytes [7, 65]. However, the question why only a limited number of primordial follicles are selected to be recruited into the growing pool at a given time still remains to be answered. Here, we reported a novel role of PARP1 in the process of primordial follicle activation, whose catalytic activity seems to be unique in GCs and leads to the heterogeneity of postnatal ovarian follicle activation. We showed that PARP1 expression in the growing follicles is heterogeneous within the neonatal mouse ovaries. Those follicles with strong PARP1 expression in the surrounding GCs contribute to the development of growing follicles, while those with low PARP1 expression in preGCs stay quiescent, suggesting that PARP1 may be involved in the primordial follicle initial recruitment by regulating the proliferation and differentiation of GCs. It has been reported that the uncoupled development of oocyte and the surrounding GCs in an individual follicle will lead to the follicle development arrest during the mouse primordial-to-primary follicle transition [66, 67]. In consistence with these findings, our results showing that inhibition or depletion of PARP1 in GCs arrested GC proliferation and differentiation and led to the failure of primordial follicle activation may help establish PARP1 as a novel pivotal regulator in GCs in dominating the selection of individual follicles for survival.

Accumulating studies have showed that PARP1 catalyzes the transfer of ADP-ribose groups to a variety of nuclear protein acceptors, thereby affecting its target protein transcriptional activities, and eventually altering multiple biological processes, such as adipogenesis [27], inflammatory responses [28], tumorigenesis [29], and metabolic stress [68]. For example, PARylation of transcription factor C/EBPβ by PARP1 inhibits C/EBPβ’s DNA binding ability and transcriptional activity, and attenuates adipogenesis [68]. Similarly, PARylation of transcription factor STAT3 by PARP1 has been reported to inhibit STAT3 transcriptional activity, and decrease the expression of PD-L1 in cancer cells [29]. Conversely, PARylation of STAT1α by PARP1 on specific residues in its DNA-binding domain and transcription activation domain drives distinct STAT1α enhancer formation and transcriptional activation in regulating inflammatory responses [28]. These studies uncover a molecular and cellular context-dependent role for PARP1-mediated PARylation in regulation of gene transcription. In our study, we found that PARP1-catalyzed transcription factor YY1 PARylation at Y185 residue increased YY1 occupancy at the *Grp78* promoter and promoted *Grp78* transcription in GCs during primordial follicle activation. Though previous studies have reported the presence of PARylation of YY1 under DNA damage in tumor cells, the sites of PARylation were not identified [52, 69]. A recent comprehensive ADP-ribosylome analysis confirmed tyrosine (Y) as an ADP‐ribose‐acceptor amino acid, and Y185 residue in YY1 was potentially ADP-ribosylated [55]. However, this site was not validated yet. We provided the first evidence showing that mutation of Y185 residue in YY1 abolished YY1 PARylation and thus decreased YY1 occupancy at the *Grp78* promoter, suggesting that Y185 residue in YY1 may represent a functional site of PARP1-mediated PARylation in neonatal mouse follicles.

The transactivation of *Grp78* is widely used as a biomarker for onset of the UPR of the endoplasmic reticulum [51]. YY1 is an essential transcription factor that can bind to the ER stress response element (ERSE) of *Grp78* promoter [70, 71]. Interestingly, YY1 only occupies the *Grp78* promoter and activates *Grp78* transcription in cells subjected to ER stress, while it has no effect on the basal activity of *Grp78* promoter despite its constitutive expression [70, 71]. The underlying molecular mechanism of this selective activation of *Grp78* promoter by YY1 is not clear. Previous studies proposed that YY1 may form a complex with the nuclear form of ATF6 on the *Grp78* promoter, which confers a higher stability to bind to ERSE of *Grp78* promoter, or YY1 may recruit chromatin-modifying enzymes to alter chromatin structure and thus enhance YY1 binding in response to ER stress [51, 72]. In our studies, we established the first link of YY1 PARylation by PARP1 to *Grp78* promoter occupancy, which may provide an important clue to elucidate the mechanisms for the activation of the *Grp78* promoter under ER stress. In fact, another crucial protein of the PARP family member, PARP16, has also been reported to regulate ADP-ribosylation of the key enzymes in the ERS response pathway, such as PERK and IRE1α during the UPR. This modification on these two ER stress sensors by PARP16 increases their kinase activities [73]. Therefore, we cannot exclude the possibility that PARP1 may also participate in the PARylation of these ER stress sensors, which needs further exploration in the future.

To meet the rapidly increased needs of protein demand in the growing follicles, maintenance of ER homeostasis by the UPR is critically required for folliculogensis and oocyte maturation [74, 75]. However, whether ERS at physiological level is associated with primordial follicle activation remains unclear. One study in *Galactose-1 phosphate uridylyltransferase*-deficient (*GalT*^*−/−*^) mouse model did show that *GalT*^*−/−*^ ovaries displays elevated ERS markers, accompanied by a substantial reduction in the number of primordial follicles. When treating the mutant mice with a chemical compound that alleviates ER stress, they found that the compound significantly slows down the premature loss of primordial ovarian follicles [76]. This is the first report to link the ERS to the primordial follicle activation. Consistent with this finding, our study clearly demonstrates that GCs in the growing follicles actually experience ERS in the process of primordial follicle activation, and PARP1-mediated such ERS in GCs determines the subpopulation of the dormant primordial follicles to be awakened (Fig. 6I). This may represent a novel determinant for the selective activation of primordial follicles, which greatly help us better understand the mystery of primordial follicle activation.

Since ERS at physiological level plays a crucial role during primordial follicle activation, it is conceivable that additional interference to ER homeostasis in pathological condition would bring a detrimental effect to this process. For example, fatty acid- or obesity-induced perturbations to ERS have been shown to impair protein secretion and mitochondrial activity in mouse cumulus-oocyte complexes [77, 78]. Consistently, our study also demonstrated that BPA environmental exposure in fetal period over-activated primordial follicles in the newborns through excessive activation of ERS, in which PARP1-catalyzed YY1 PARylation is strictly involved. More importantly, either inhibiting PARP1 catalytic activity with NMS-P118 or administration of melatonin showed a protective effect on the loss of primordial follicles by attenuation of ERS in the newborn ovaries under fetal BPA exposure. These results unravel how PARP1-catalyzed YY1 PARylation in GCs contributes to the regulation of primordial follicle activation through balancing ERS. Our findings also identify a role for melatonin in the protection of fetal BPA exposure-induced excessive ERS and primordial follicle overactivation, and support the concept that PARP1 in GCs may represent a pivotal modulator to determine the fate of the primordial follicles.

## Materials and methods

### Mice and treatments

The Animal Care and Use Committee of Nanjing Medical University approved all animal procedures. The mice were housed at 22–24 °C, and exposed to a 12 h dark/12 h light cycle with free access to food and water. Mice were mated overnight. Females were checked for vaginal plug in the next morning. The presence of vaginal plug was recorded as 0.5 days postcoitus (dpc). Neonatal ovaries were collected at 0.5, 2.5, 4.5, and 7.5 days postpartum (dpp). For drug treatment, all pregnant mice were randomly divided into control and experimental groups (*n* = 4/group). The pregnant mice were subcutaneously injected with BPA (#R005000, RHAWN, Shanghai, China) (10 μg/kg/day) from 12.5 dpc to 18.5 dpc. And PBS was administered in the control group. Melatonin (50 mg/kg/day, #HY-B0075, MedChemExpress, New Jersey, USA) or NMS-P118 (10 mg/kg/day, #HY-18954, MedChemExpress) was intragastrically administrative from day 12.5 to 18.5 of pregnancy. Neonatal ovaries were collected at 4.5 dpp.

### Ovary culture

Neonatal ovaries were collected at 2.5 dpp, and 4 ovaries were randomly selected as a group and cultured in a 24-well dish with α-minimal essential medium (α-MEM, #12571063, Invitrogen, Carlsbad, CA, USA) supplemented with 5% FBS, 1% penicillin/streptomycin and ITS at 37 °C under 5% CO_2_. Half of the medium was replaced every two days. For the ovaries treated with 50 nM of Tunicamycin (TM, #HY-A0098, MedChemExpress), or 50 μM of Tauroursodeoxycholate (TUDCA, #HY-19696, MedChemExpress), dimethylsulfoxide (DMSO) was used as a control. The ovary transfection was performed as described in the previous studies [79, 80]. Briefly, the ovaries were cultured in a 24-well plate for 30 min, followed by transfection with 4 μl of 50 μM siRNA against *Parp1* (General Biosystems, Anhui, China) and 1 μl of Hieff Trans^TM^ (#40802ES02, YEASEN, Shanghai, China) in 200 μl of culture medium at 37 °C for 24 h. After 3 days of culture, the ovaries were collected. The siRNA oligonucleotide sequences were listed in Supplementary Table 1.

### Histological evaluation of follicle numbers

The ovaries were fixed overnight at 4 °C in 4% paraformaldehyde, embedded in paraffin, and serially sliced into 5-µm-thick sections. The sections were stained with hematoxylin dye and observed under a light microscope (ECLIPSE Ni-E, Nikon Instruments, Tokyo, Japan). The follicles in every fifth section were counted. Only those follicles containing clearly visible oocyte nuclei in each section of the ovaries were counted. The follicles were distinguished from each other as follows: primordial follicle (a single oocyte surrounded by several flattened pre-granulosa cells) and primary follicle (an enlarged oocyte surround by a mixture of squamous and cuboidal granulosa cells). To estimate the total number of follicles at different development stages in each ovary, the sum was multiplied by five.

### Immunoblotting

Total ovary lysates were separated on SDS-PAGE. After electrophoresis, the protein was transferred to PVDF membrane. The membranes were blocked with 5% skimmed milk and incubated with anti-GRP78 (1:1000, #11587–1-AP, Proteintech, Hubei, China), anti-PARP1 (1:4000, #66520-1-lg, Proteintech), anti-PAR (1:1000, #4335-MC-100, Bio-techne, Minneapolis, USA), anti-mTOR (1:1000, #20657-1-AP, Proteintech), anti-p-mTOR (1:800, #sc-293133, Santa cruz, Dallas, Texas, USA), anti-KitL (1:1000, #bs-0545R, Biorbyt, Hubei, China), anti-p-IRE1α (1:800#, bs-16698R, Biorbyt), anti-IRE1α (1:1000, #27528-1-AP, Proteintech), anti-ATF4 (1:1000, #10835-1-AP, Proteintech), anti-p-PERK (1:800, #Orb336657, Biorbyt), anti-PERK (1:1000, #24390-1-AP, Proteintech), anti-HA-tag (1:1000, #30701ES60, YEASEN), anti-FLAG-tag (1:1000, #30501ES60, YEASEN) and anti-GAPDH (1:4000, #ab8245, Abcam, Cambridge, UK) antibodies overnight at 4 °C. After TBST washing, the membranes were treated with the corresponding peroxidase-conjugated secondary antibody, and then the signal was detected with an Enhanced Chemiluminescence Detection Kit (#32106, Thermo Scientific, Waltham, MA, USA) on Tanon 4500 gel imaging system (Shanghai, China).

### Immunofluorescence staining

The ovarian sections were subjected to antigen retrieval in EDTA solution in boiling water for 15 min. The sections were then blocked for 30 min in 10% normal goat serum at room temperature. Primary antibody binding was performed overnight at 4 °C, including anti-DDX4 (1:200, #Ab13840, Abcam), anti-DDX4 (1:200, #Ab27591, Abcam), anti-GRP78 (1:200, #11587-1-AP, Proteintech), PARP1 (1:400, #66520-1-lg, Proteintech), anti-FOXL2 (1:200, #19672-1-AP, Proteintech), and anti-ATF4 (1:200, #10835-1-AP, Proteintech). After 5 washes in PBST, the sections were then stained with the corresponding fluorescence-labeled secondary antibody for 60 min at room temperature. The nuclei were counterstained with DAPI. The sections were examined under a confocal laser scanning microscope (LSM 700; Zeiss, Oberkochen, Germany).

### Isolation of total RNA and quantitative real-time RT-PCR

Total RNA from mouse ovaries was extracted by TRIzol reagent (#15596026, Invitrogen). Total RNA (1.0 μg) was treated with RNase-free DNase, and then reverse transcribed with reverse transcriptase (K1621, Thermo Scientific, Waltham, MA, USA). Real-time RT-PCR was performed with AceQ qPCR SYBR Green Master Mix (#Q141-02, Vazyme, Jiangsu, China). All primers were presented in supplementary Table 2. The comparative CT method was used to determine the amount of target, normalized to an endogenous reference (*Gapdh*) and relative to a calibrator (2^−ΔΔCt^).

### Oocyte/ovarian somatic cell (OSC) isolation and in vitro reconstruction

The in vitro reconstruction procedure was modified as described previously [81]. Briefly, at least 15 fetal ovaries at 2.5 dpp were harvested and treated with NMS-P118 or DMSO for one day, and washed in 200 µl of PBS to rinse off the medium. Ovaries were then incubated with 200 µl of 0.25% trypsin solution at 37 °C for 15–20 min until complete tissue digestion, followed by addition of an equal volume of 10% FBS to terminate tissue digestion. The cell suspension was centrifuged at 1500 ×g for 5 min at 4 °C. The cell pellets were resuspended in PBS and filtered through a 50-µm-diameter cell sieve. The filtered cells were collected by centrifugation and resuspended in an ovarian in vitro culture media. Then, 1.5 × 10^5^ of cells suspended in α-MEM/ITS were added to a 6-well dish. After 6 h of cell culture, the oocyte/ovarian somatic cell (OSCs) adhered to the culture plates, while the oocytes still remained in the media. Therefore, the oocytes were separated from the OSCs by directly remove the media, and the remaining OSCs were recovered by digestion. These two types of cells from different treatment of ovaries were reconstructed with phytohemagglutinin, and cultured in 24-well plates for 12 h. Finally, the cell clumps were carefully transplanted under the kidney of ovariectomized adult recipient mice (8-weeks-old) for 14 days.

### Chromatin immunoprecipitation (ChIP)

MAGNA ChIP kit (#17371RF, Millipore, USA) was used to perform the ChIP assay. Cross-linked chromatin from mouse ovaries was prepared and immunoprecipitations were performed as instructed using the antibodies, including anti-PARP1 (4 µg/sample, #66520-1-lg, Proteintech), anti-YY1 (4 μg/sample, #22156-1-AP, Proteintech), or rabbit IgG (4 μg/sample, #12–370, Millipore, USA). The enriched chromatin DNA was amplified by PCR and the product was separated electrophoretically on agarose gel. Primers are listed in supplementary Table 3.

### Cell transfection

Human embryonic kidney (HEK) 293 cells were purchased from the Cell Resource Center of the Shanghai Institute for Biological Sciences (Shanghai, China). Cells were cultured in Dulbecco’s Modified Eagle’s medium (DMEM; HyClone, USA) with 10% FBS and 1% penicillin/streptomycin in a maintained chamber with 5% CO_2_. Cells were transfected with HA tagged-PARP1 and FLAG tagged-YY1 plasmids using Hieff Trans^TM^ reagent for 40 h, followed by cell collection. The mutation of PARP1 and YY1 were performed by site-directed mutagenesis (Mut Express II Fast Mutagenesis Kit V2, #C214, Vazyme) and verified by sequencing.

### Cellular and ovarian PARylation detection

HEK293 cells transfected with HA-PARP1 and Flag-YY1 plasmids, or the cultured ovaries treated with NMS-P118 or si*Parp1* were lysed in 4% SDS buffer. The tPARylation detection were carried out as previously described [29]. Briefly, the cellular or ovarian lysates were immunoprecipitated with the anti-FLAG antibody, anti-YY1, or anti-GRP78 antibody, respectively. The poly(ADP-ribosyl)ated YY1 proteins were detected using western blot with an anti-PAR primary antibody at a dilution of 1:10000.

### Statistical analysis

All experiments were repeated at least three independent biological replicates and presented as the means ± S.D. Statistical analysis was performed using Student’s *t* tests or one-way *ANOVA* to compare the difference. Differences were considered significant at *P < 0.05, **P < 0.01, and ***P < 0.001.

### Supplementary information

aj-checklist

Primers

Original Data File

## Data Availability

The materials described in the manuscript can be provided upon request.
