# Correction: PARP1-catalyzed PARylation of YY1 mediates endoplasmic reticulum stress in granulosa cells to determine primordial follicle activation

**DOI:** 10.1038/s41419-023-06152-w

**Published:** 2023-10-05

**Authors:** Wei Chen, Qiukai E, Bo Sun, Pengxue Zhang, Nan Li, Shujia Fei, Yingnan Wang, Shuting Liu, Xiaoqiu Liu, Xuesen Zhang

**Affiliations:** 1 State Key Laboratory of Reproductive Medicine, Nanjing Medical University, Nanjing, 211166 China; 2 Department of Obstetrics and Gynecology, the Affiliated Jiangning Hospital of Nanjing Medical University, 211166 Nanjing, China; 3 Department of Pathogen Biology, College of Basic Medical Sciences, China Medical University, Shenyang, 110122 China; 4 Key Laboratory of Human Functional Genomics of Jiangsu Province, Nanjing Medical University, Nanjing, 211166 China; 5 College of Basic Medical Science, China Medical University, Shenyang, 110122 China

**Keywords:** Oogenesis, Mechanisms of disease

Correction to: *Cell Death and Disease* (2023) 10.1038/s41419-023-05984-w, published online 15 August 2023

In this article the affiliation of author Xuesen Zhang is given as:

College of Basic Medical Science, China Medical University, Shenyang, 110122, China.

Nanjing Medical University, Key Laboratory of Human Functional Genomics of Jiangsu Province, Nanjing, 211166, China.

It should read:

Key Laboratory of Human Functional Genomics of Jiangsu Province, Nanjing Medical University, Nanjing, 211166, China.

College of Basic Medical Science, China Medical University, Shenyang, 110122, China.

The original article has been corrected.

