# Supplementary material for: PARP1-catalyzed PARylation of YY1 mediates endoplasmic reticulum stress in granulosa cells to determine primordial follicle activation
Source: Cell Death Dis. 2023 Aug 15;14(8):524. doi: 10.1038/s41419-023-05984-w (PMC10427711; doi:10.1038/s41419-023-05984-w)
Supplement: Supplementary file 2 — Primers [file 41419_2023_5984_MOESM2_ESM.docx]

**SUPPLEMENTAL INFORMATION**

**Supplementary Table 1. siRNA oligonucleotide sets**

| **Gene** | **Primer** | **Sequence (5′-3′)** |
| --- | --- | --- |
| si*NC* | Forward | UUCUCCGAACGUGUCACGUTT |
|  | Reverse | ACGUGACACGUUCGGAGAATT |
| si*Parp1*-1 | Forward | CCAAAGGAAUUCCGAGAAATT |
|  | Reverse | UUUCUCGGAAUUCCUUUGGTT |
| si*Parp1*-2 | Forward | AGAAUGAAGGAAAGAGAAATT |
|  | Reverse | UUUCUCUUUCCUUCAUUCUTT |
| si*Yy1* | Forward | AGAAGCAGGUGCAGAUCAATT |
|  | Reverse | UUGAUCUGCACCUGCUUCUTT |

**Supplementary Table 2. Primers for RT-qPCR**

| **Gene** | **Primer** | **Sequence (5′-3′)** |
| --- | --- | --- |
| *Gapdh* | Forward | AGGTTGTCTCCTGCGACTTCA |
|  | Reverse | GGGTGGTCCAGGGTTTCTTACT |
| *Parp1* | Forward | GGACGAAGAGGCAGTAAAGAAG |
|  | Reverse | CTCGCTGAGGTAAGAGTAGGC |
| *Gdf9* | Forward | GGCGATAAGTTGTAGGAGATTAC |
|  | Reverse | GAAAATAACGAACGAAAACCG |
| *Bmp15* | Forward | TCCTTGCTGACGACCCTACAT |
|  | Reverse | TACCTCAGGGGATAGCCTTGG |
| *Grp78* | Forward | CTGGCCGAGACAACACTGACCT |
|  | Reverse | GCGACGACGGTTCTGGTCTCAC |
| *Atf6* | Forward | TCGCCTTTTAGTCCGGTTCTT |
|  | Reverse | GGCTCCATAGGTCTGACTCC |
| *Atf4* | Forward | TATGGATGATGGCTTGGCCAG |
|  | Reverse | TTCCAGGTCATCCATTCGAAAC |
| *Yy1* | Forward | AAAGCATCTGCACACCCACG |
|  | Reverse | CTCCGGTATGGATTCGCACA |

**Supplementary Table 3. Primers for ChIP-qPCR**

| **Name** | **Primer** | **Sequence (5′-3′)** |
| --- | --- | --- |
| PARP1-GRP78-1 | Forward | TAAATGGCCCAAGTAGCCTTCA |
|  | Reverse | ACACAACTGACATCTGTCTCC |
| PARP1-GRP78-2 | Forward | TTGTTTTGTTTGTCCCCCAACAT |
|  | Reverse | CGACTCTTGGAATAGGGCTGA |
| PARP1-GRP78-3 | Forward | GGGGAGGACCTGAACGGTTA |
|  | Reverse | GAGCGCTGGTCCTATTGGTT |
| YY1-GRP78 | Forward | CATTGGTGGCCGTTAAGAATGAC |
|  | Reverse | AGTATCGAGCGCGCCGTCGC |
