# Supplementary material for: PARP1-catalyzed PARylation of YY1 mediates endoplasmic reticulum stress in granulosa cells to determine primordial follicle activation
Source: Cell Death Dis. 2023 Aug 15;14(8):524. doi: 10.1038/s41419-023-05984-w (PMC10427711; doi:10.1038/s41419-023-05984-w)
Supplement: Supplementary file 3 — Original Data File [file 41419_2023_5984_MOESM3_ESM.doc]

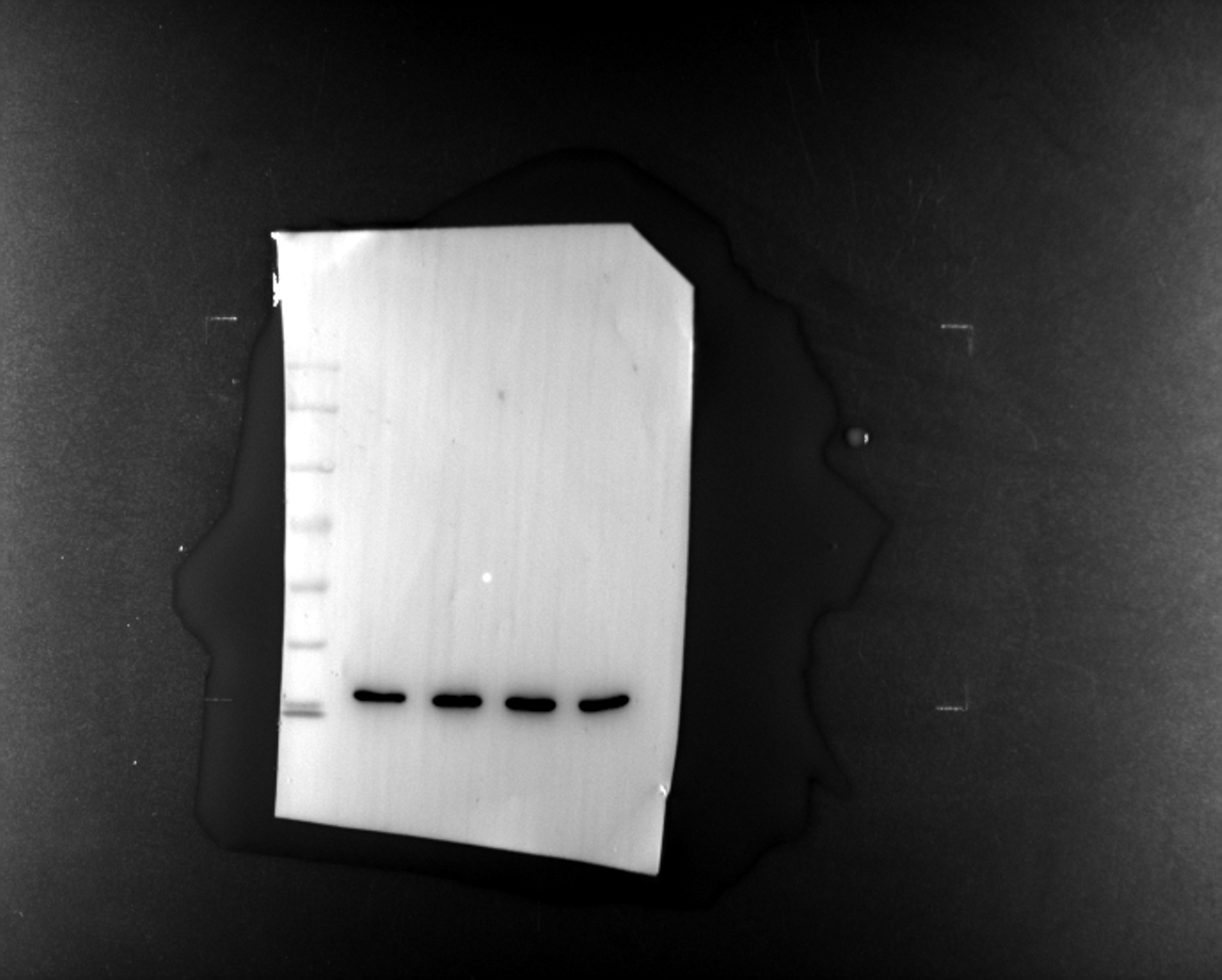

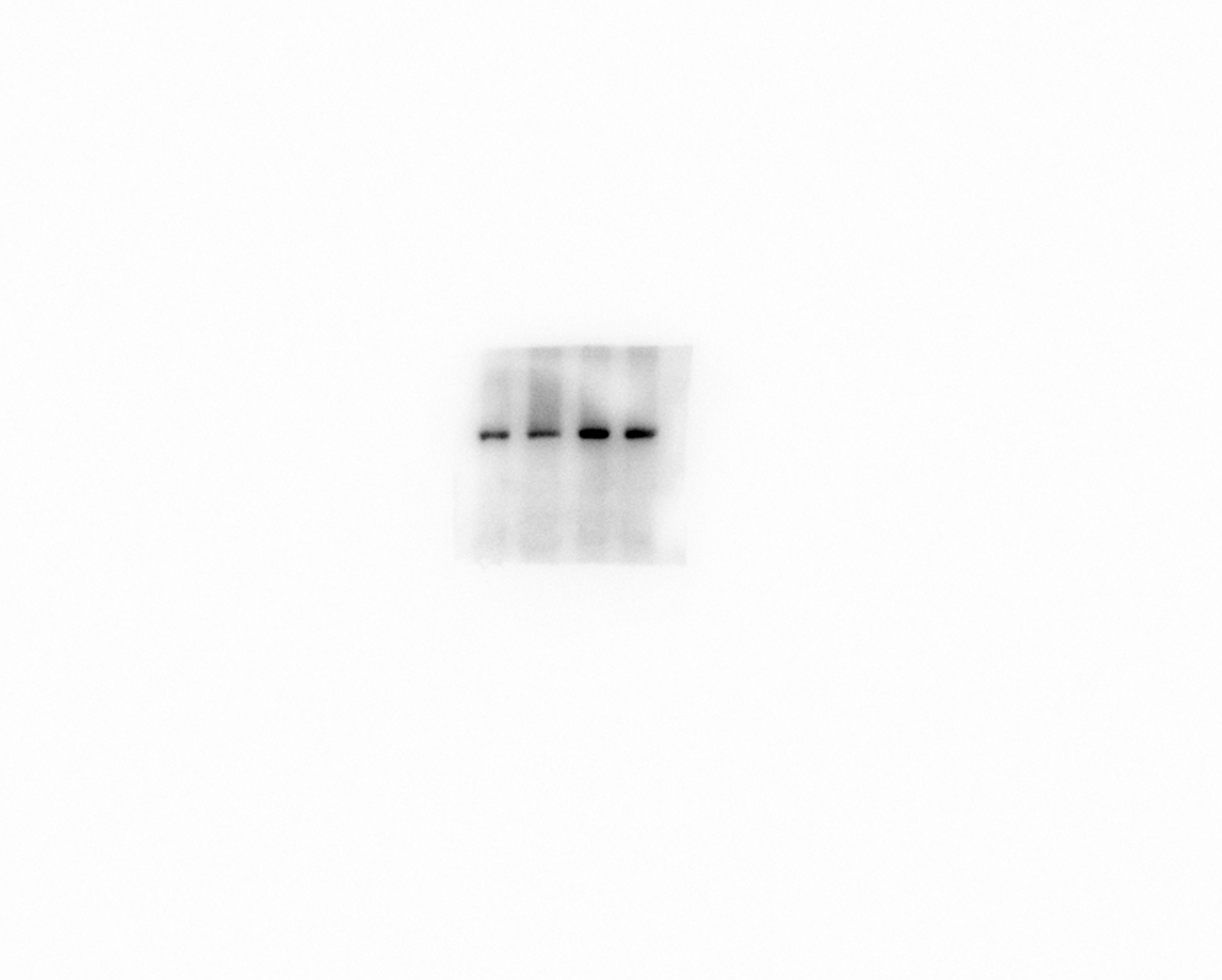


Figure 1B-GAPDH Figure 1B-PARP1


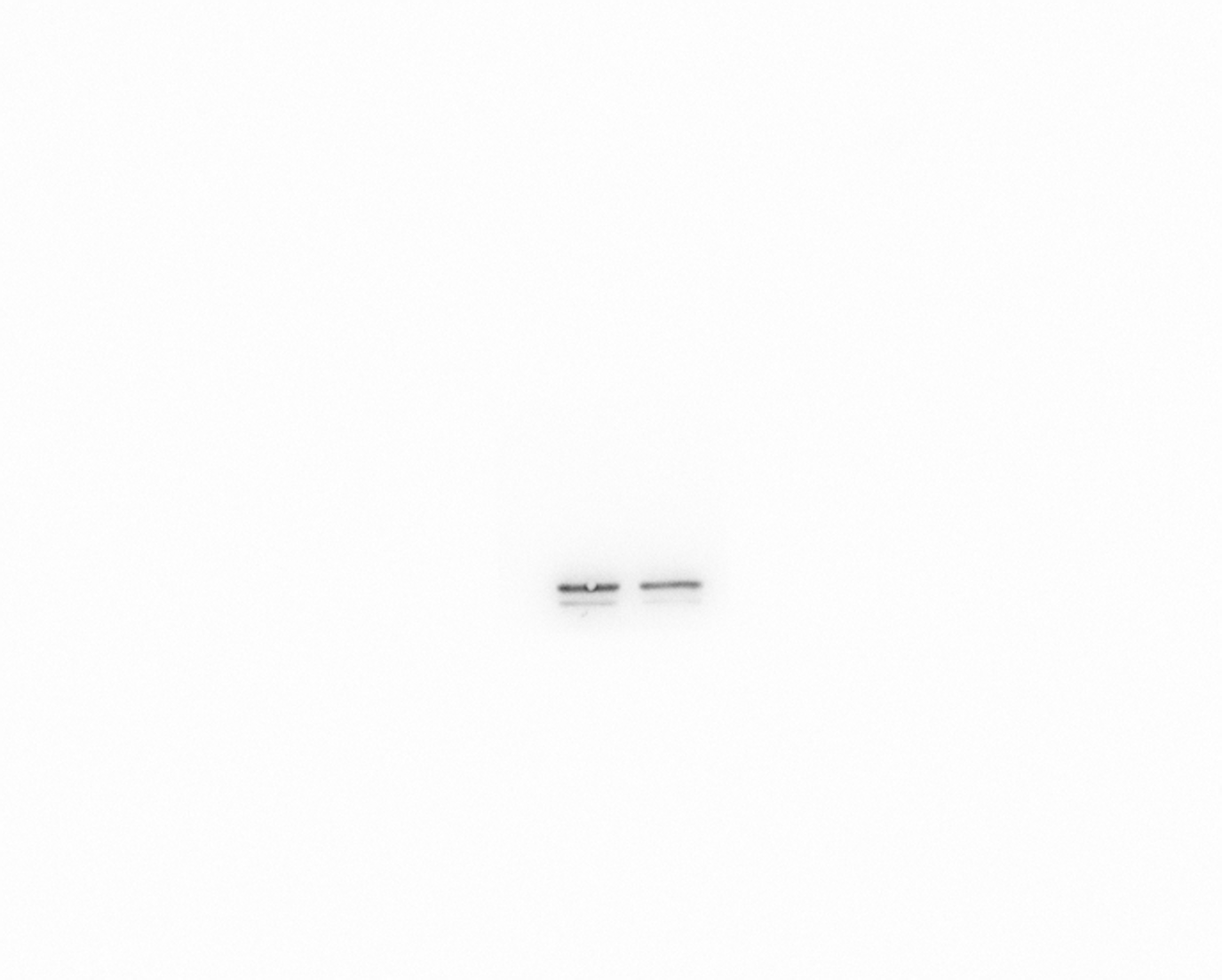

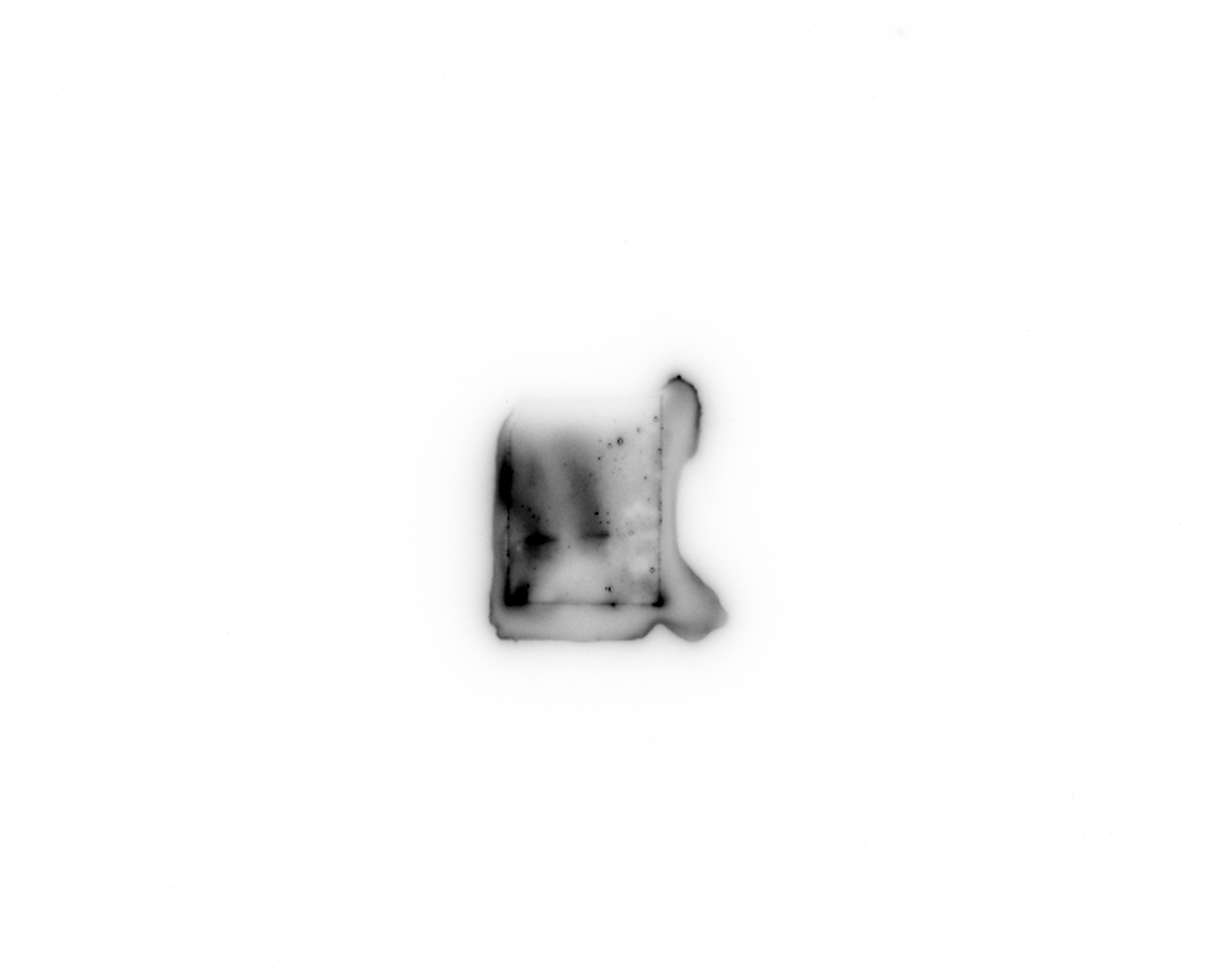

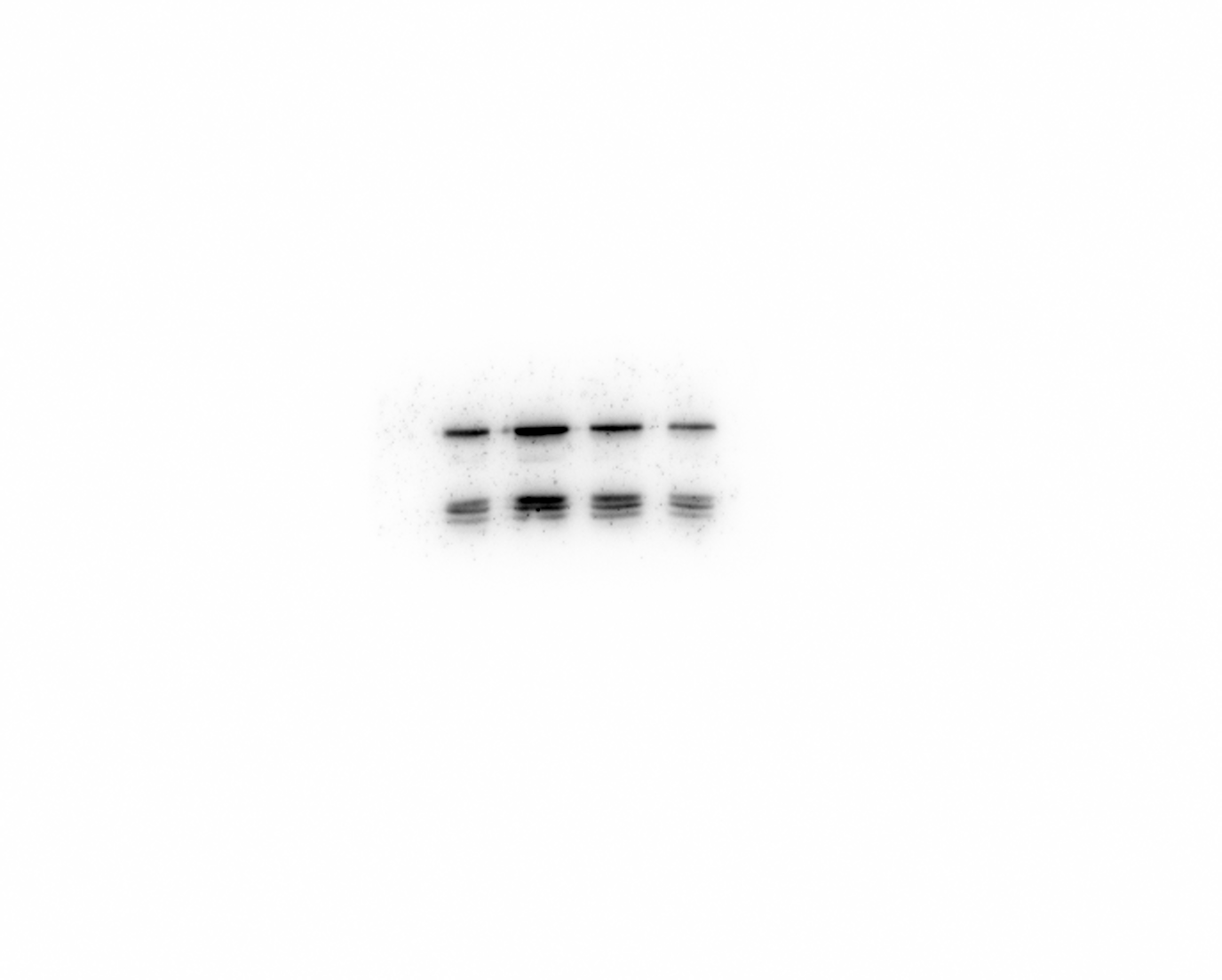


Figure 1D-GAPDH Figure 1D-PAR Figure 1D-PARP1


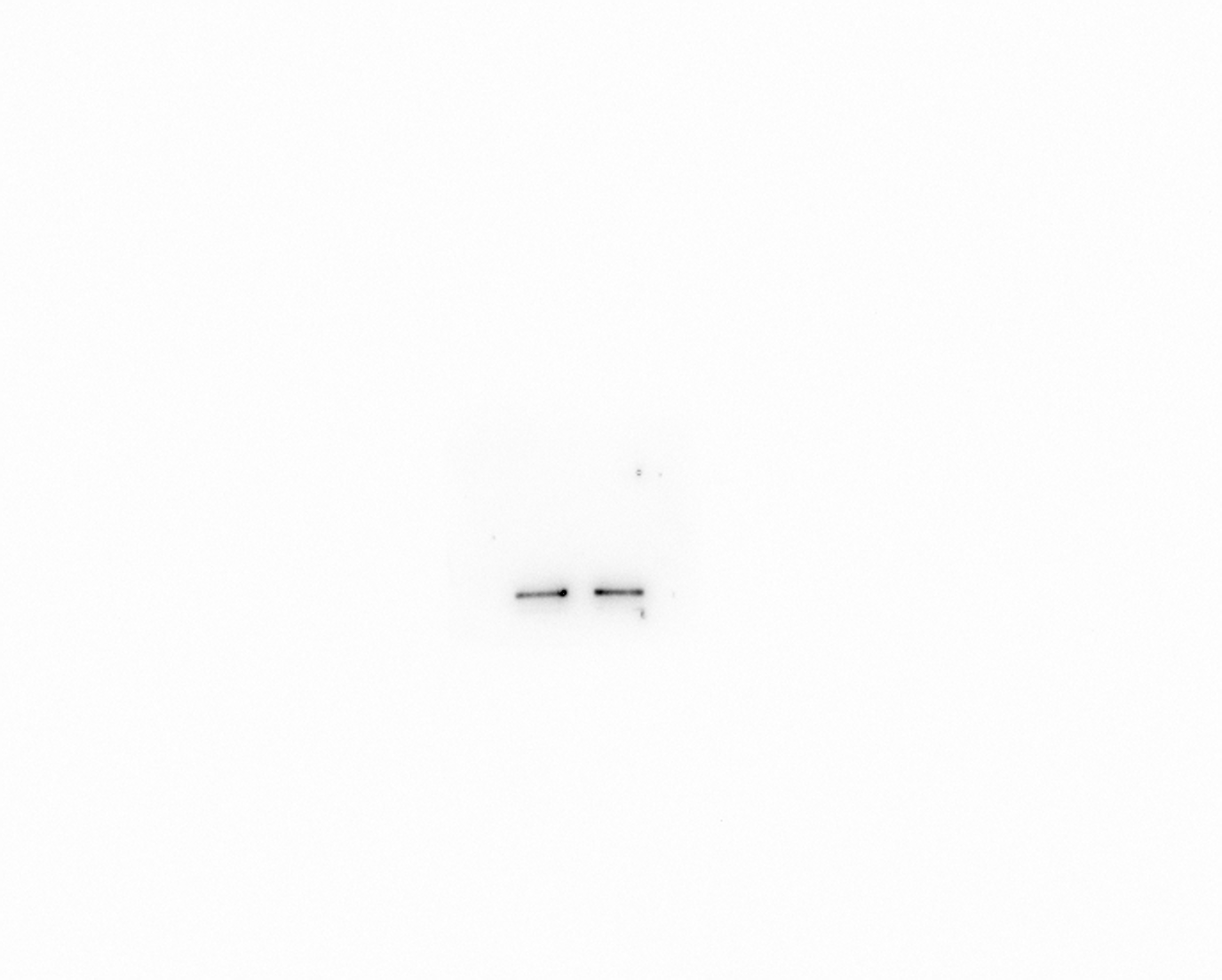

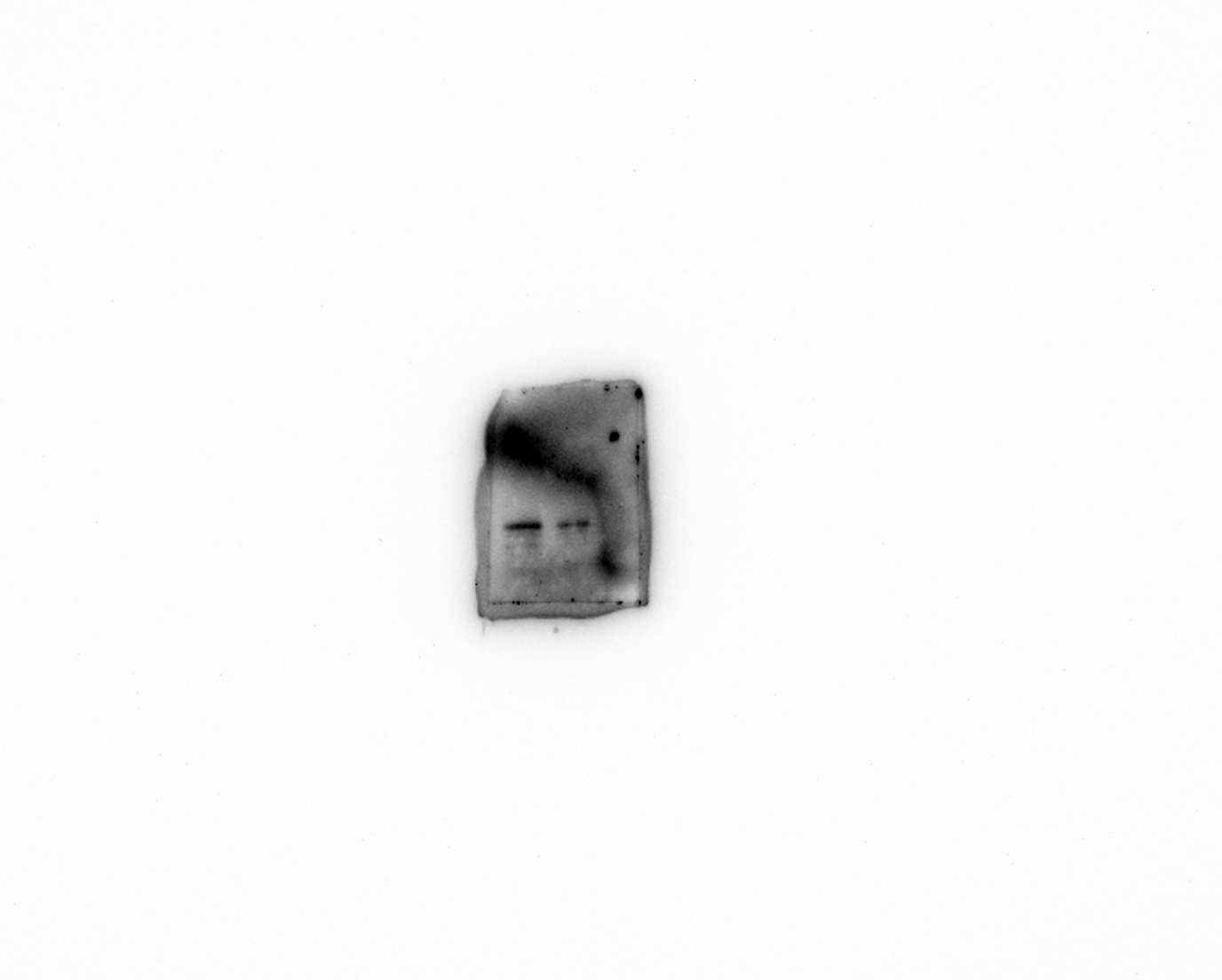

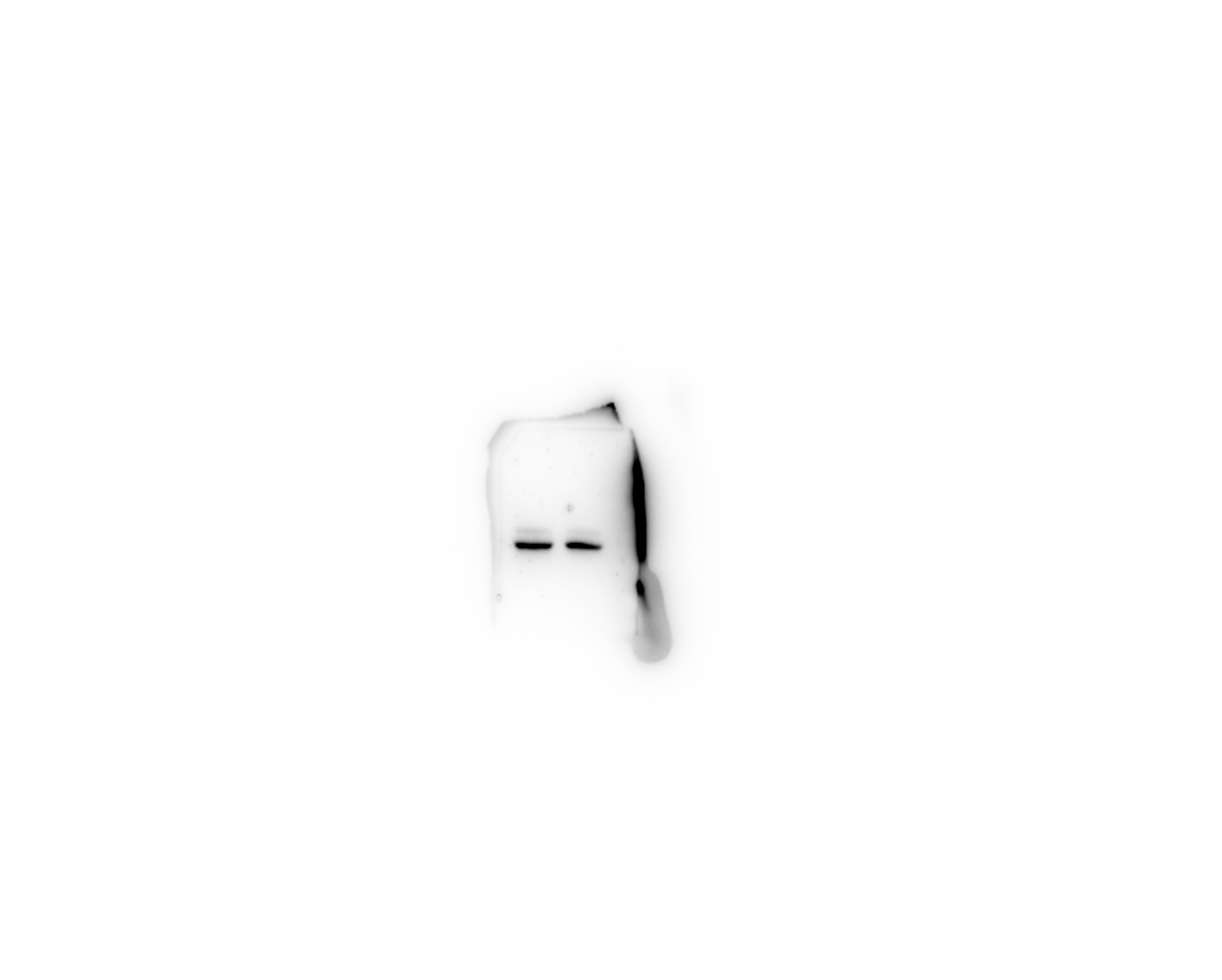


Figure 1F-GAPDH Figure 1F-PAR Figure 1F-PARP1


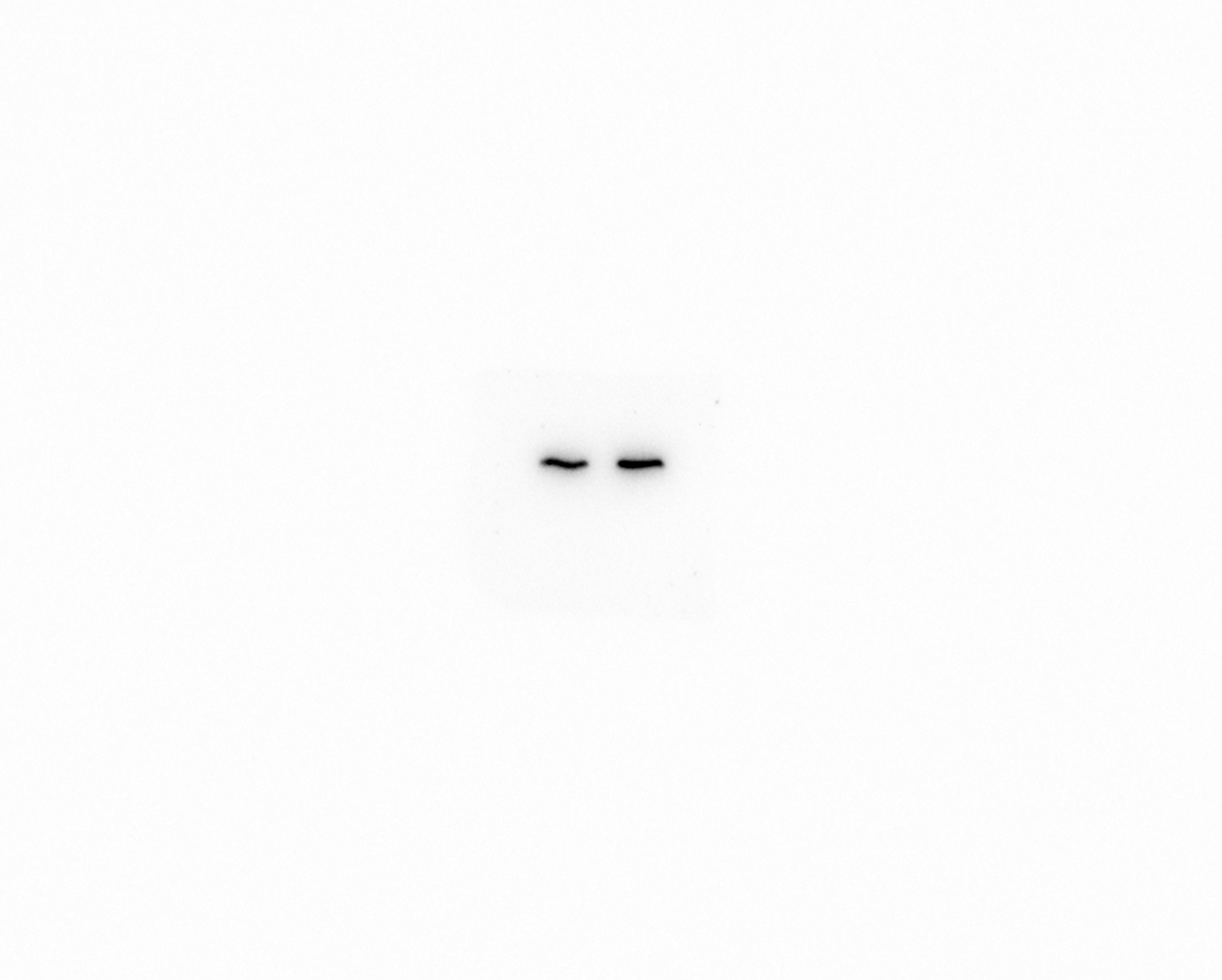

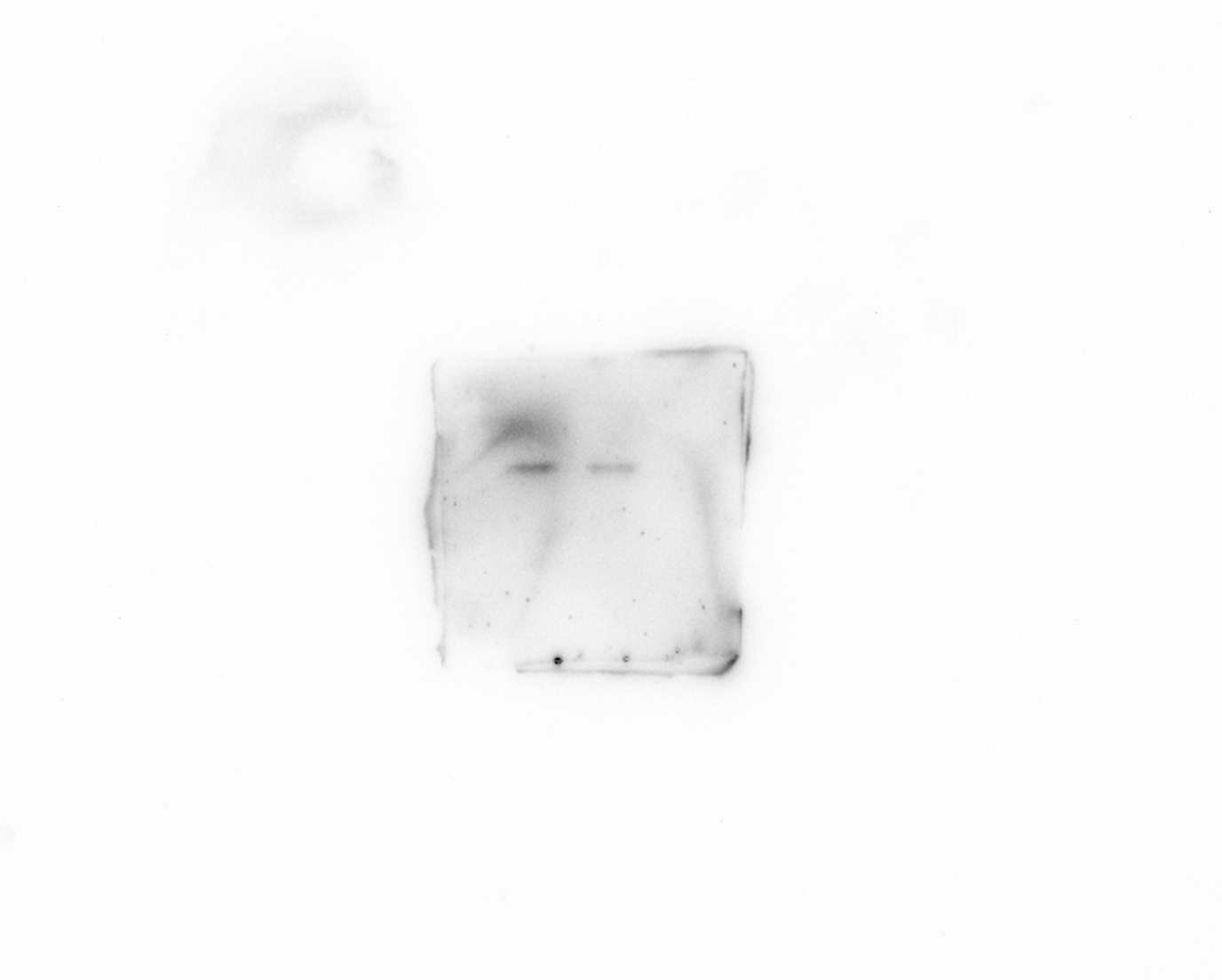

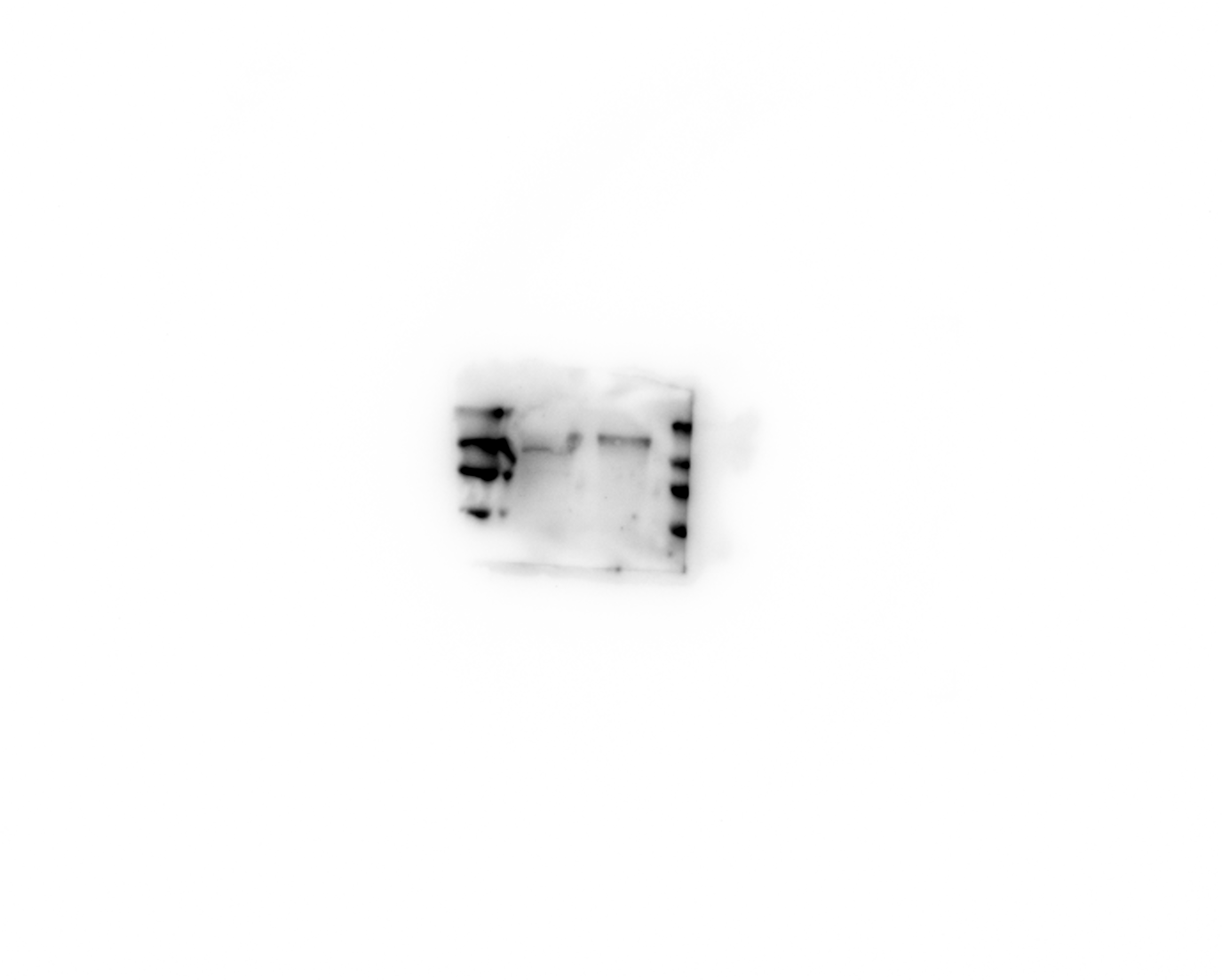

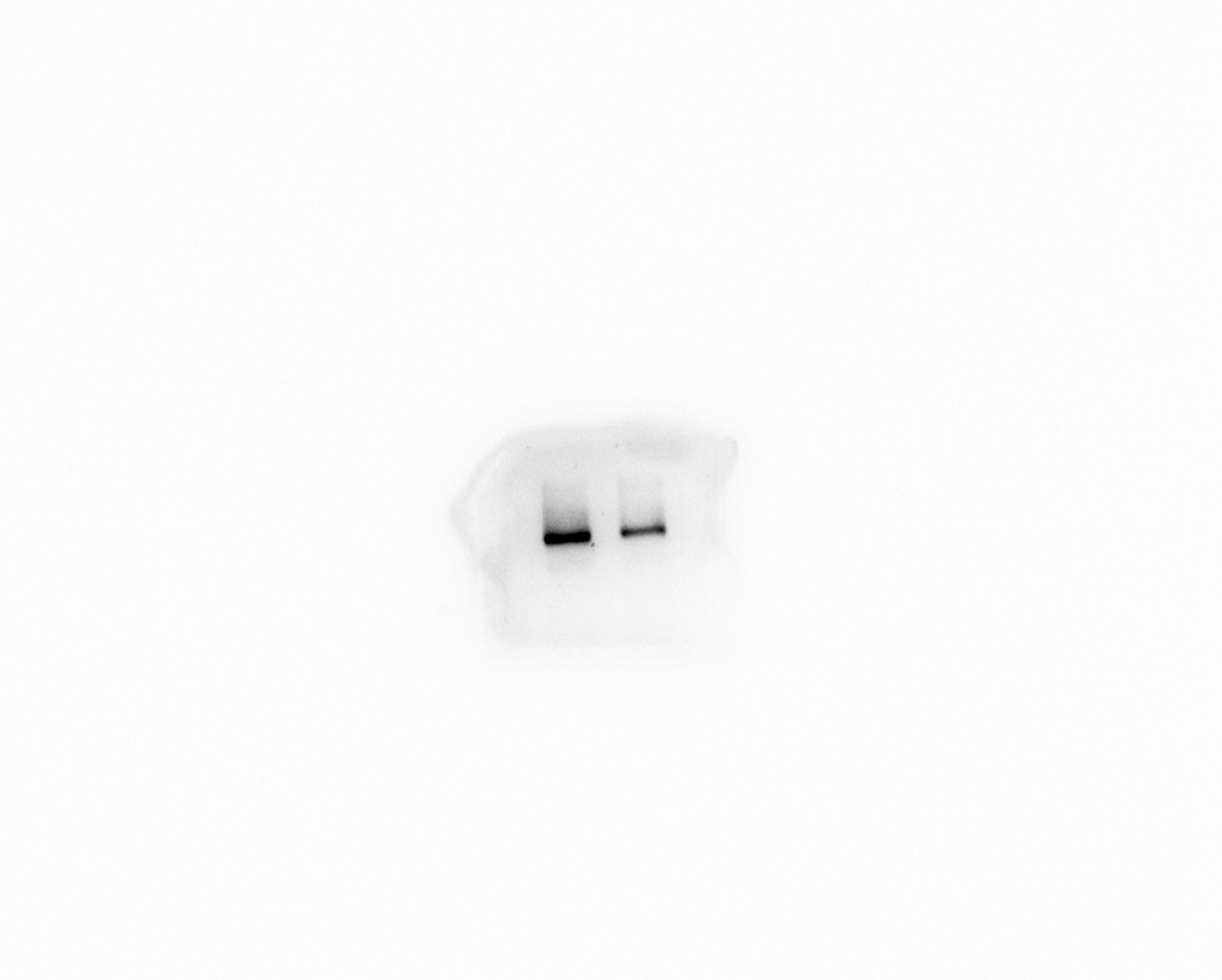

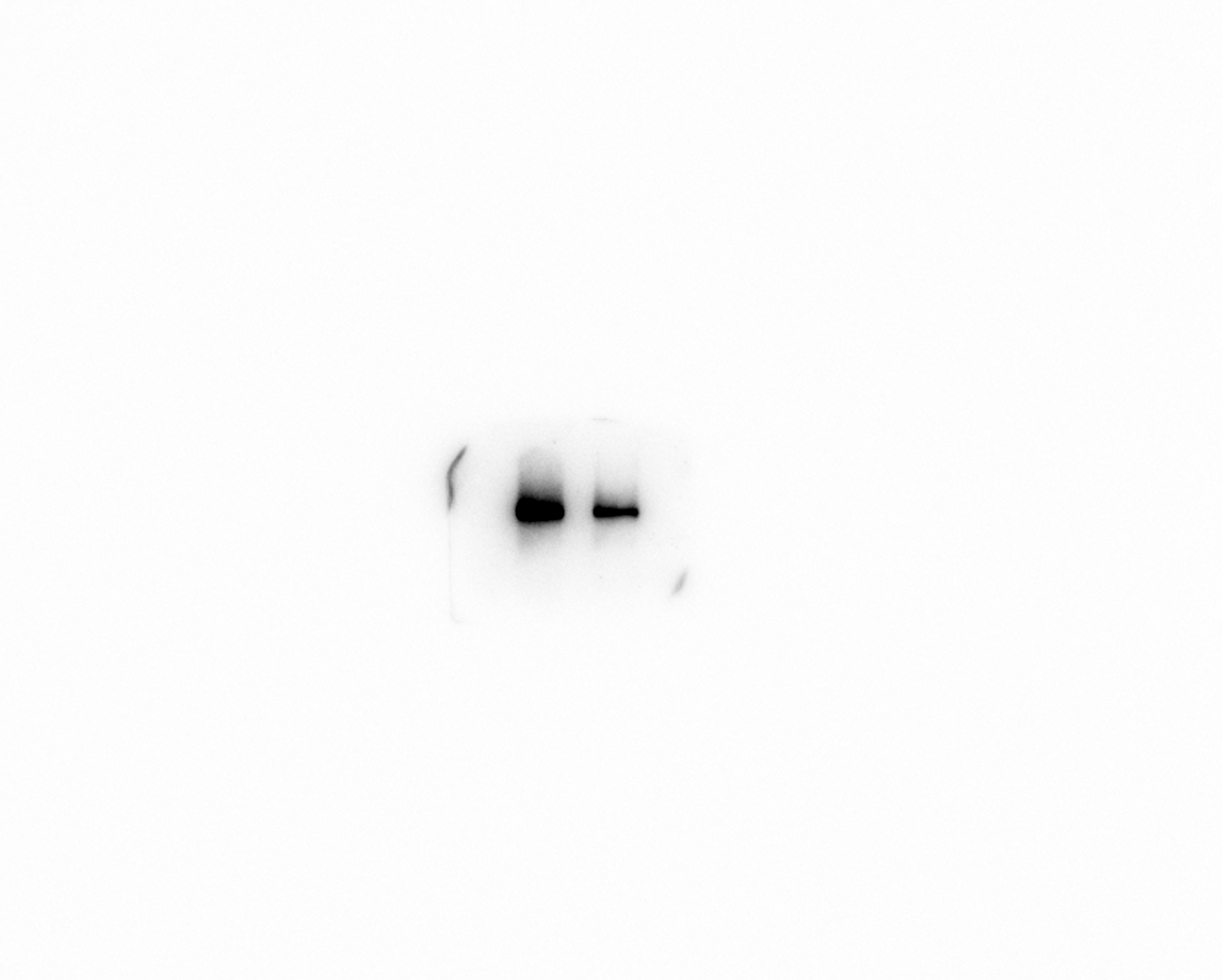


Figure 2E-GAPDH Figure 2E-KITL Figure 2E-mTOR Figure 2E-PARP1 Figure 2E-pmTOR


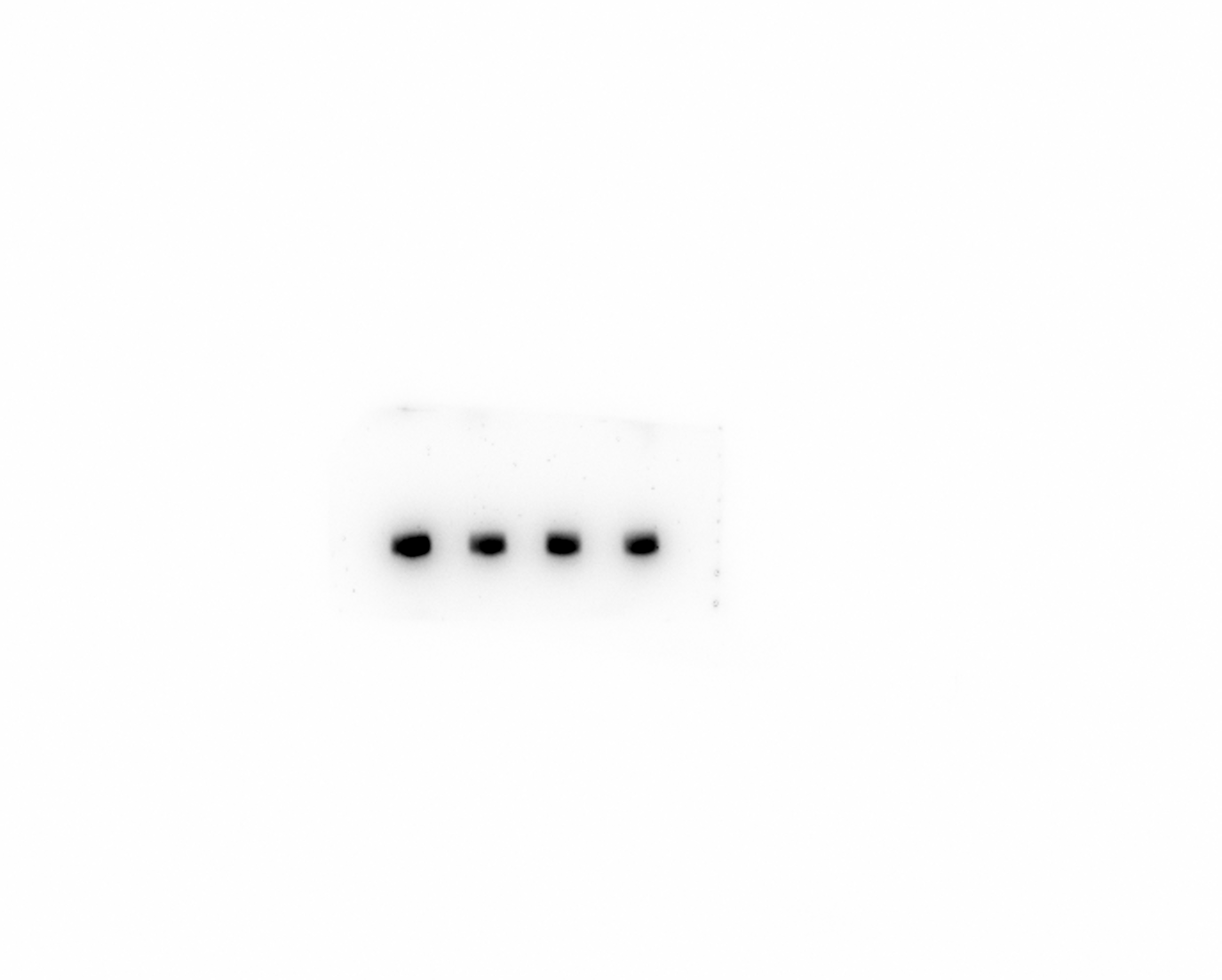

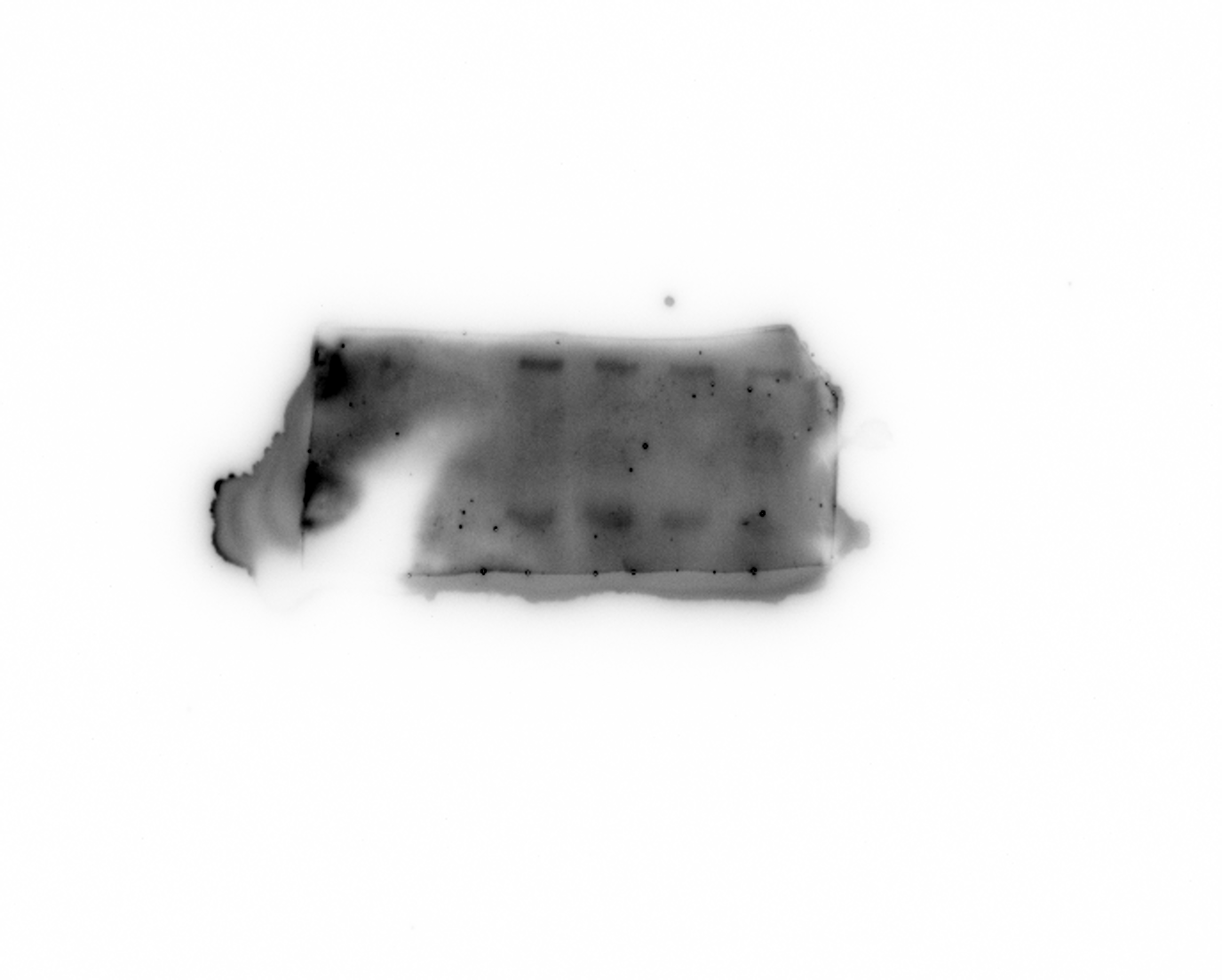

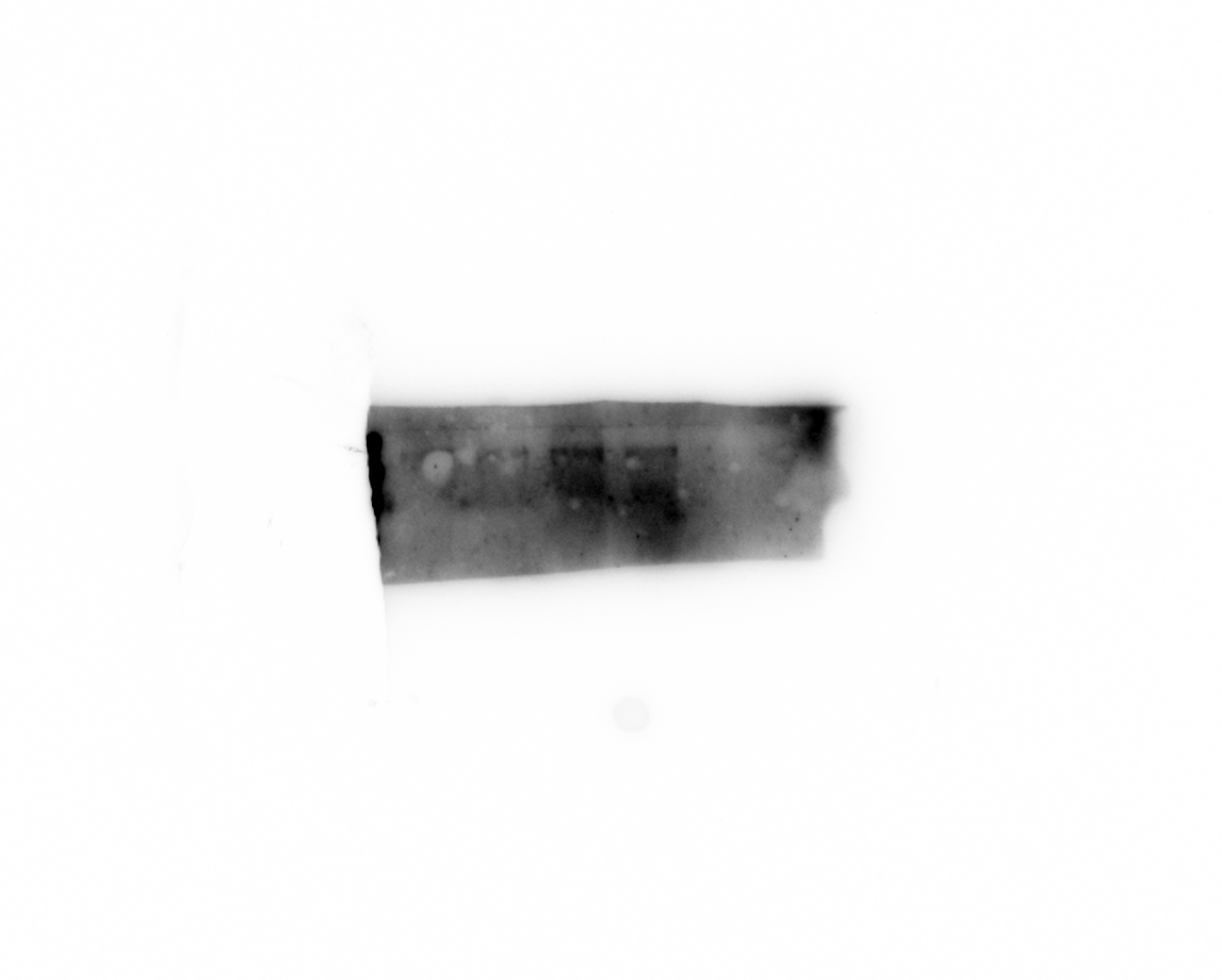

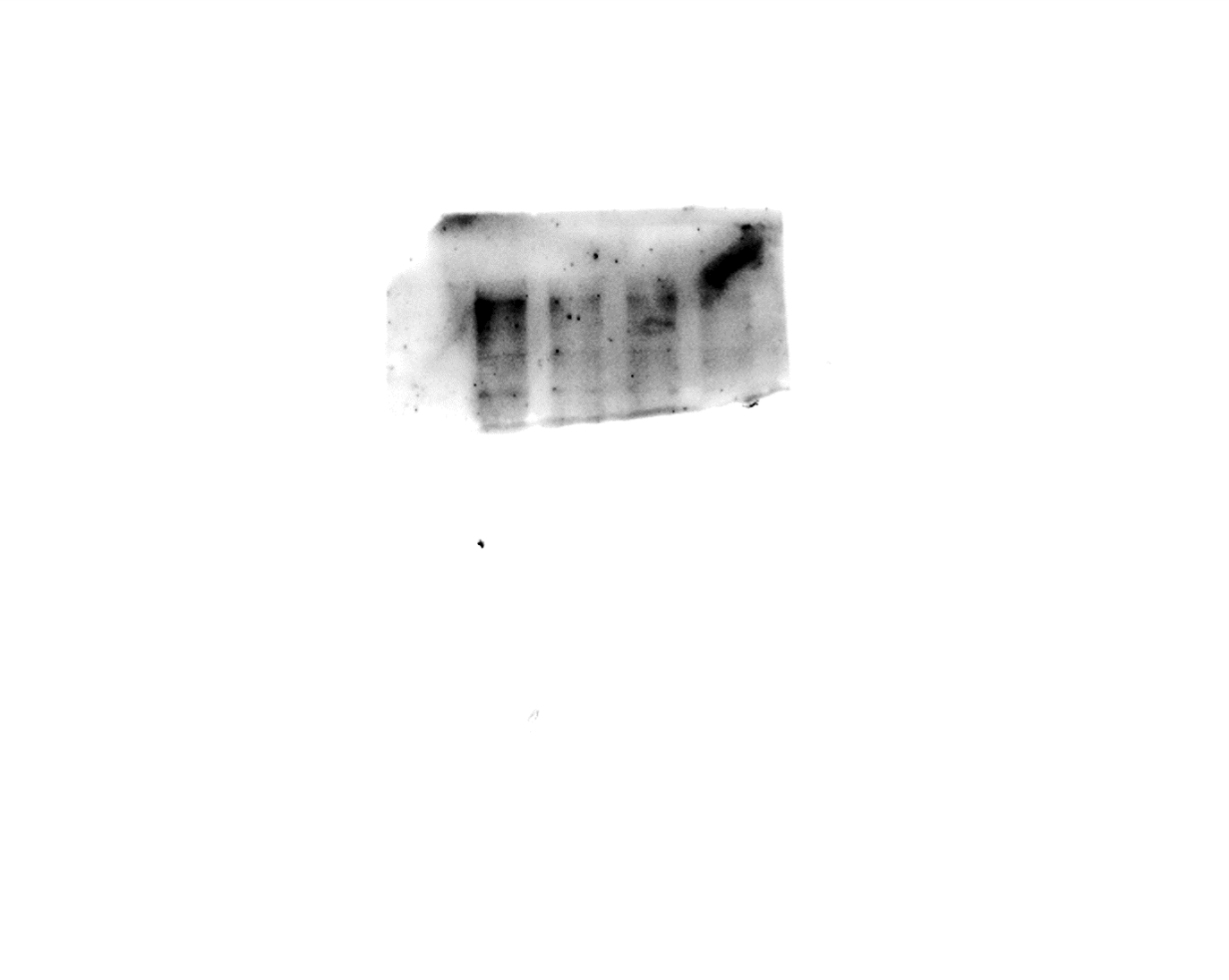


Figure 2F-GAPDH Figure 2F-KITL Figure 2F-mTOR Figure 2F-pmTOR


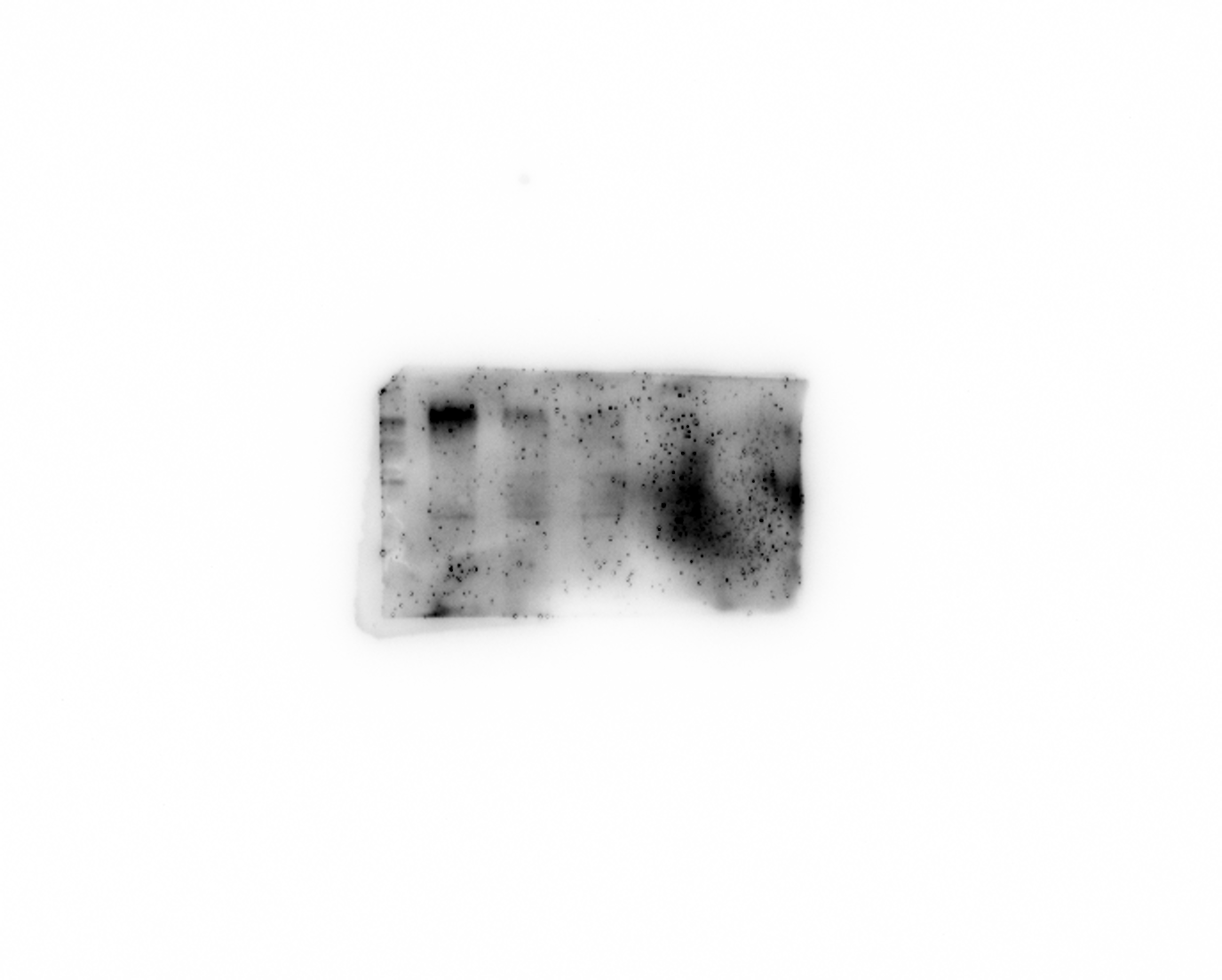

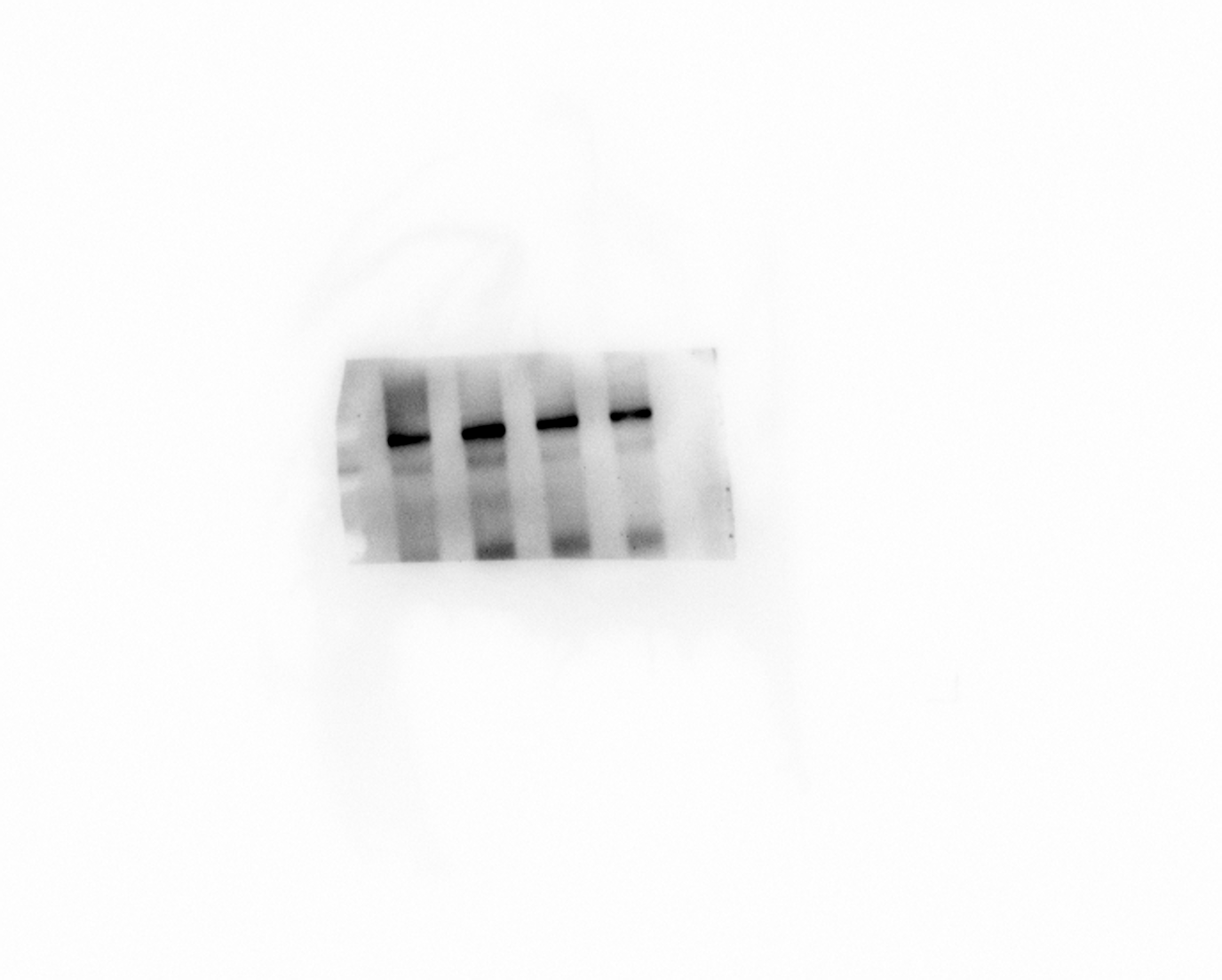


Figure 2F-PAR Figure 2F-PARP1


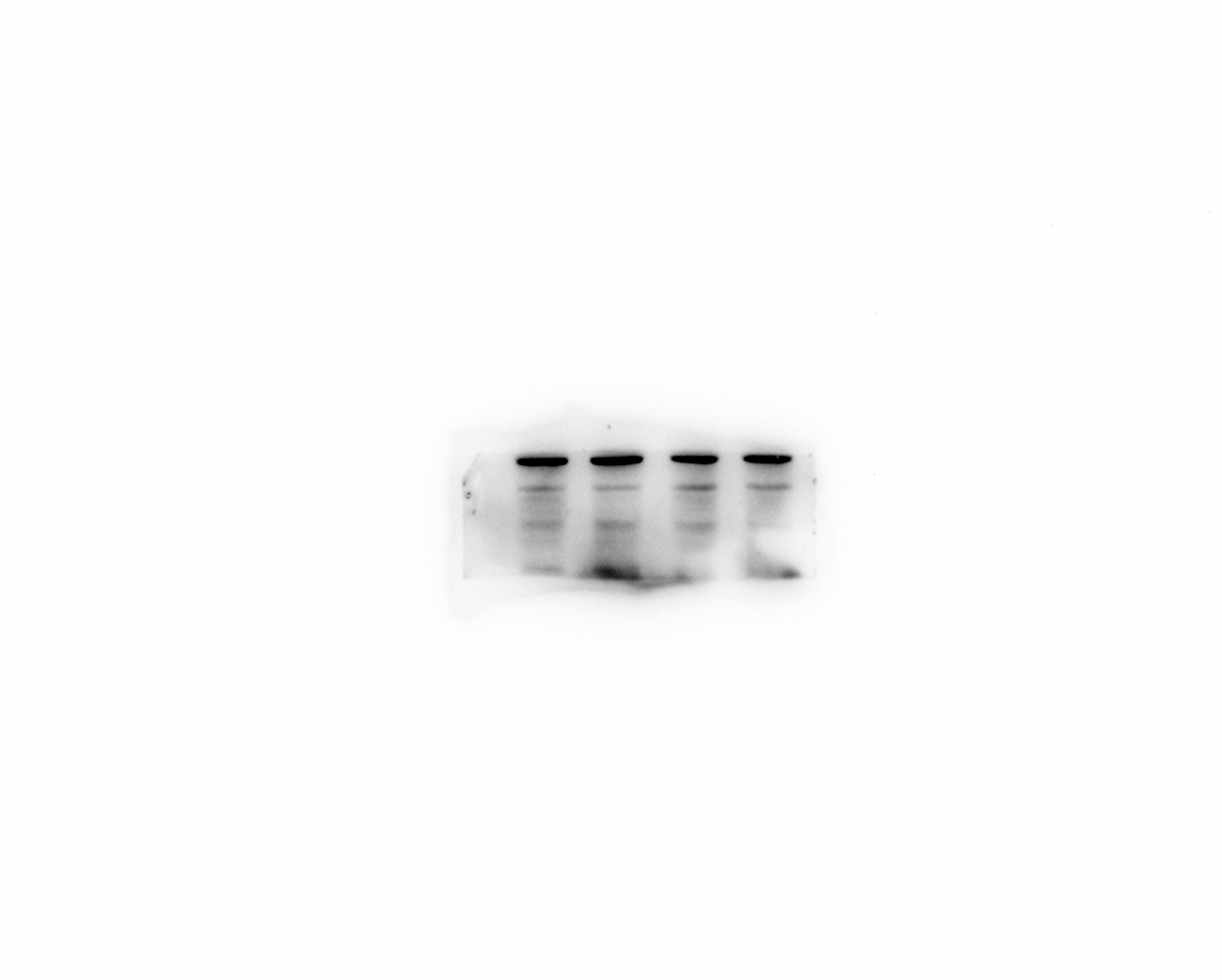

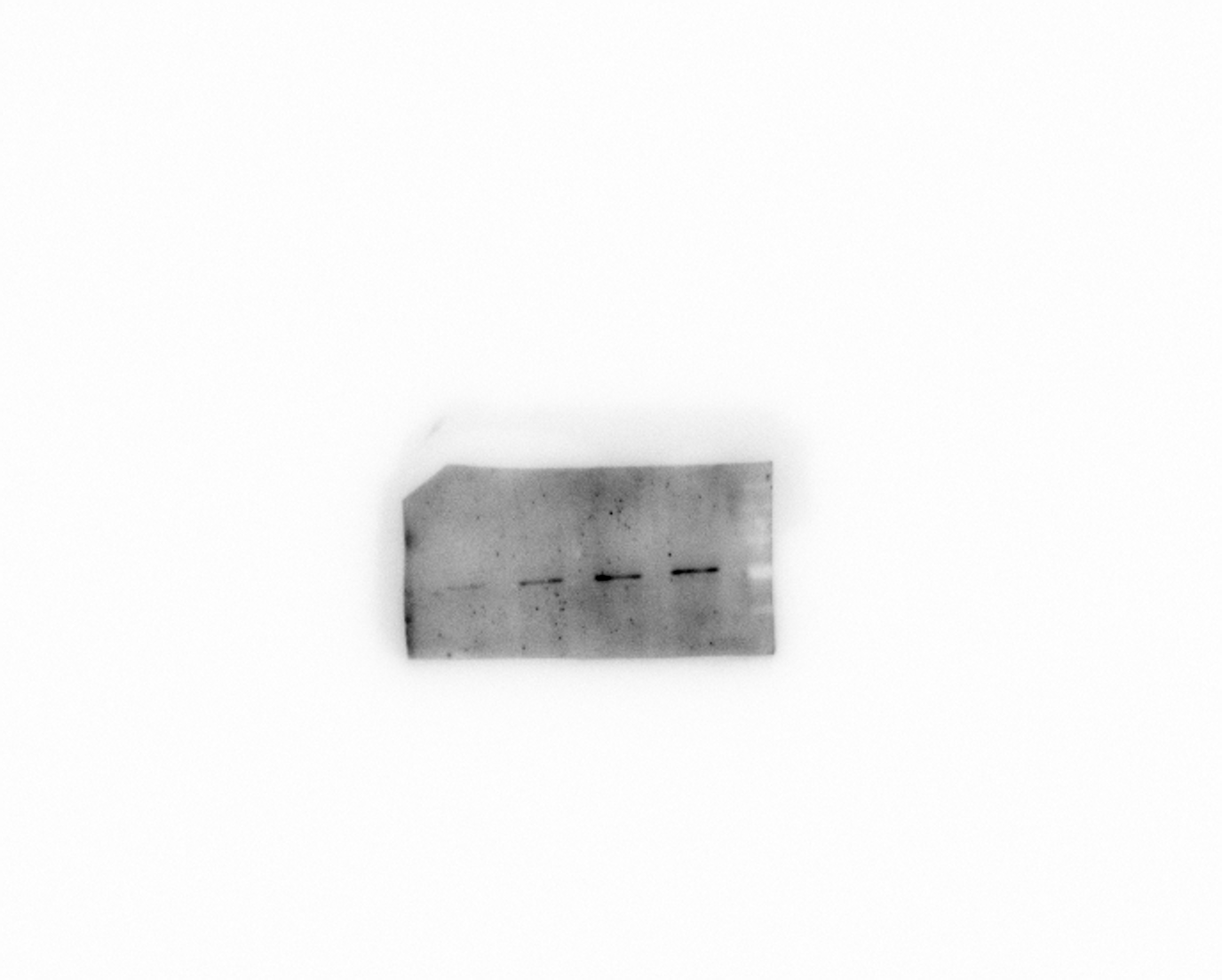

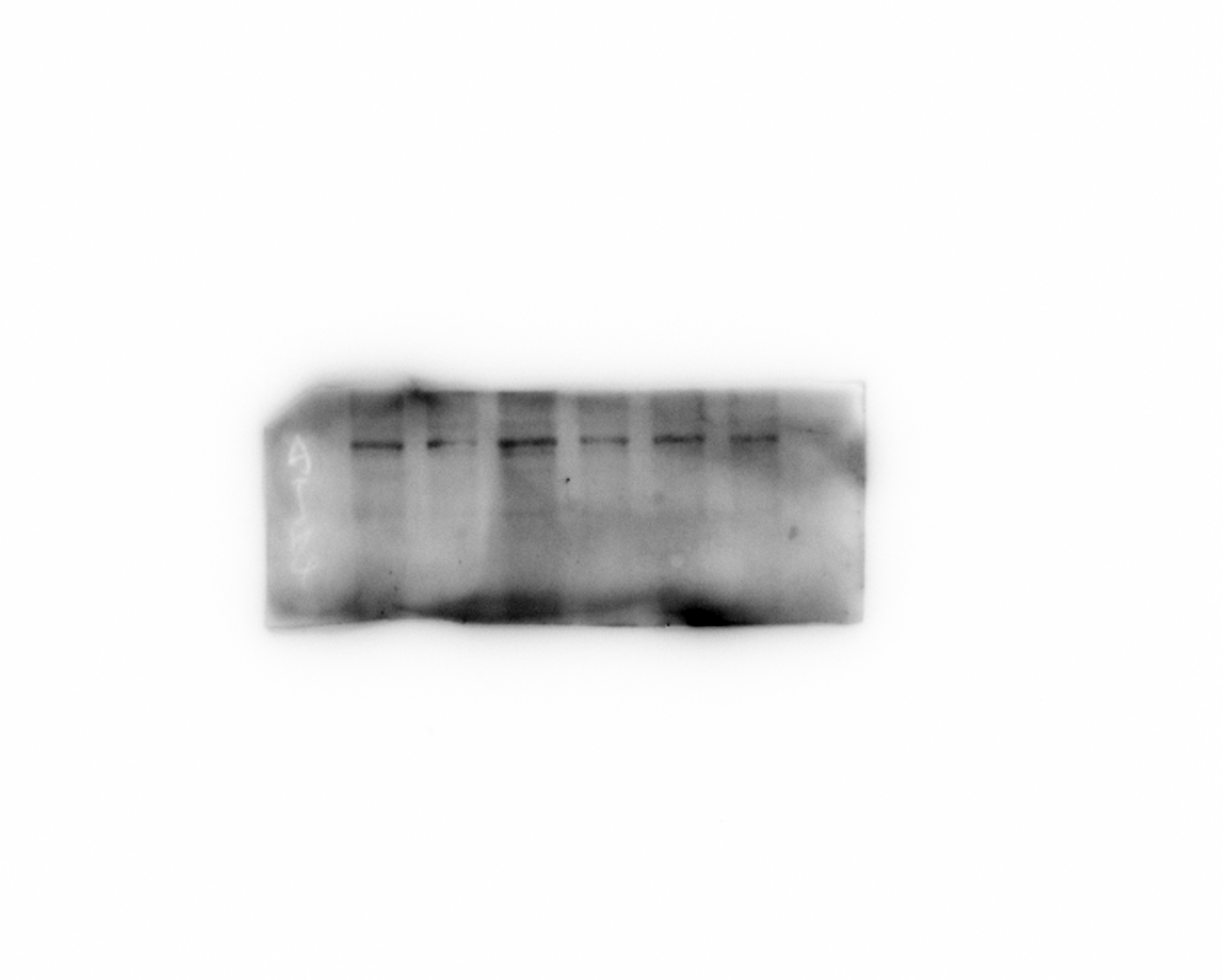

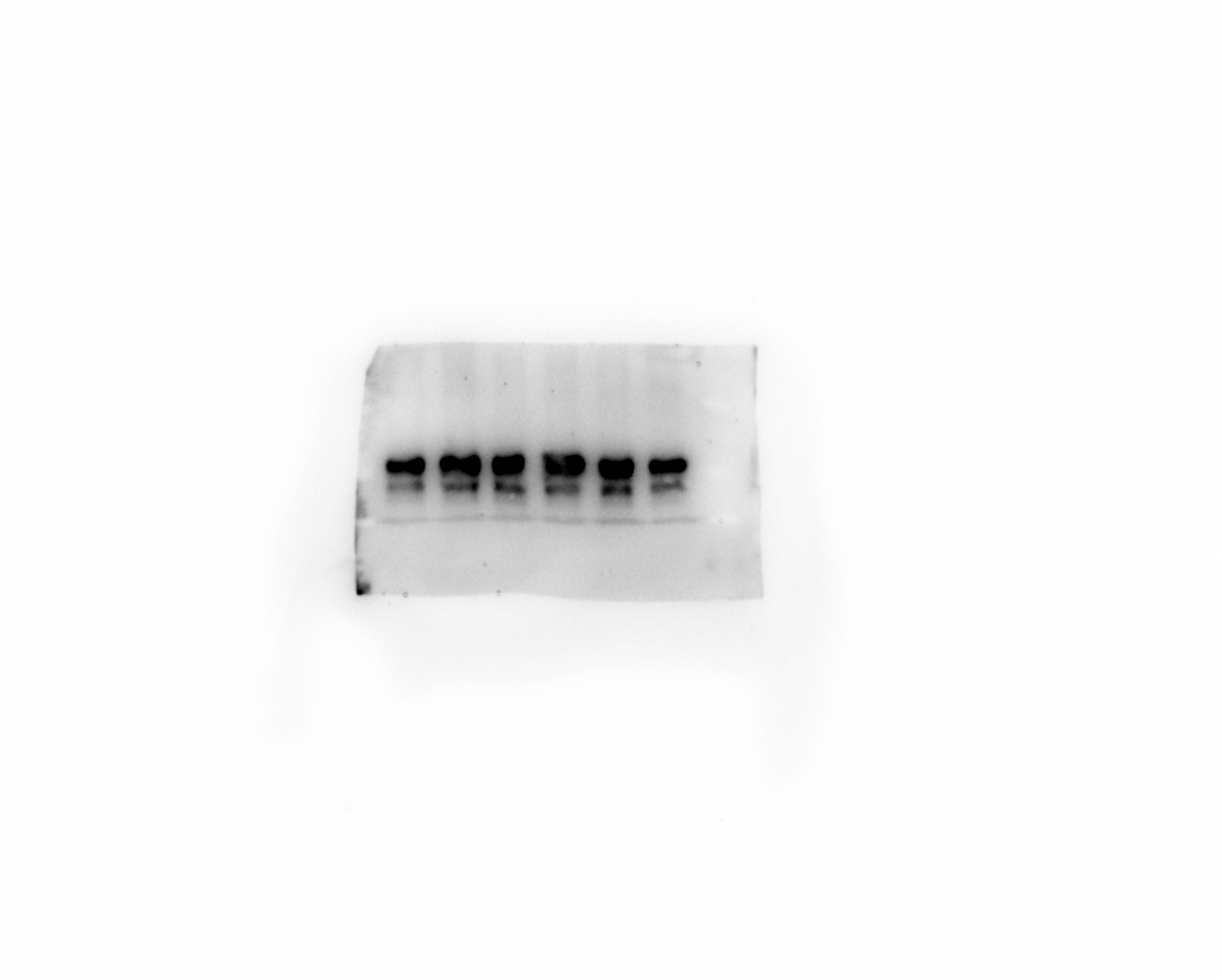


Figure 3B-GAPDH Figure 3B-GRP78 Figure 3D-ATF4 Figure 3D-GAPDH


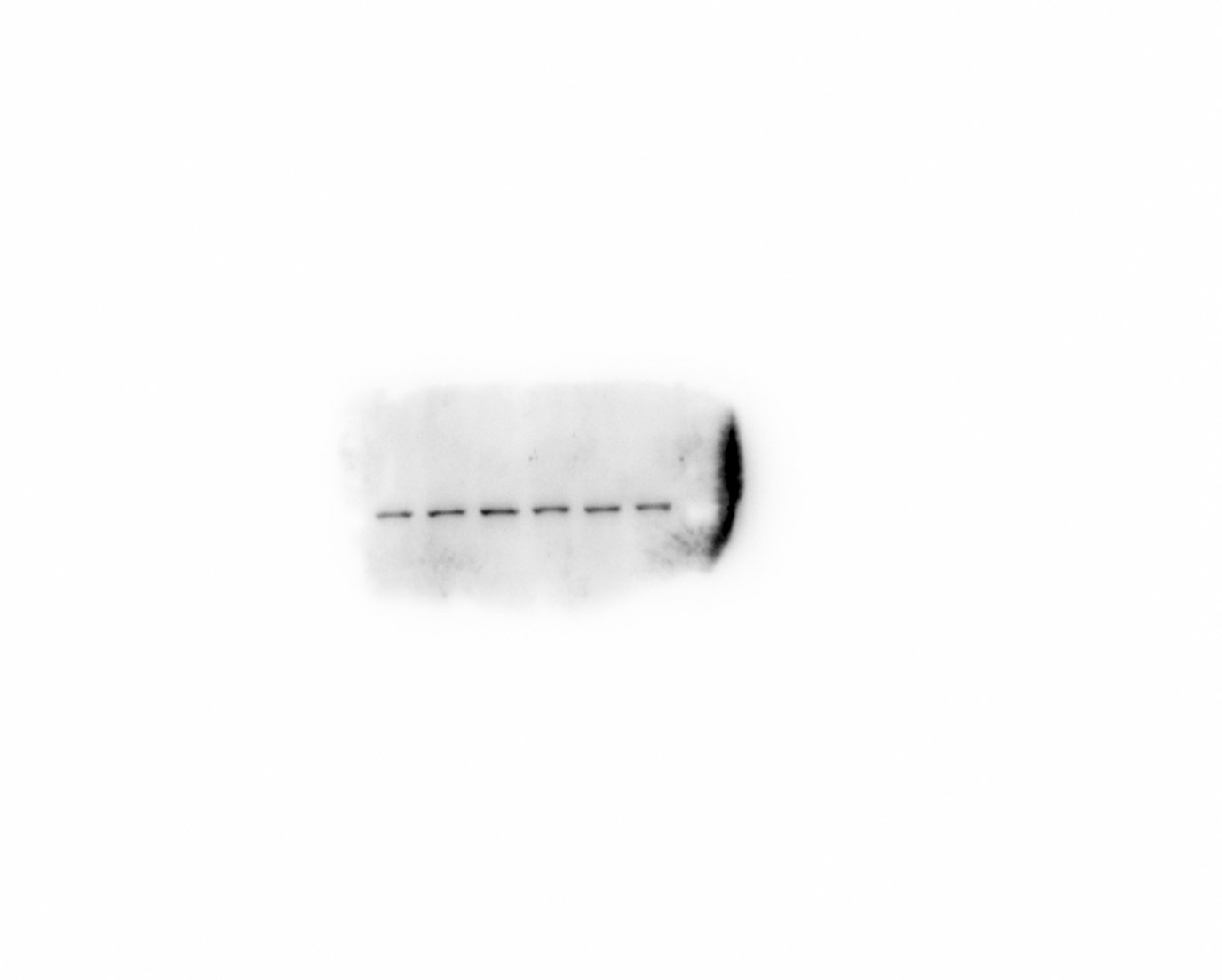

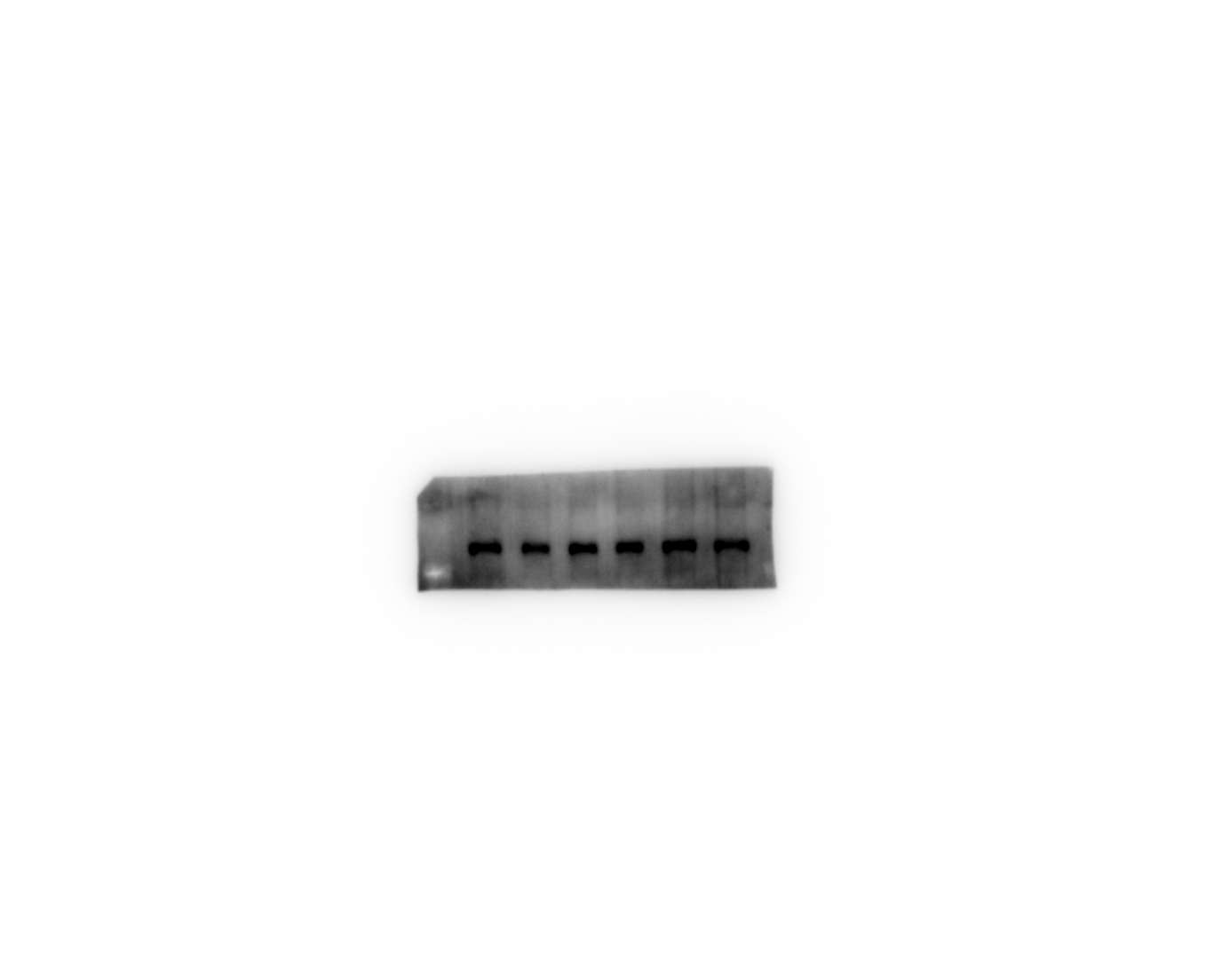

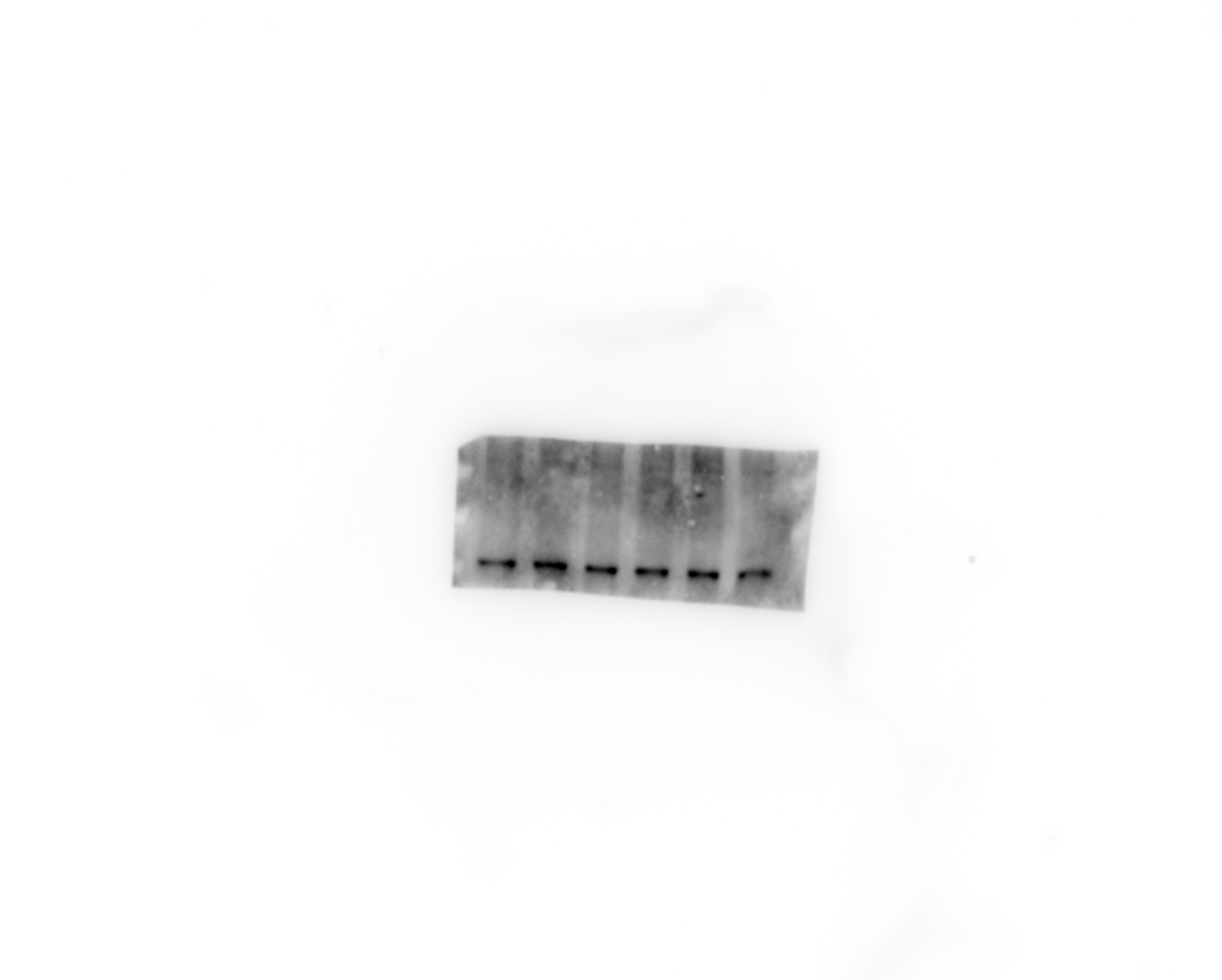


Figure 3D-GRP78 Figure 3D-IRE1α Figure 3D-PERK


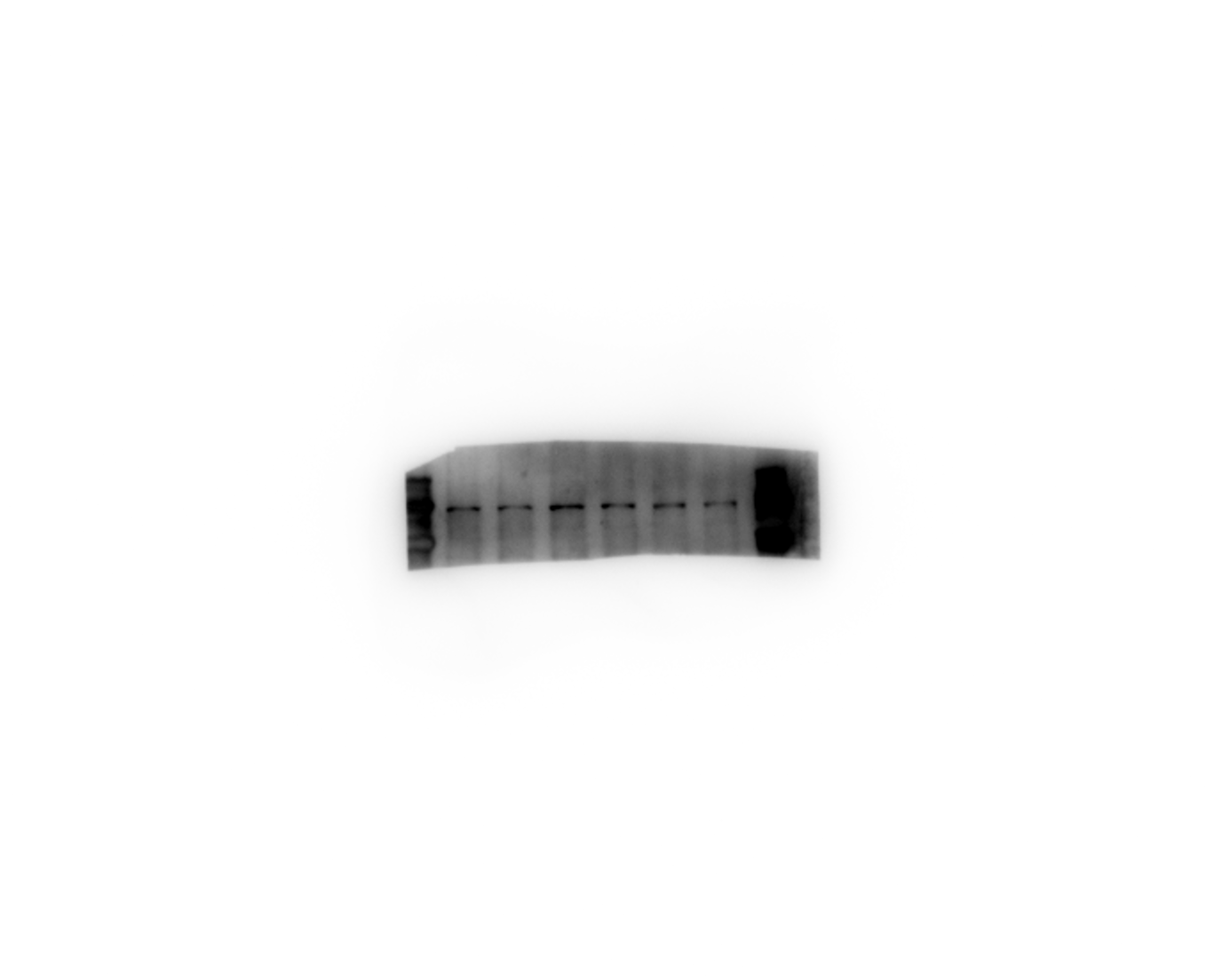

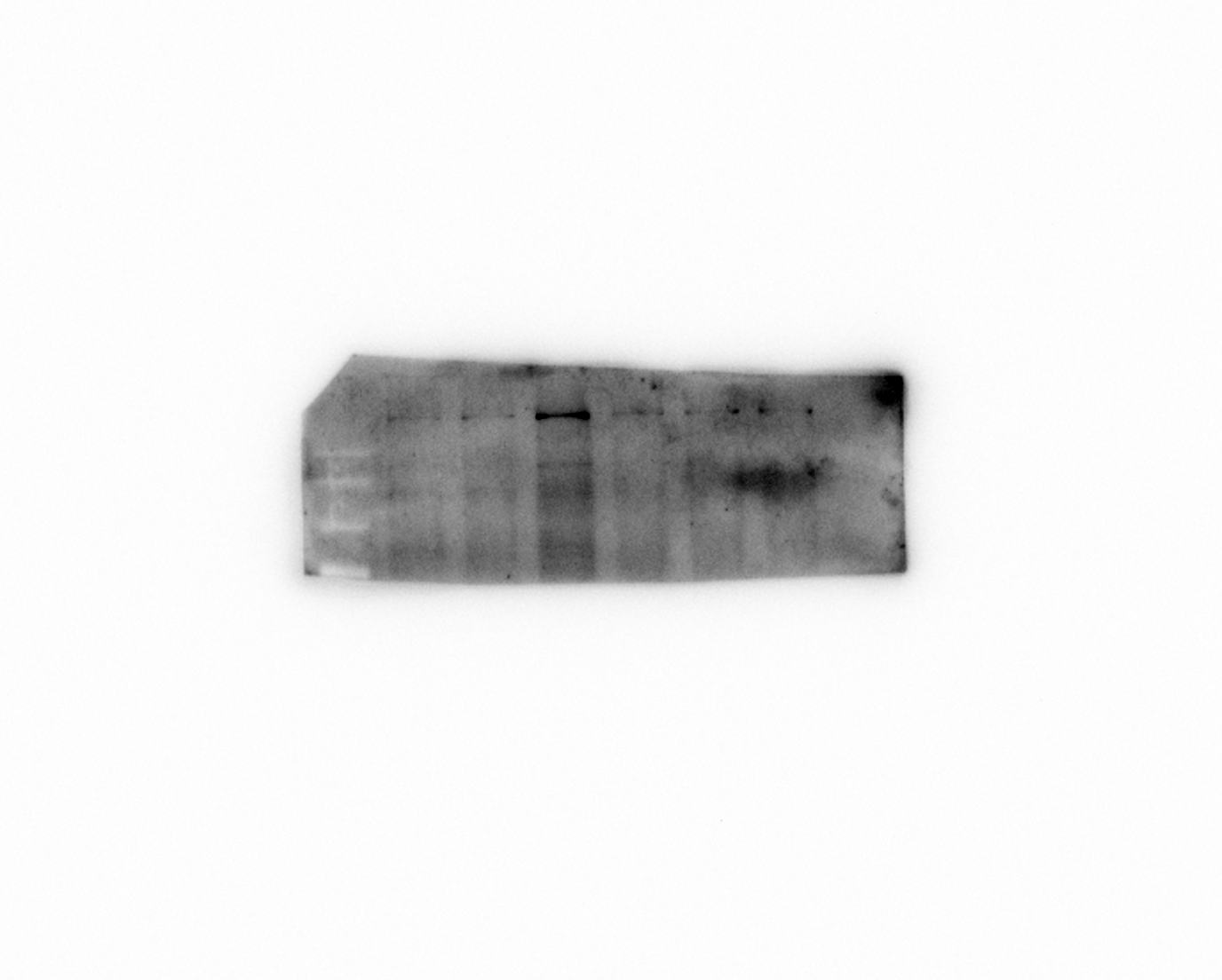


Figure 3D-pIRE1α Figure 3D-pPERK


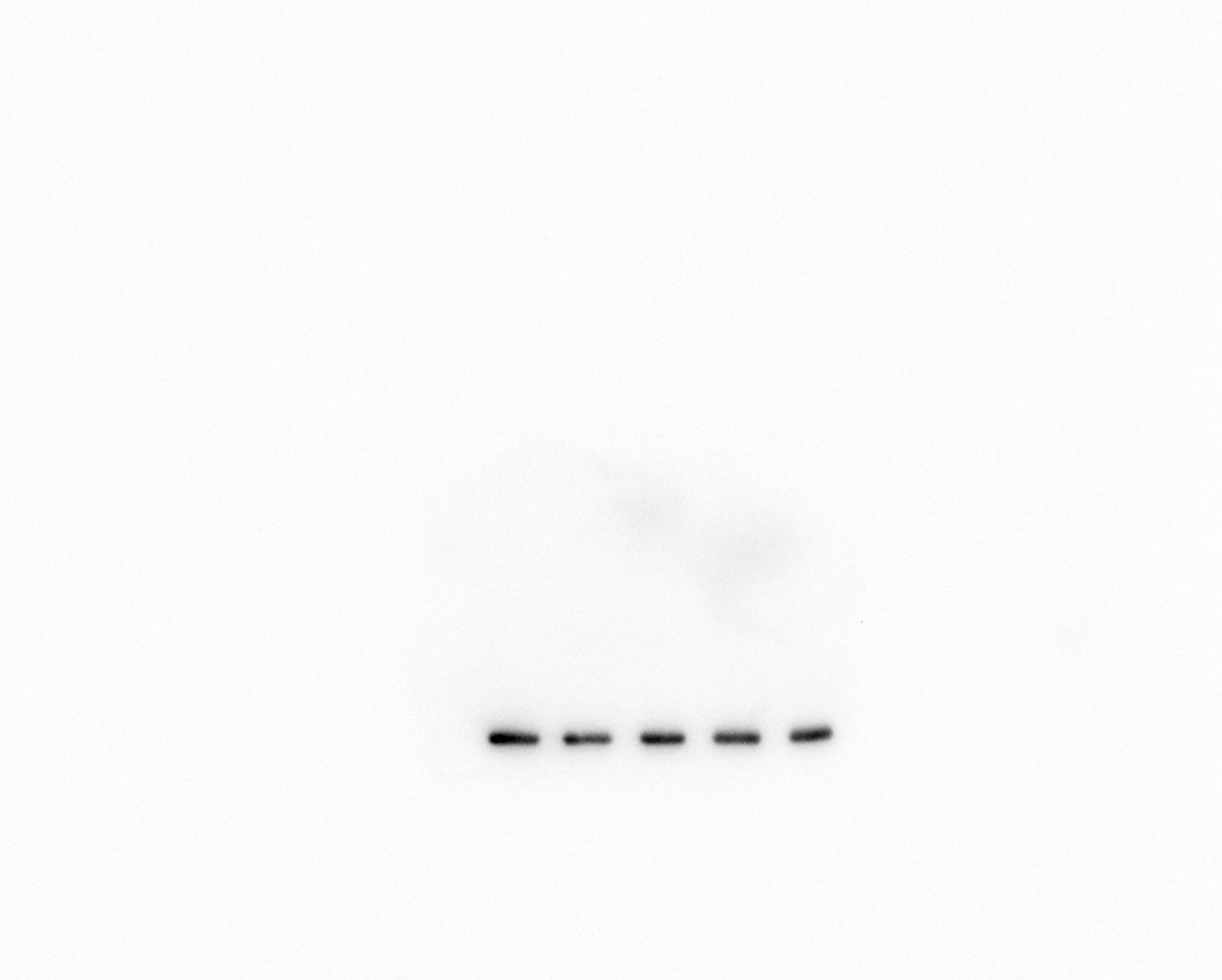

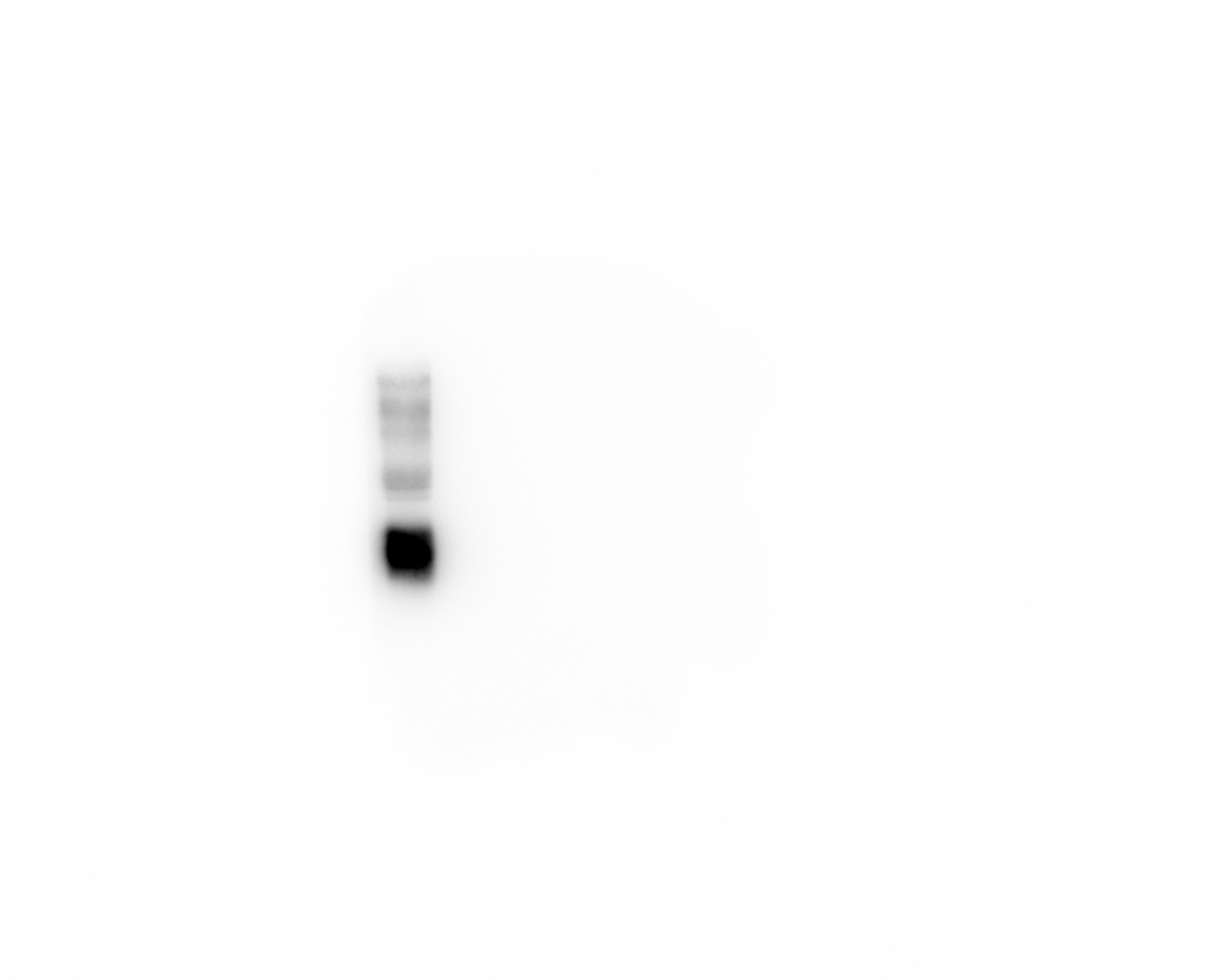

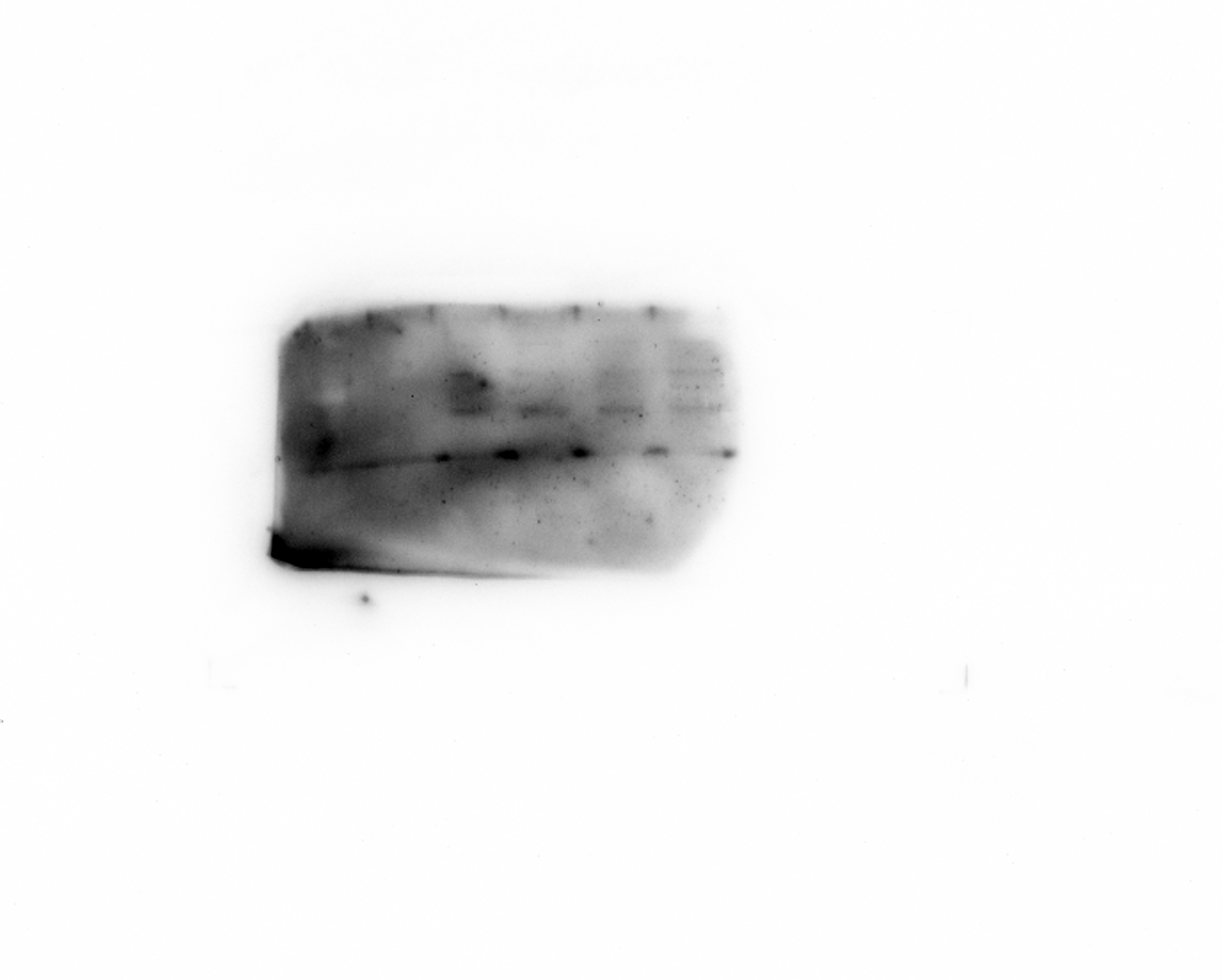

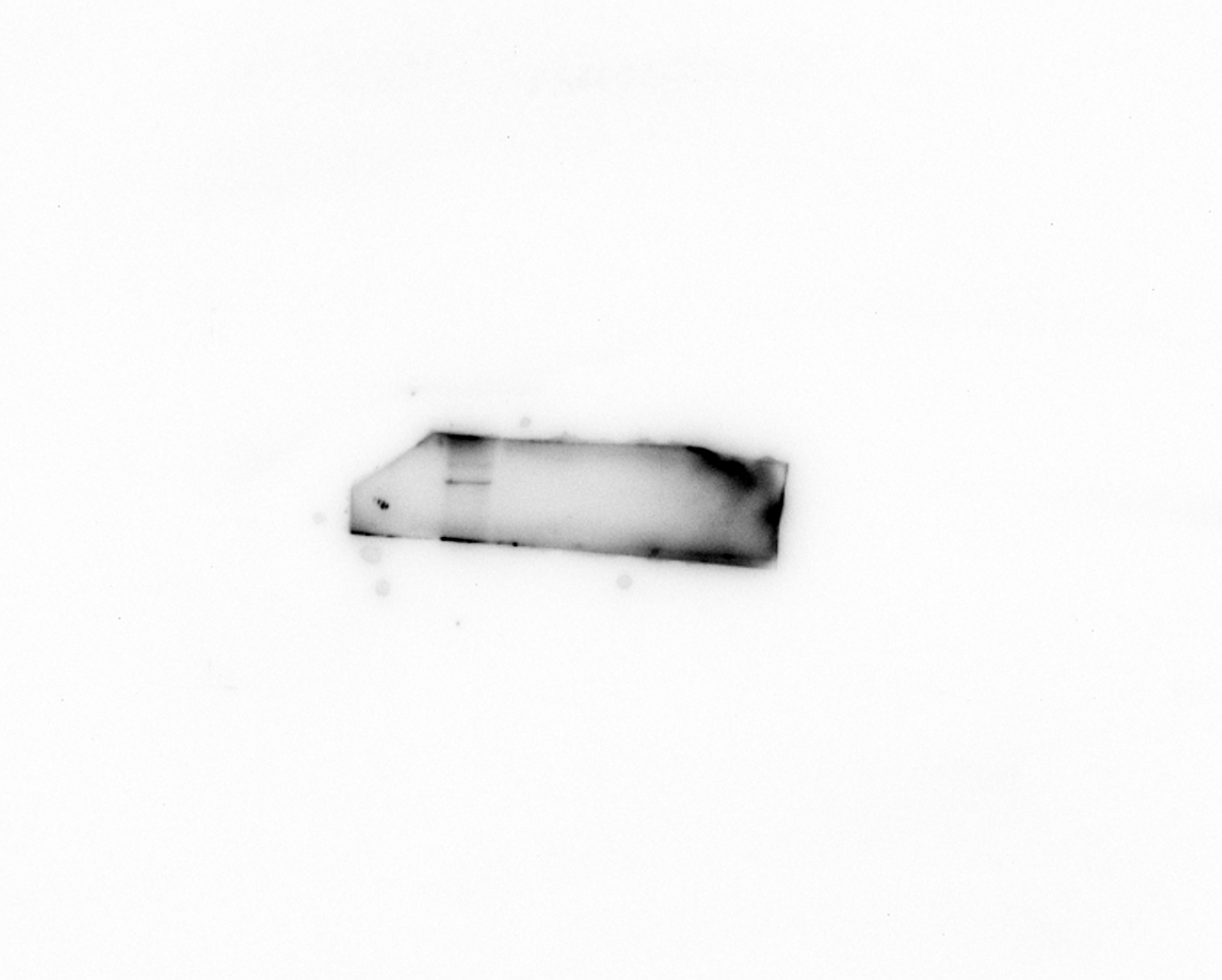


Figure 4A-GAPDH Figure 4A-GRP78 PARylation Figure 4A-GRP78 Figure 4A-YY1


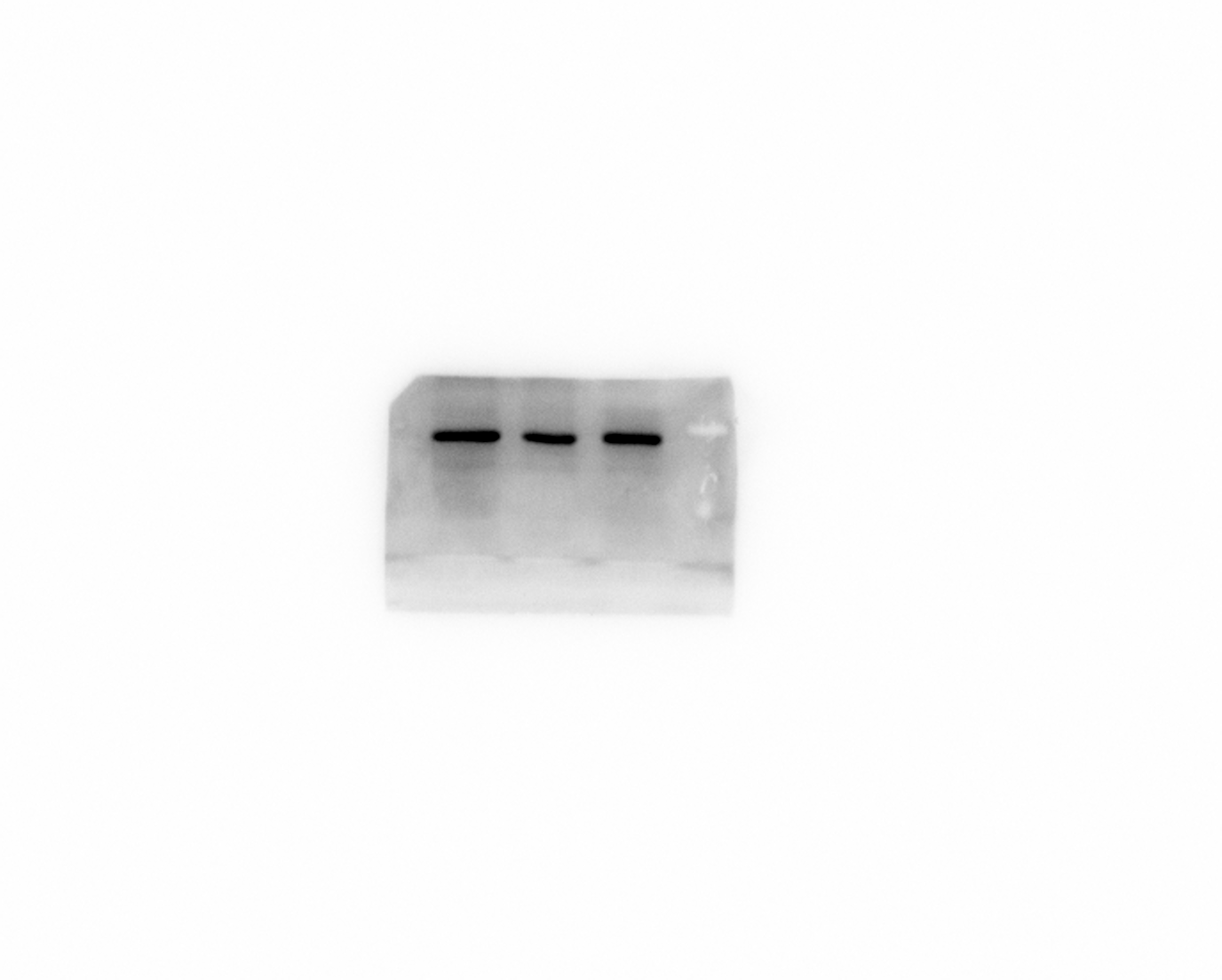

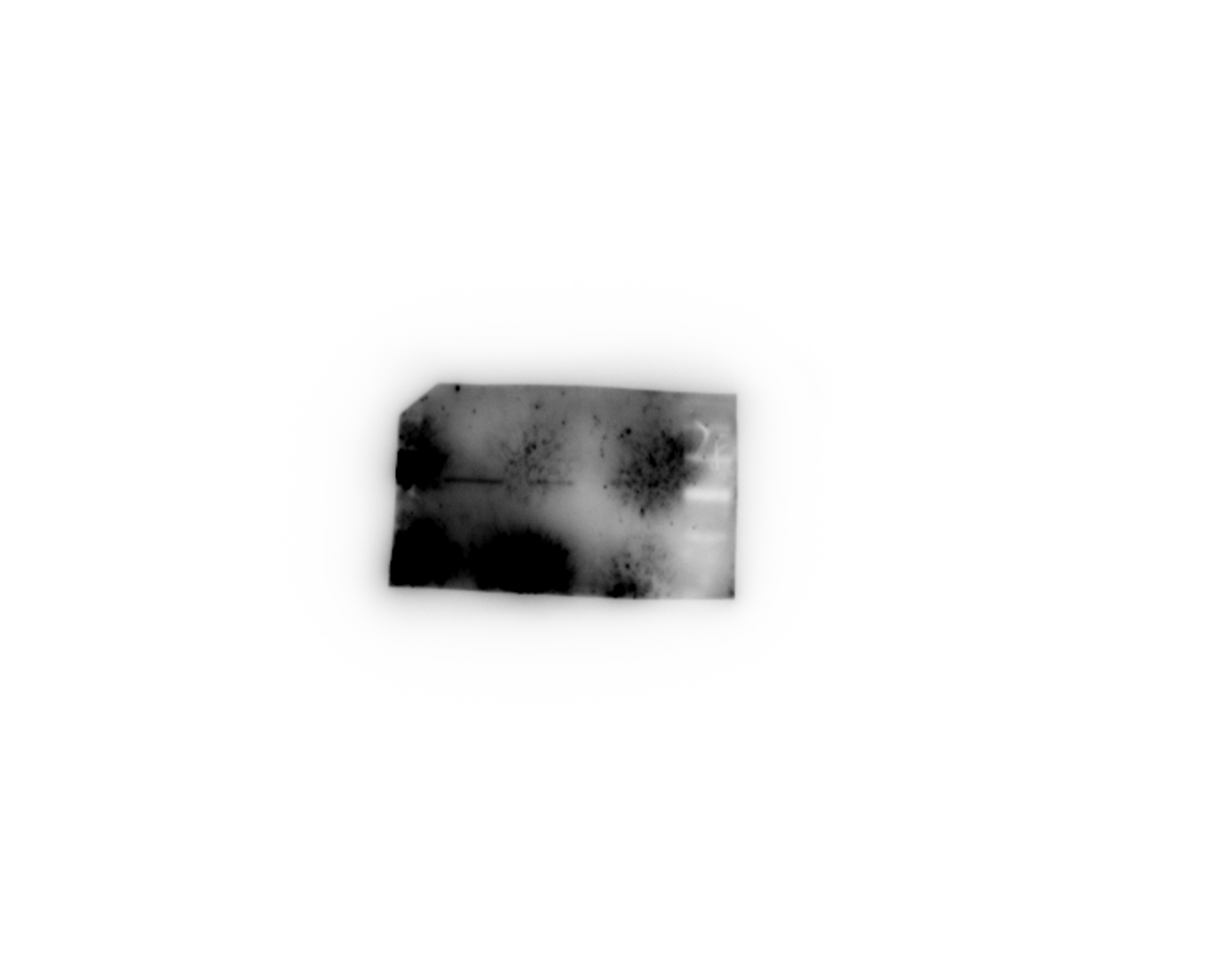

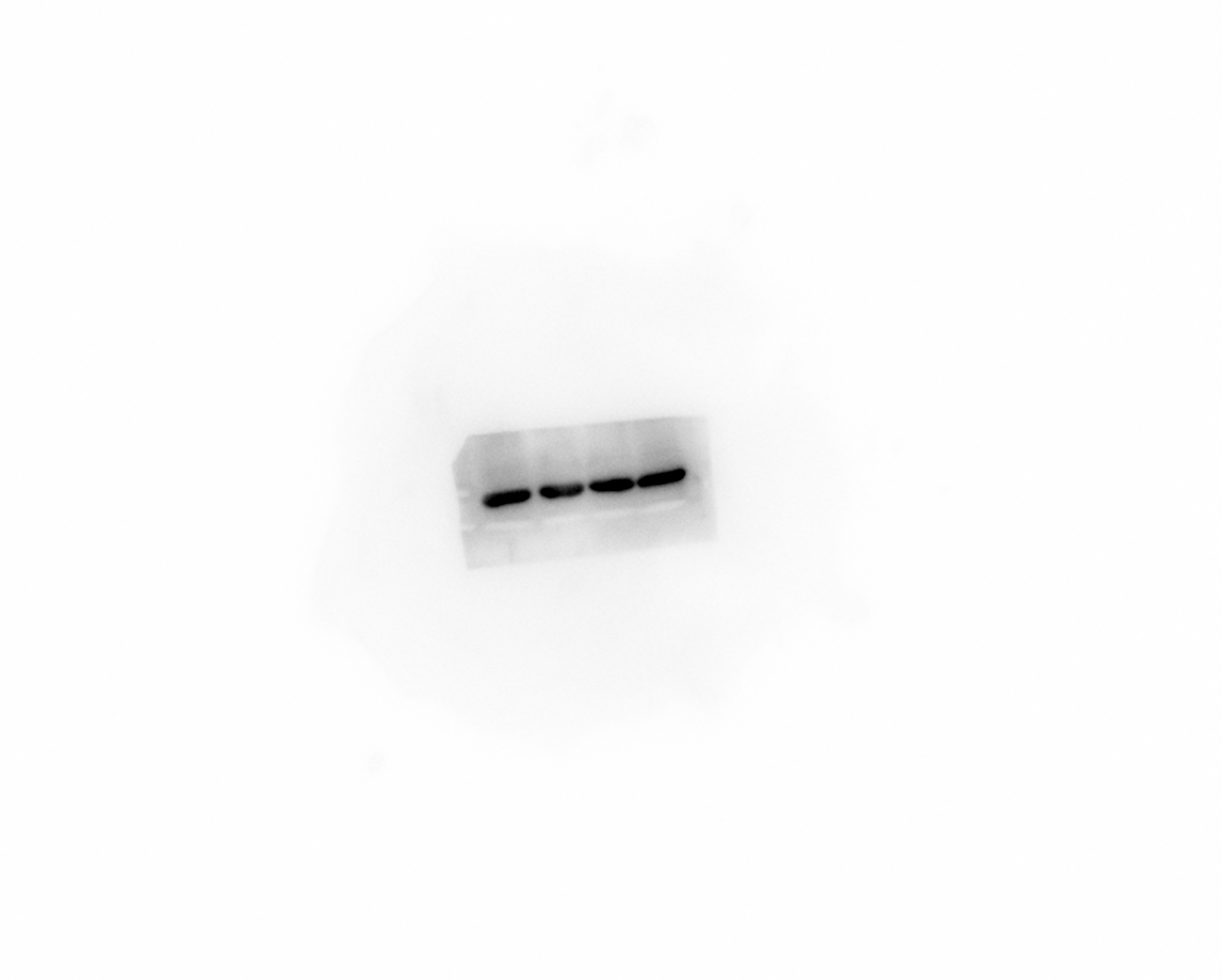

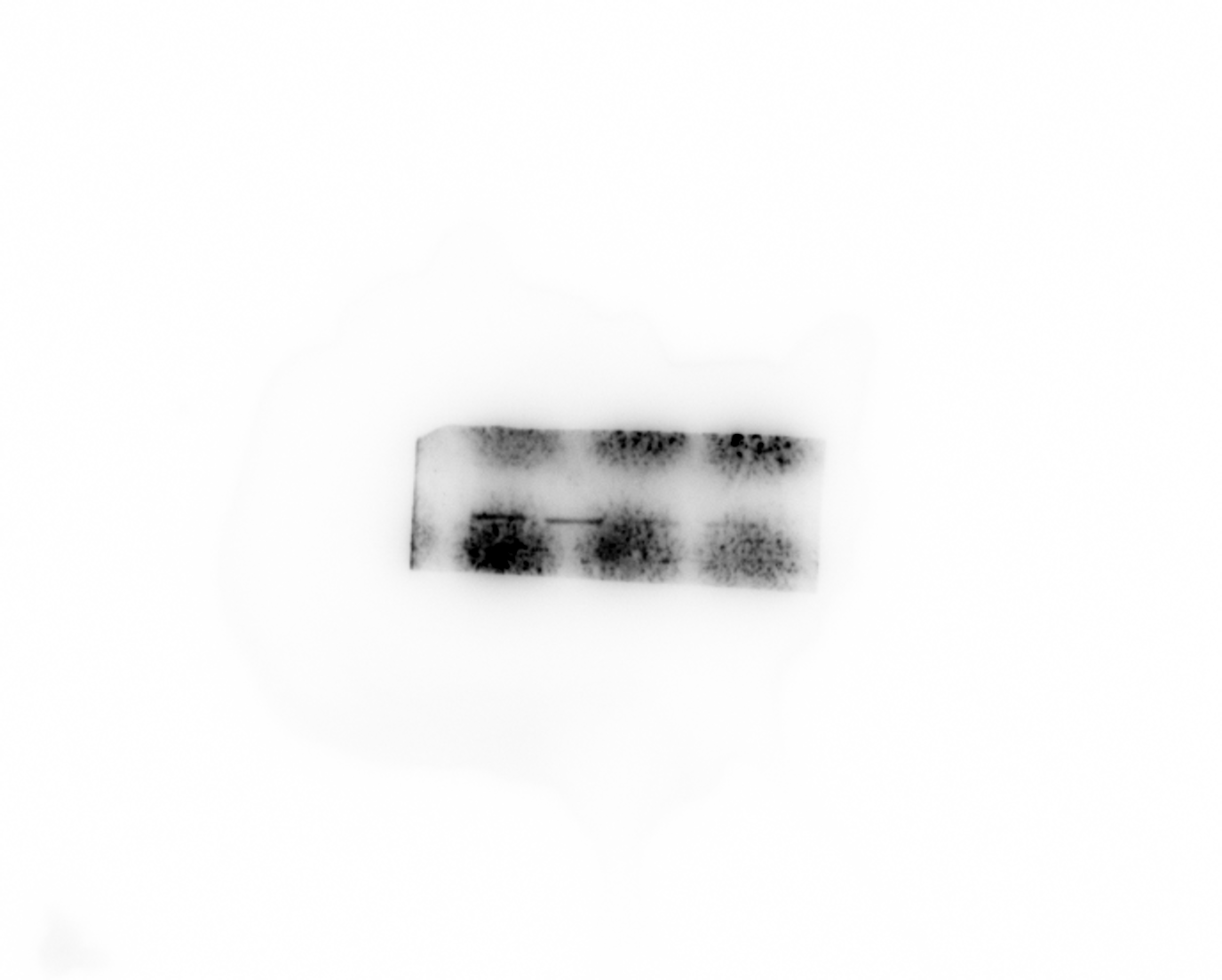


Figure 4B-GAPDH Figure 4B-GRP78 Figure 4C-GAPDH Figure 4C-GRP78


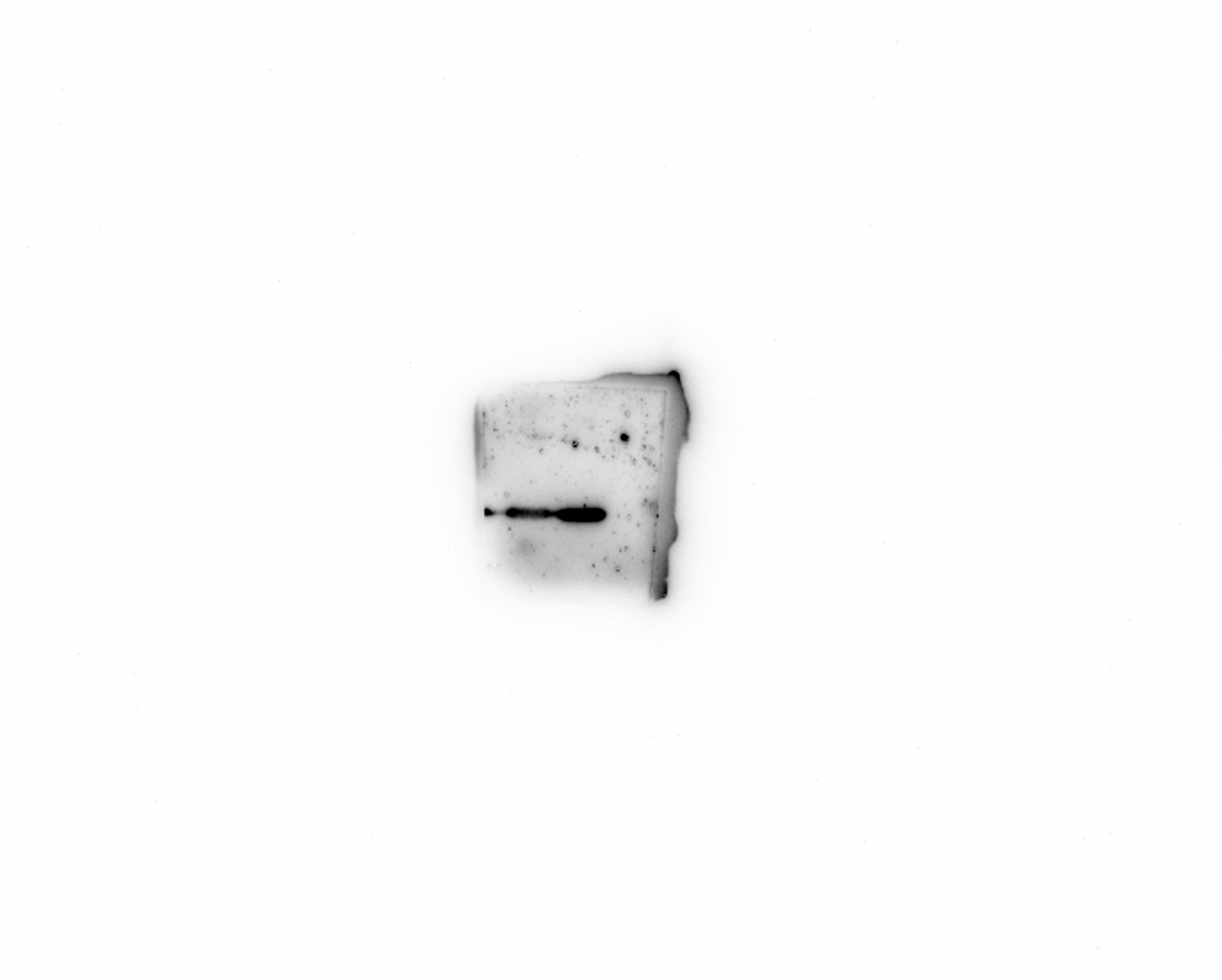

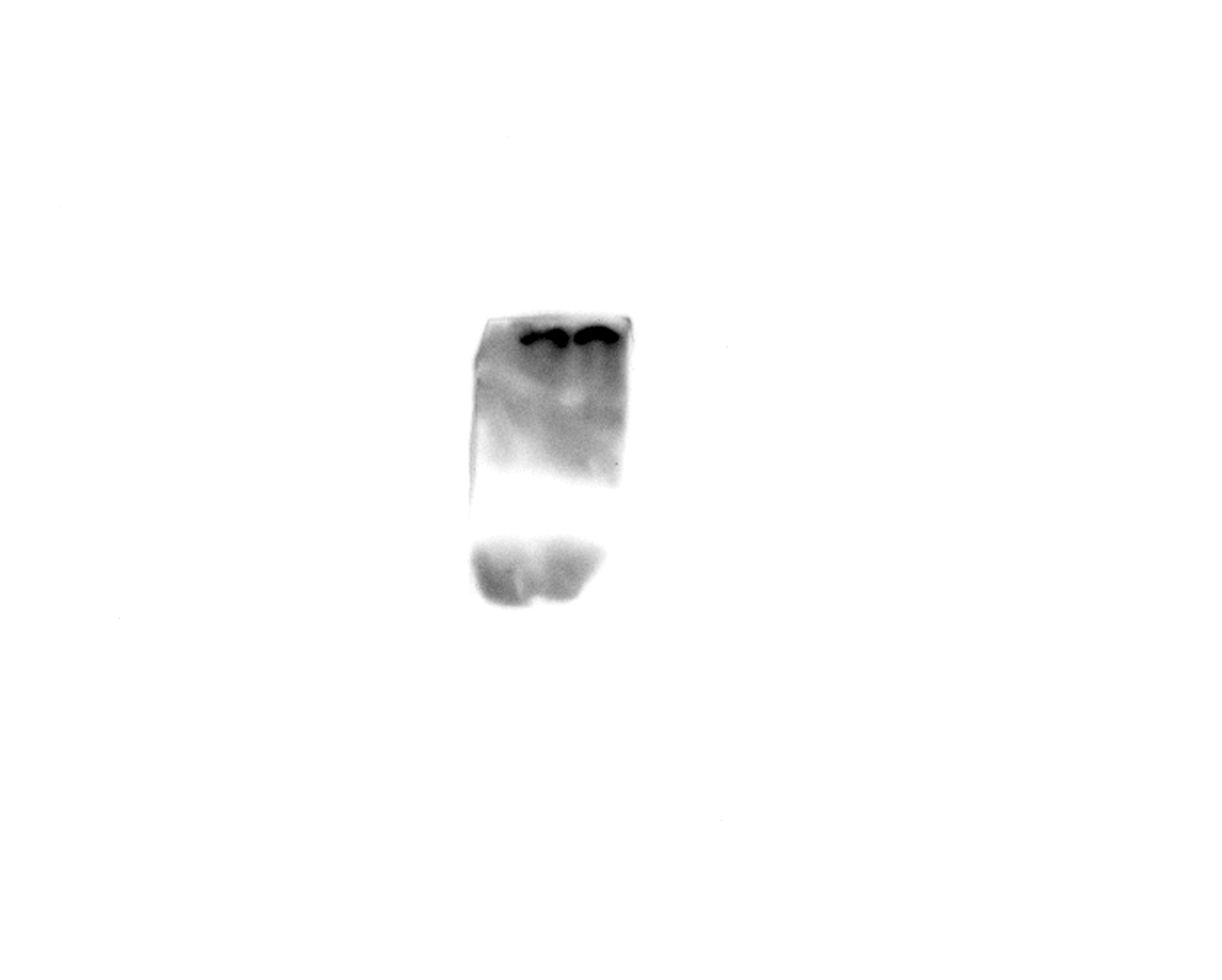

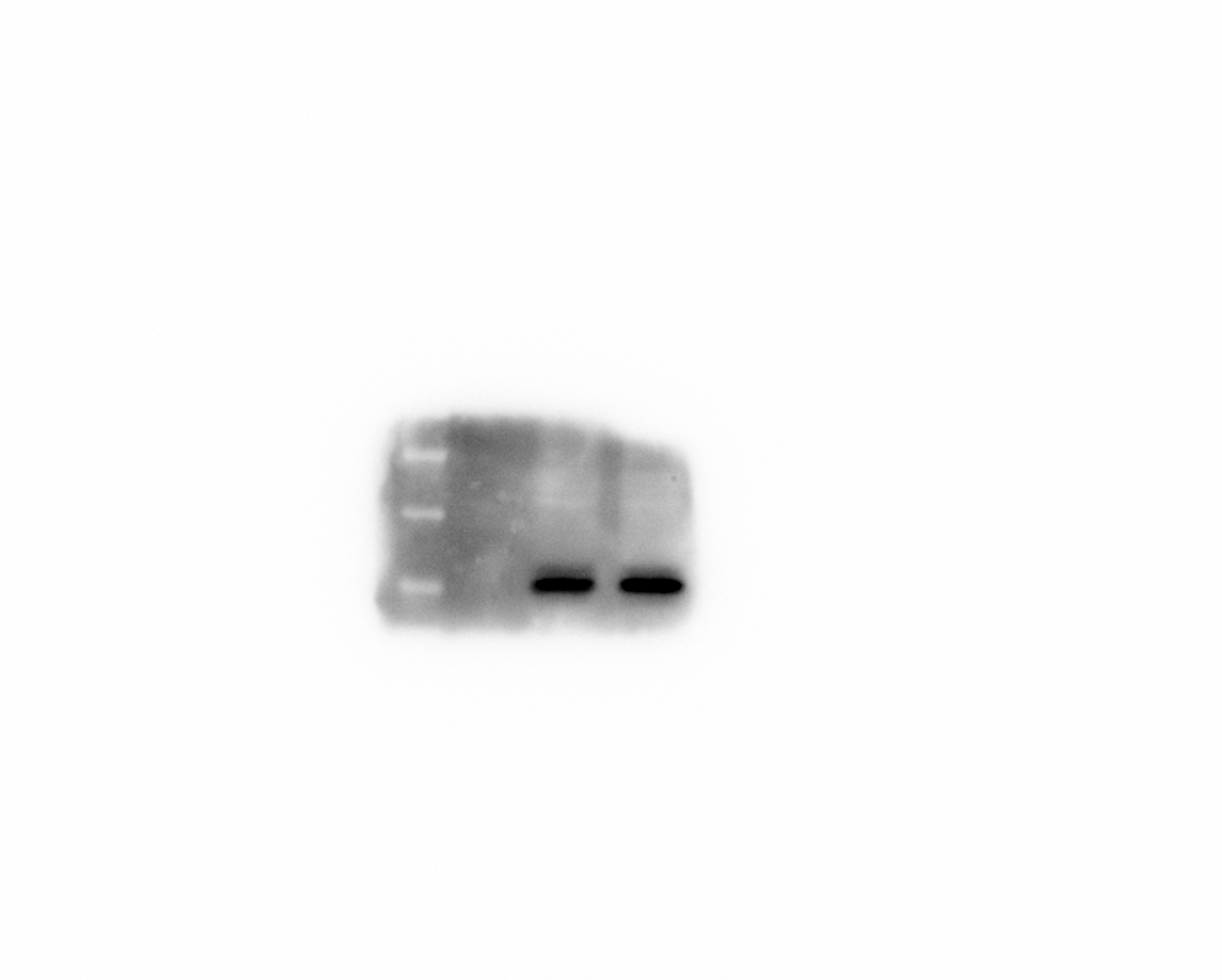

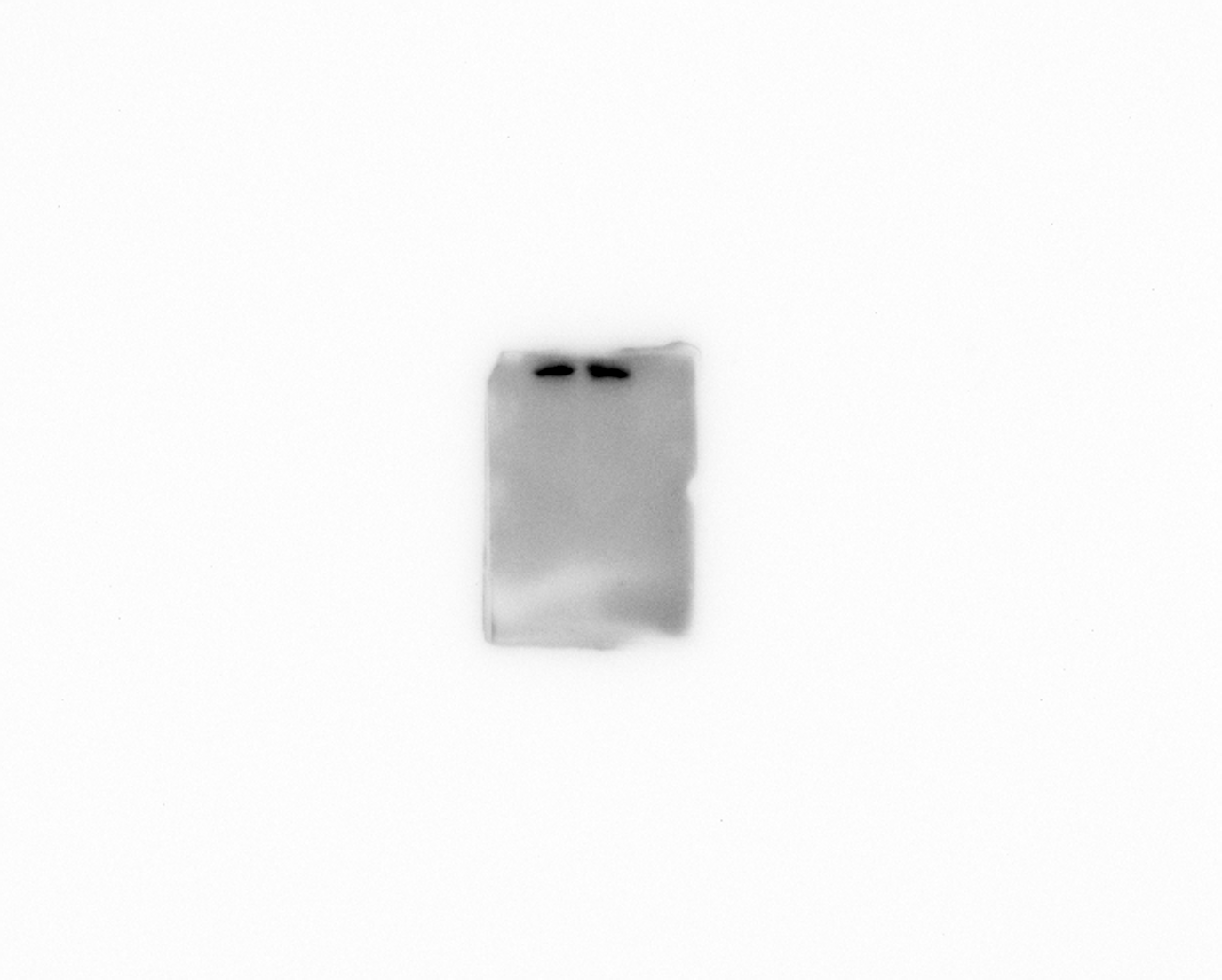

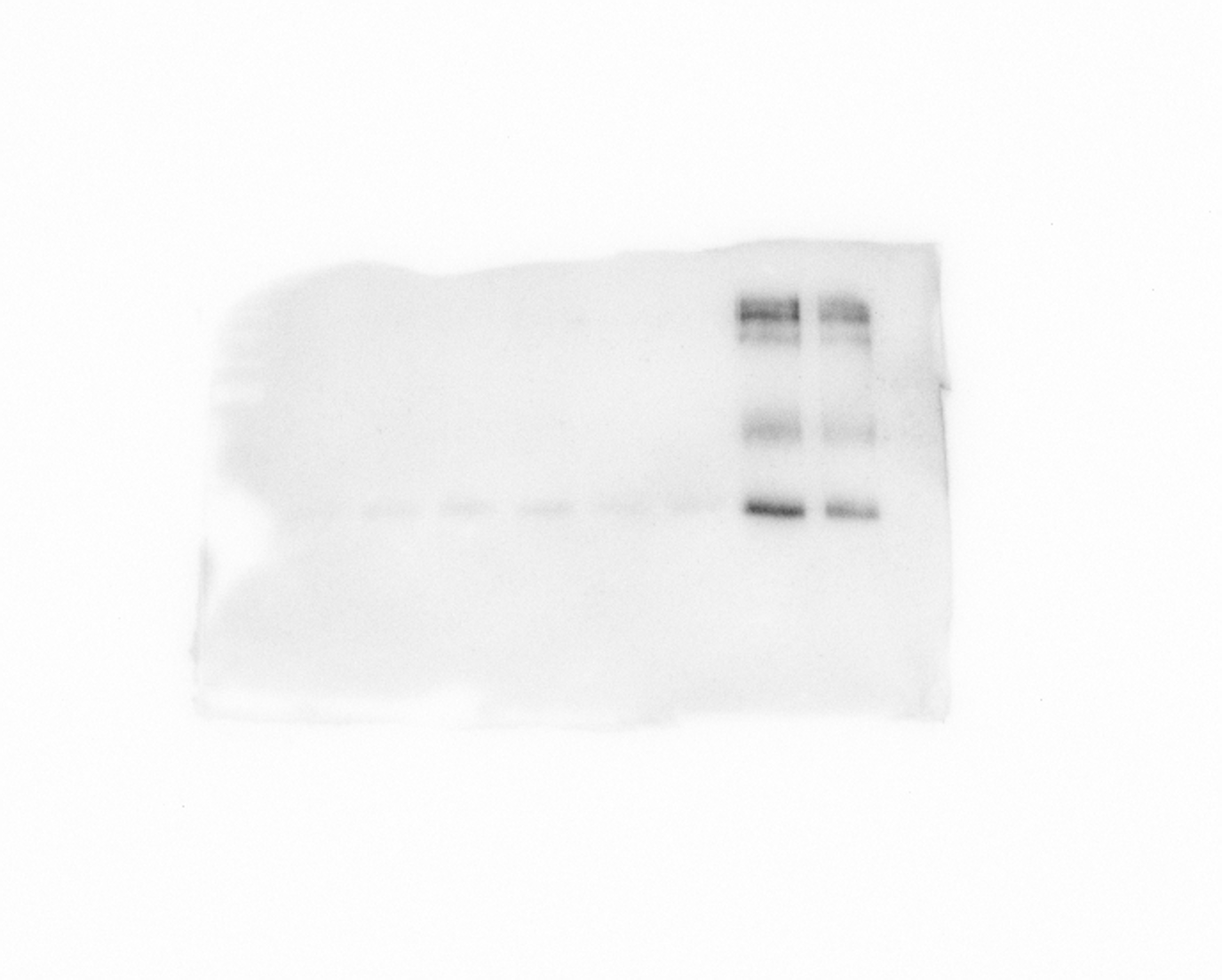


Figure 5B-GAPDH Figure 5B-YY1 Figure 5C-GAPDH Figure 5C-YY1.Tif Figure 5D-FLAG PARylation


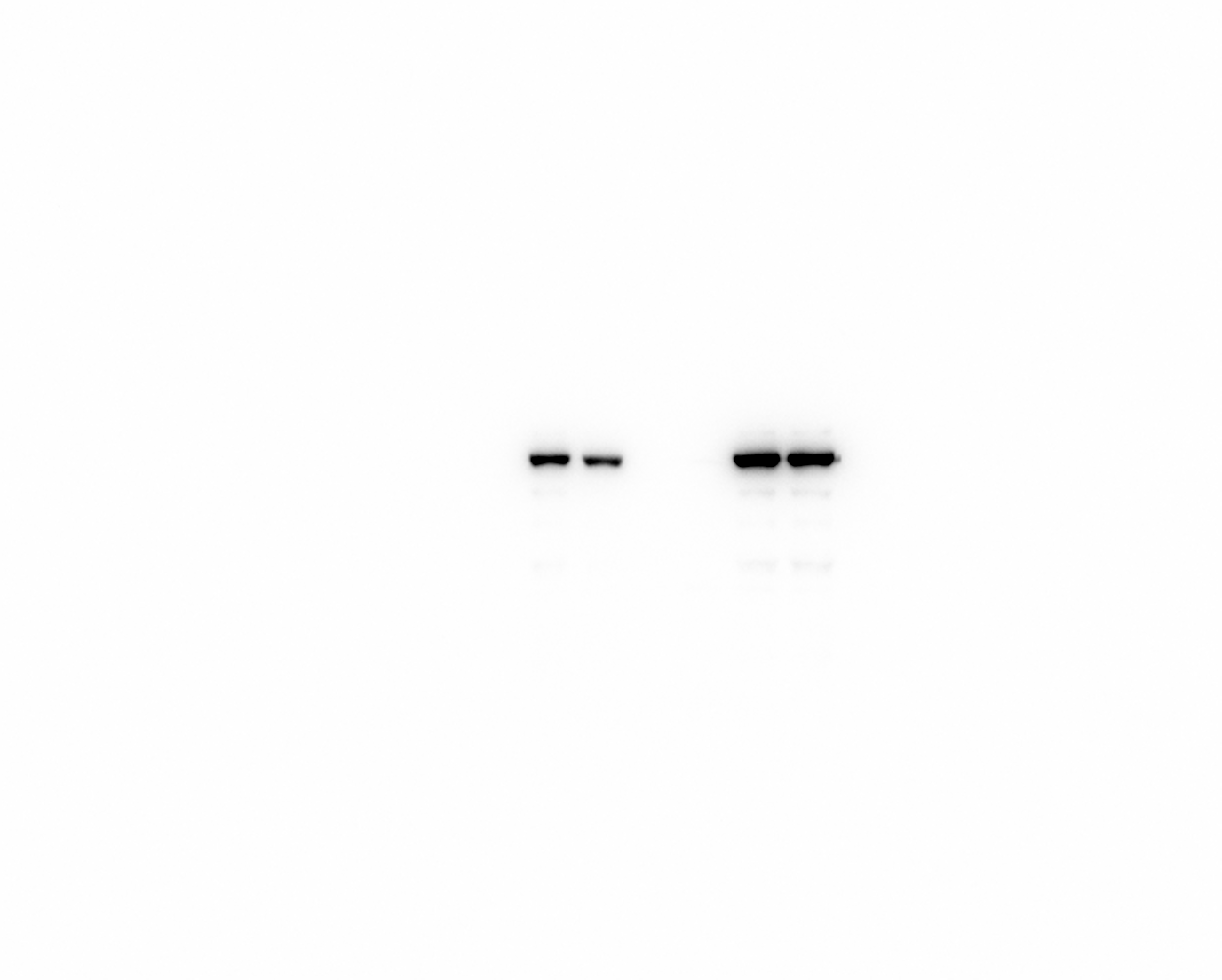

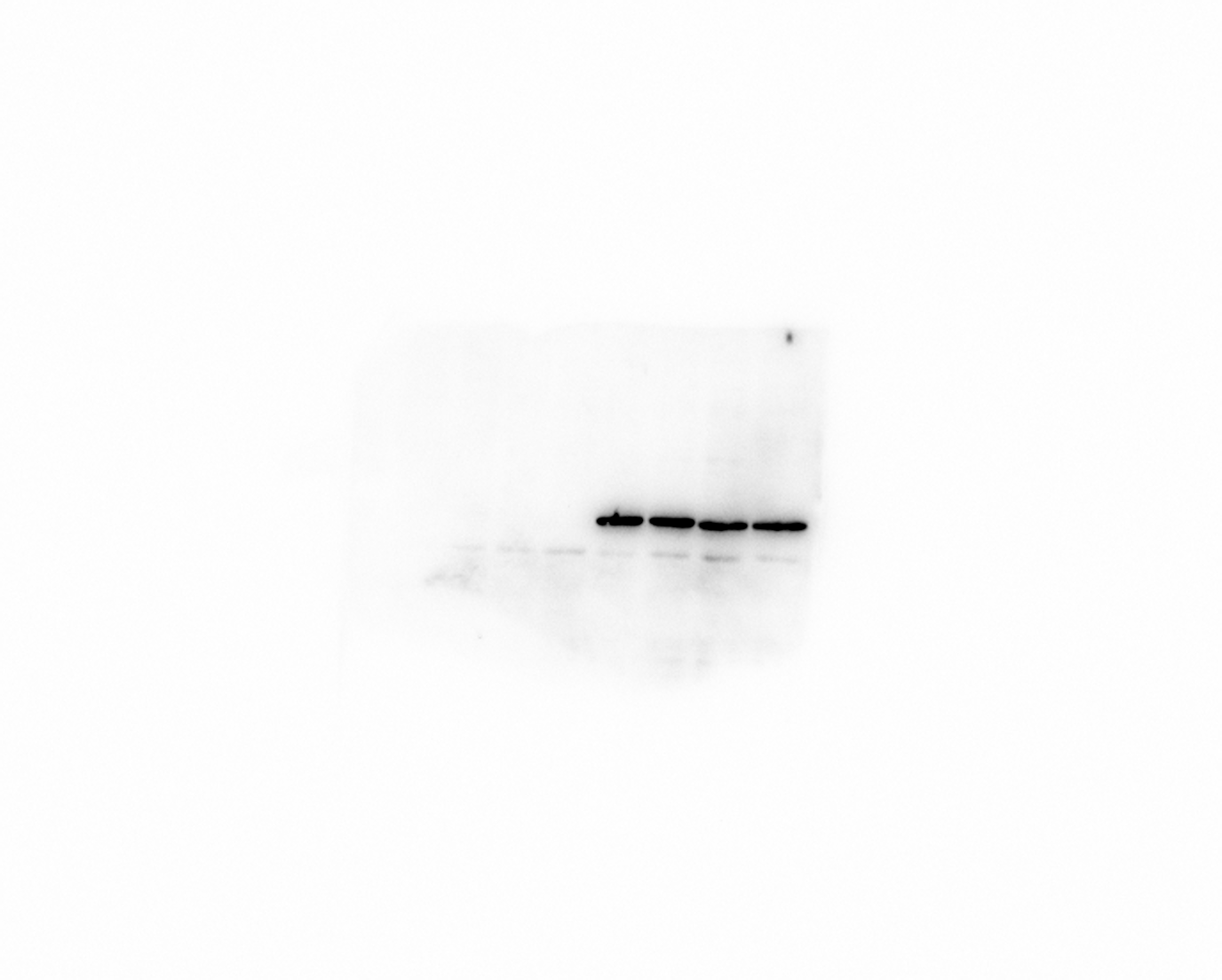

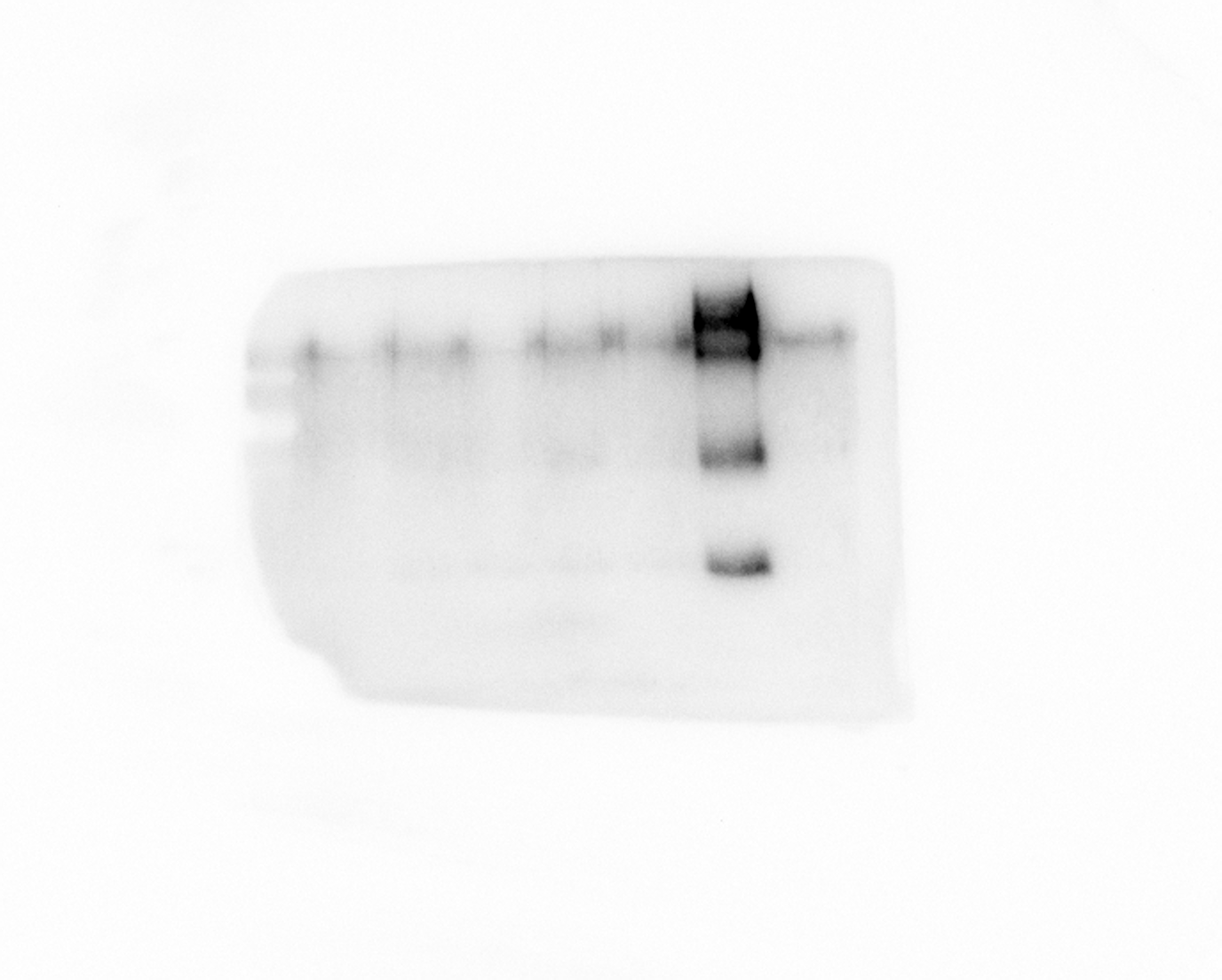

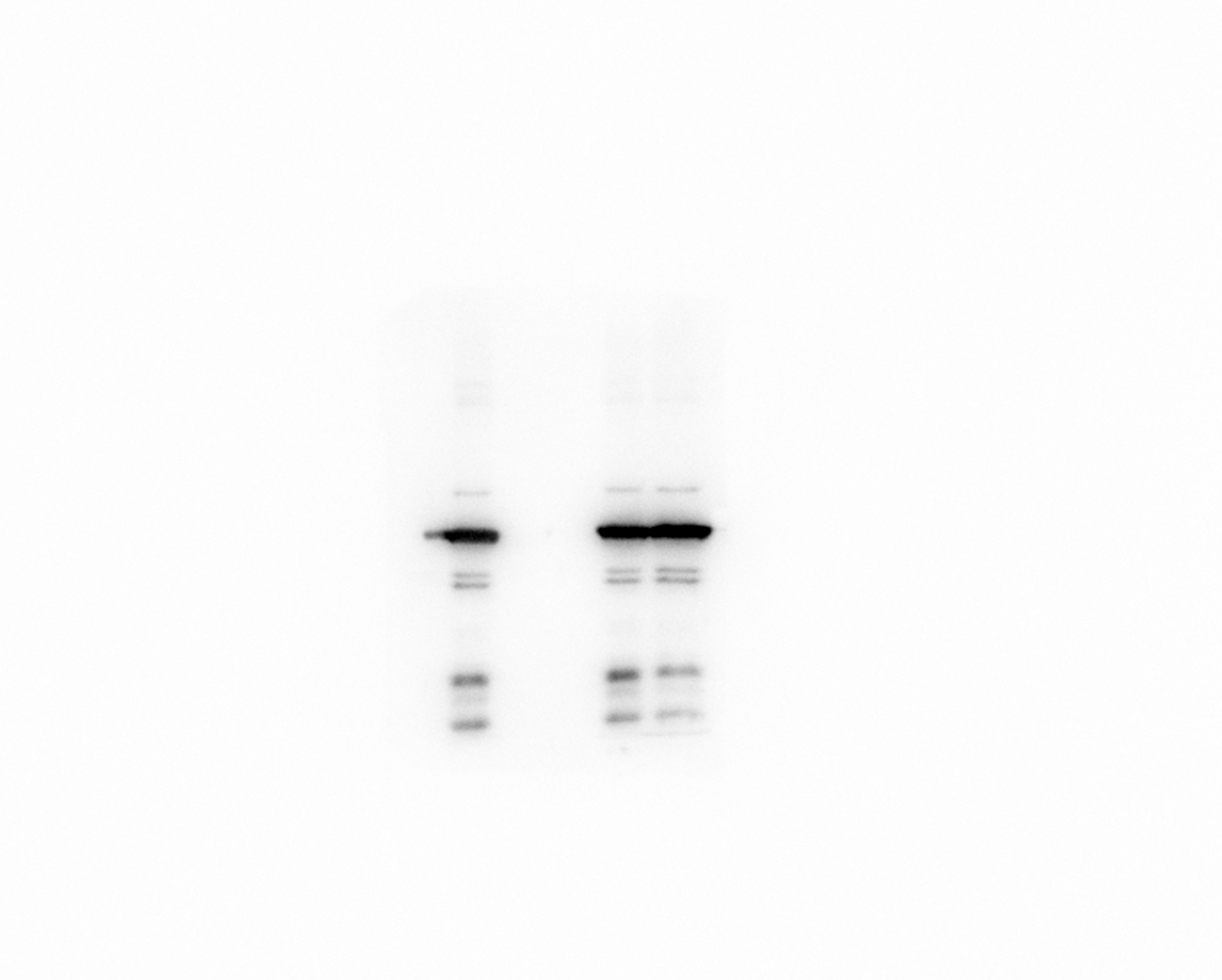


Figure 5D-FLAG Figure 5D-HA Figure 5E-FLAG PARylation Figure 5E-FLAG


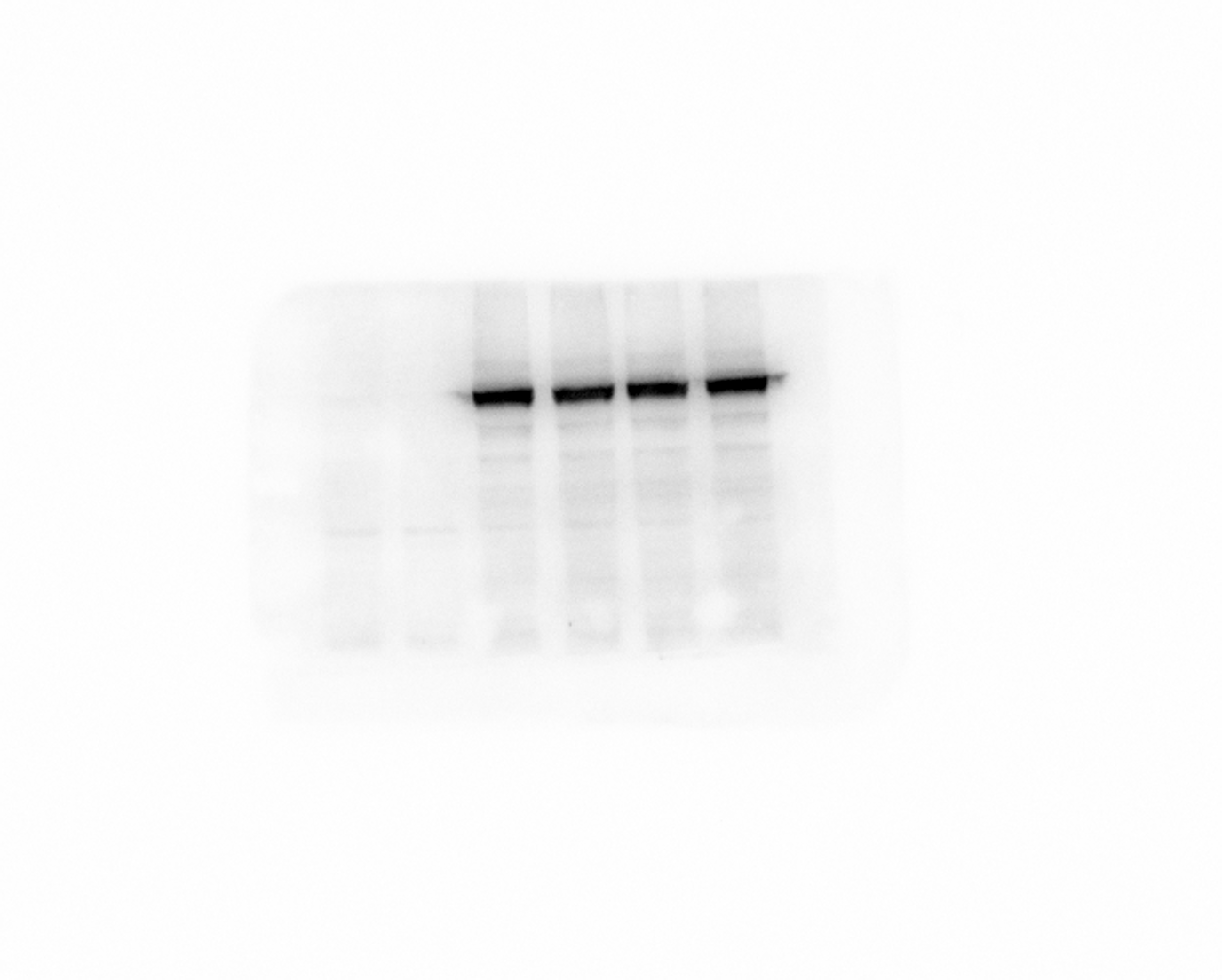

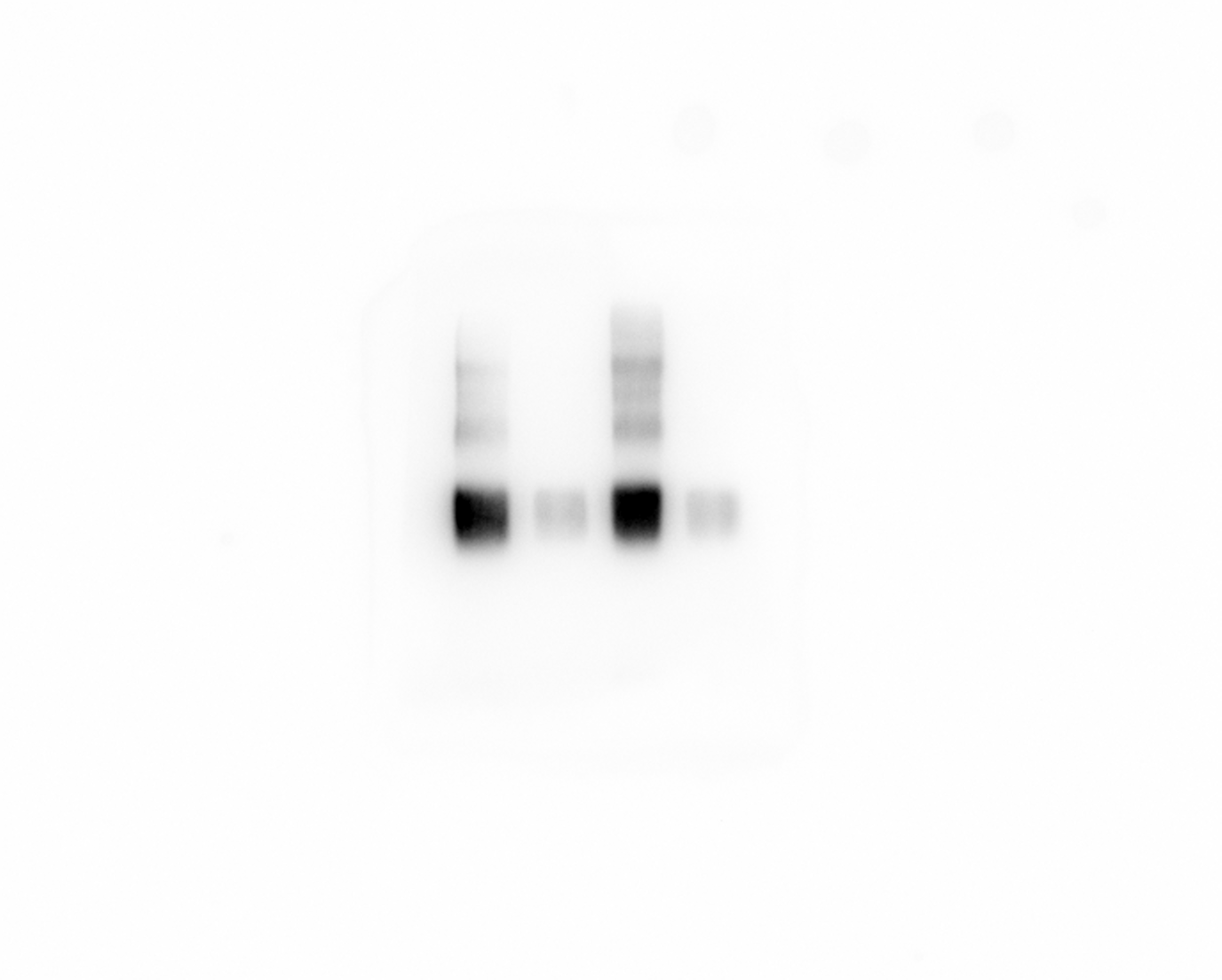

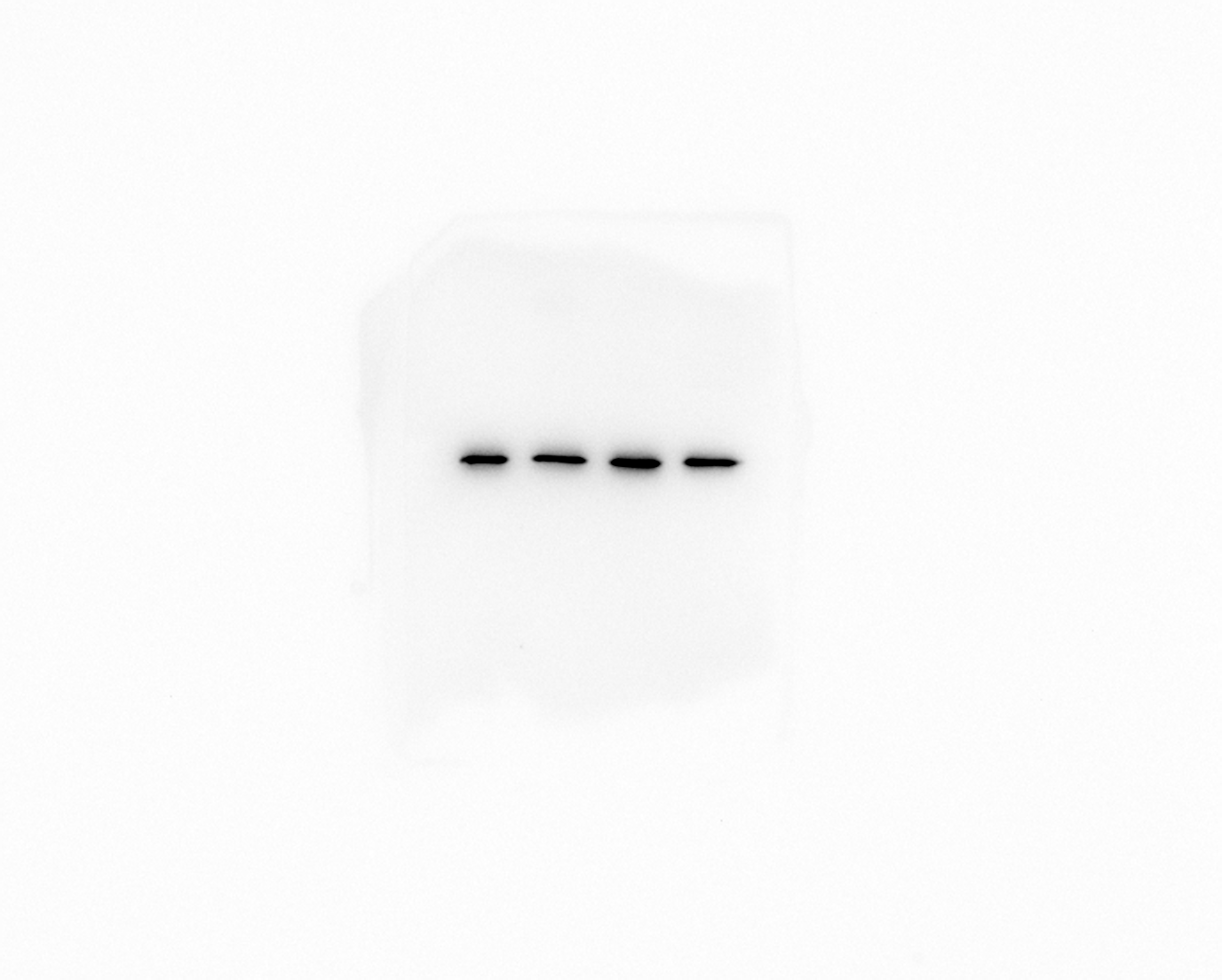

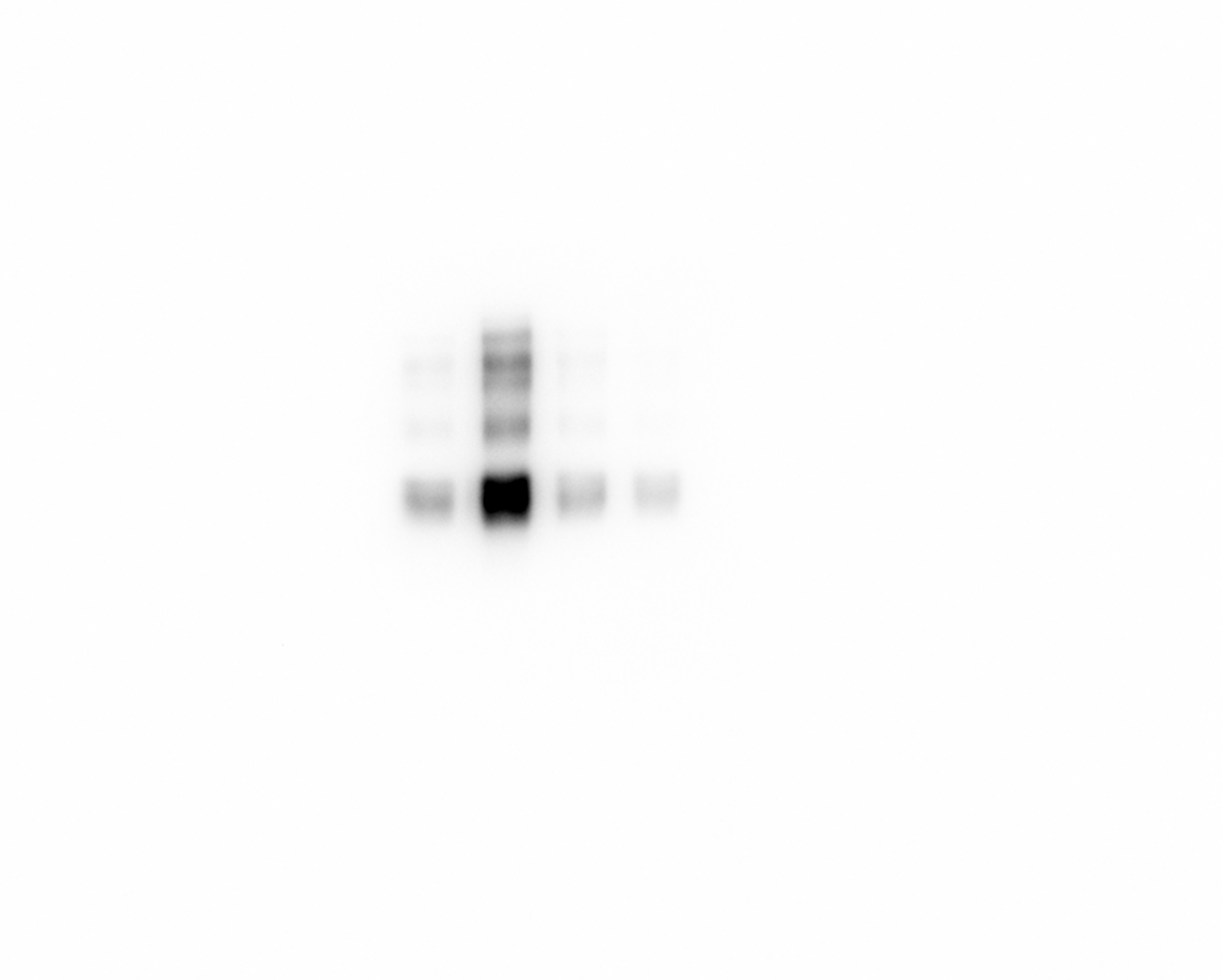


Figure 5E-HA Figure 5F-YY1 PARylation Figure 5F-YY1 Figure 5I-FLAG PARylation


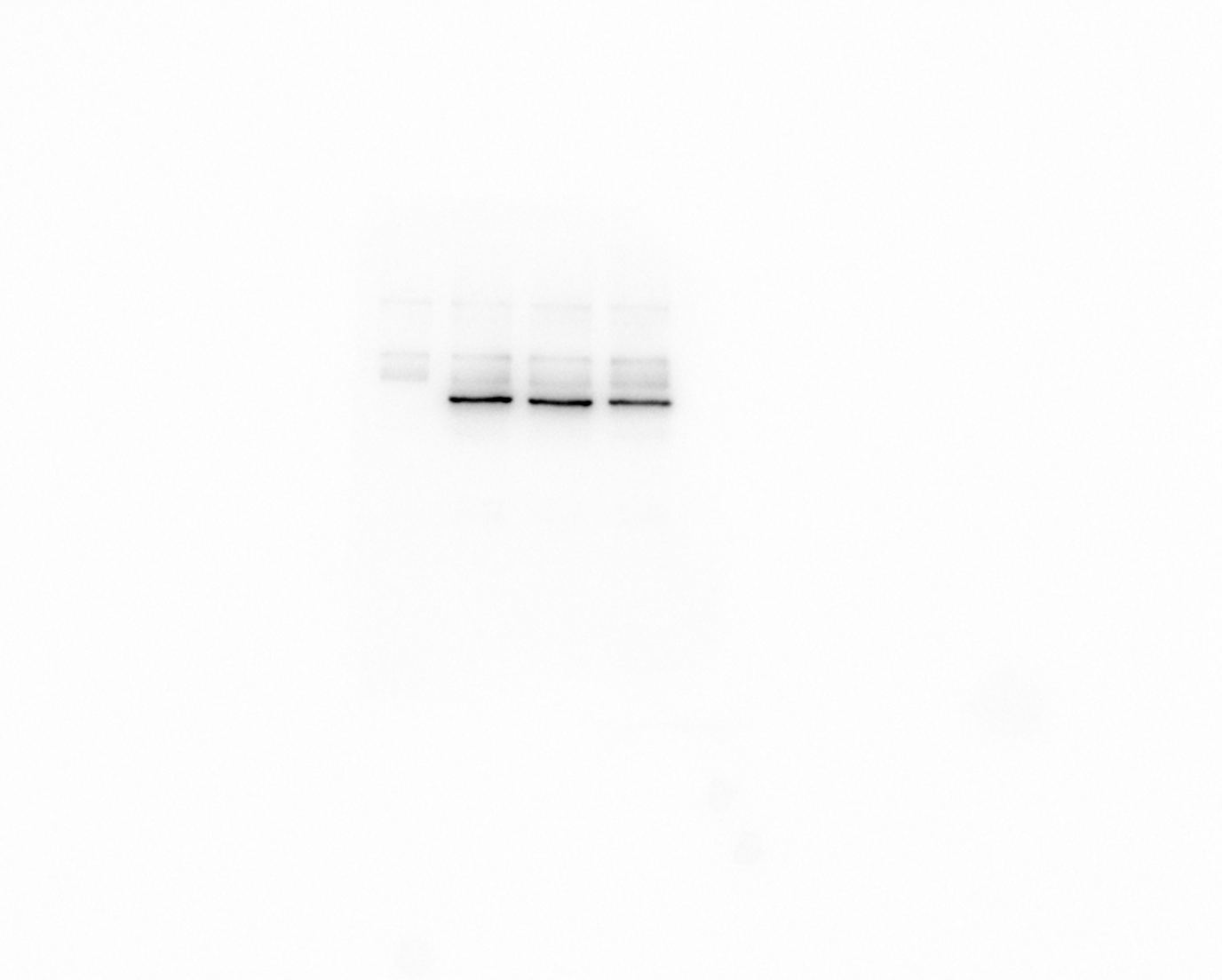

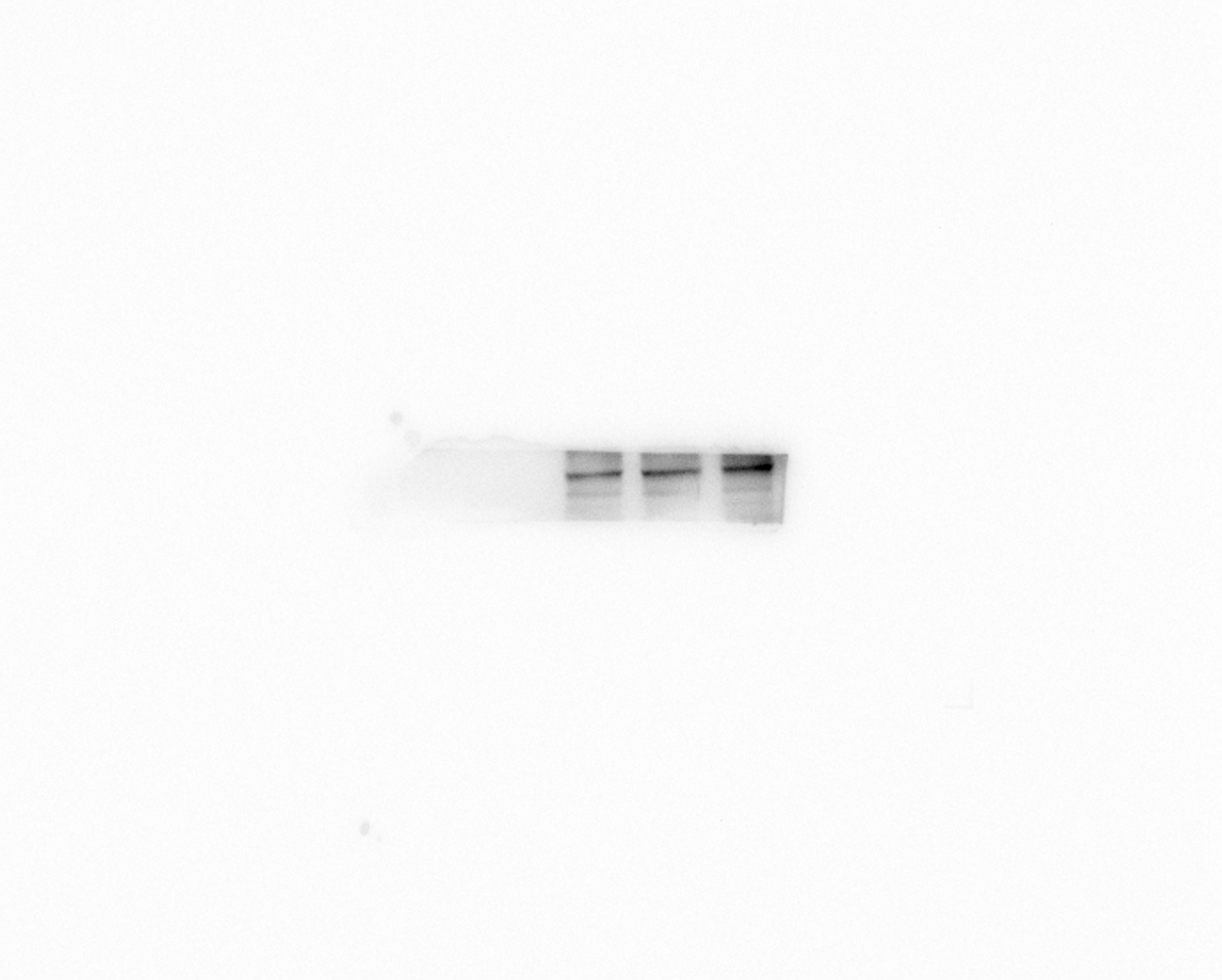


Figure 5I-FLAG Figure 5I-HA


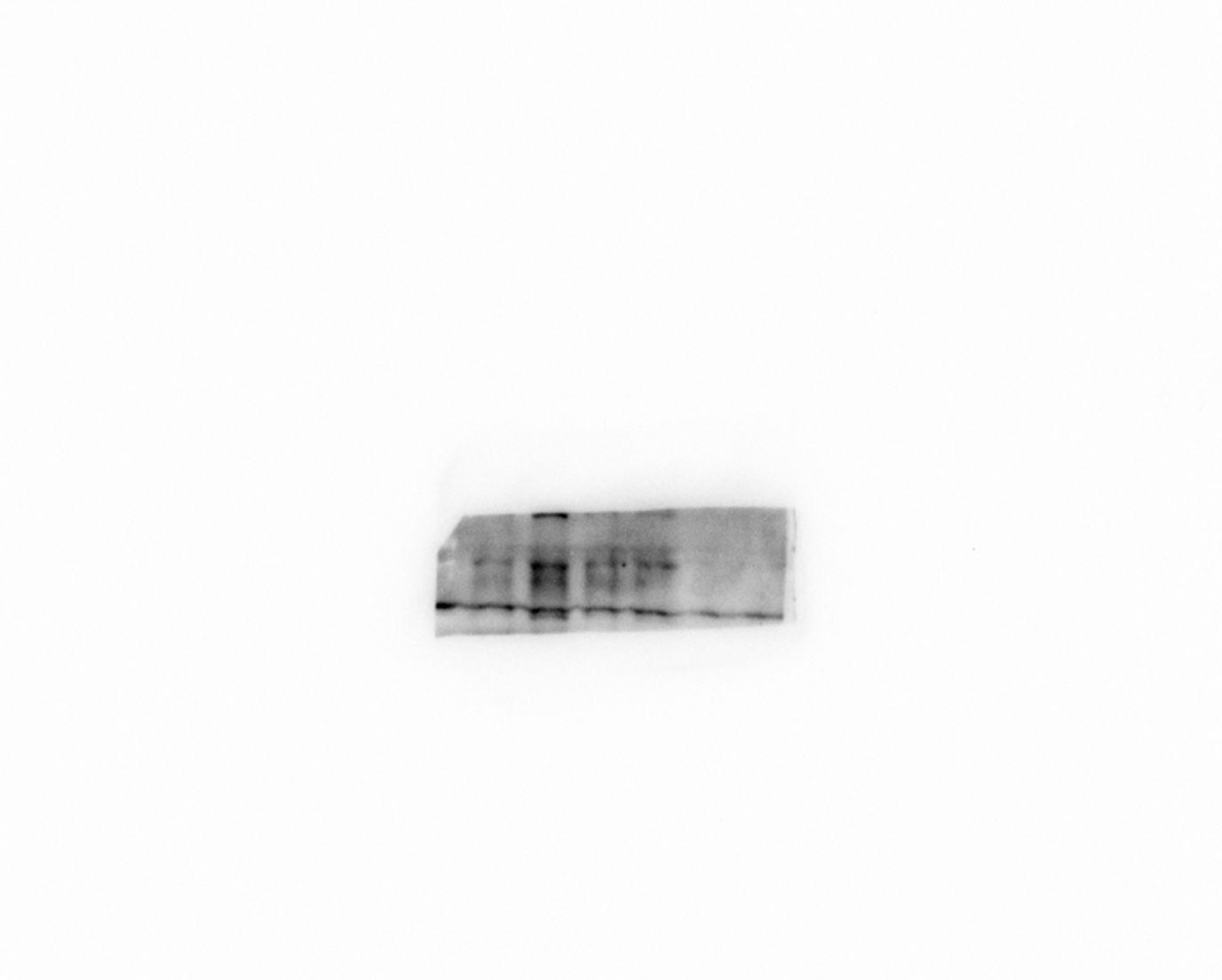

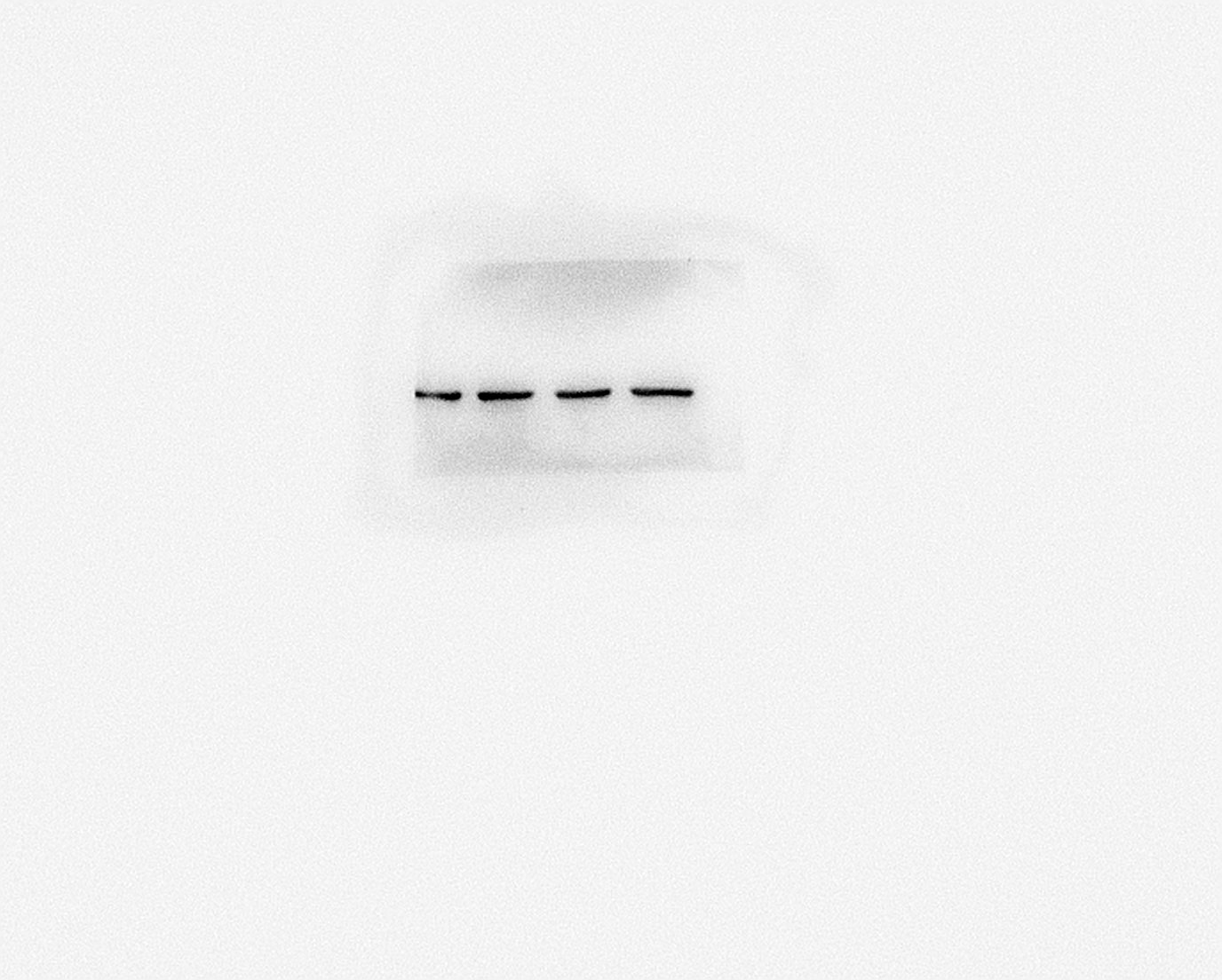

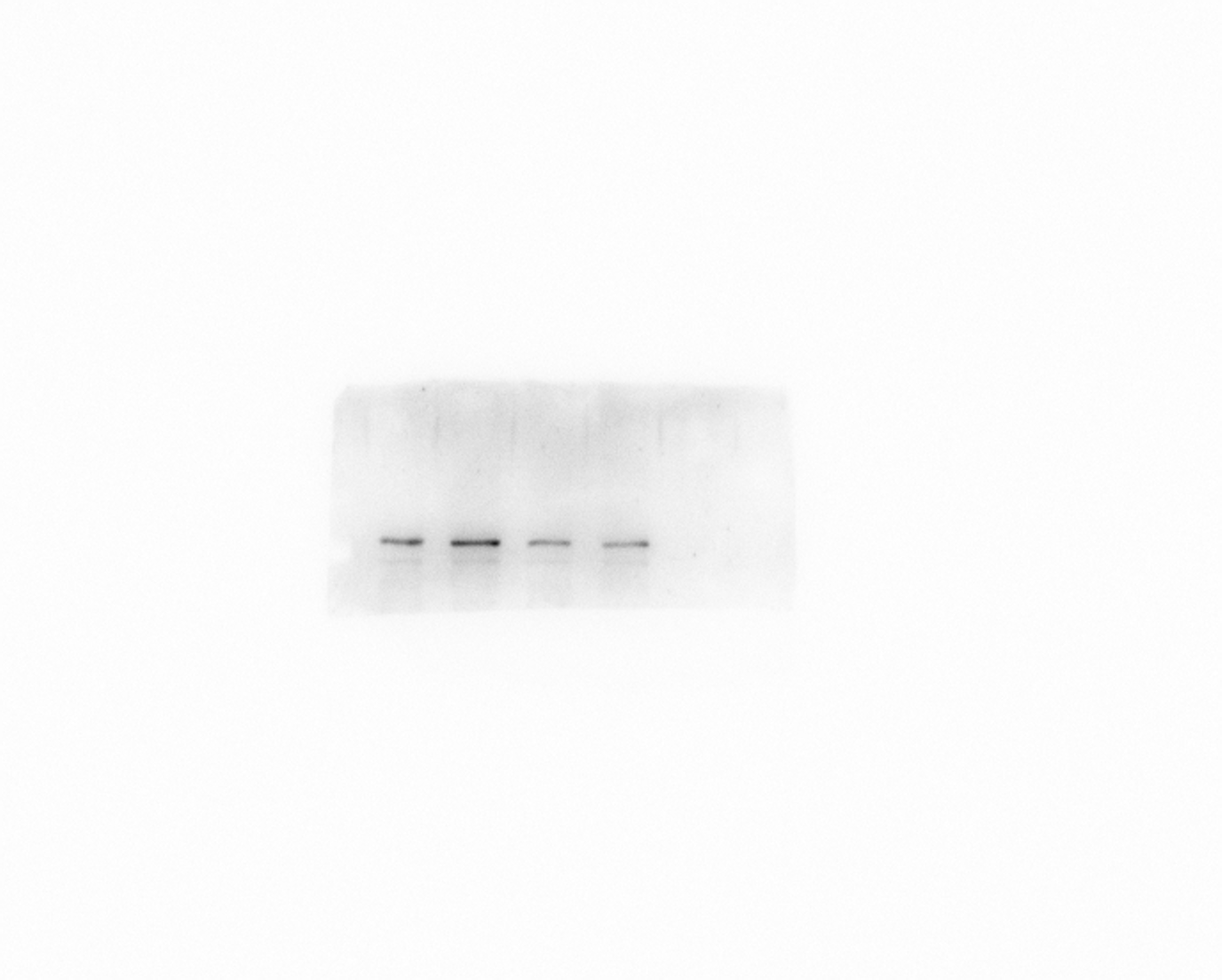

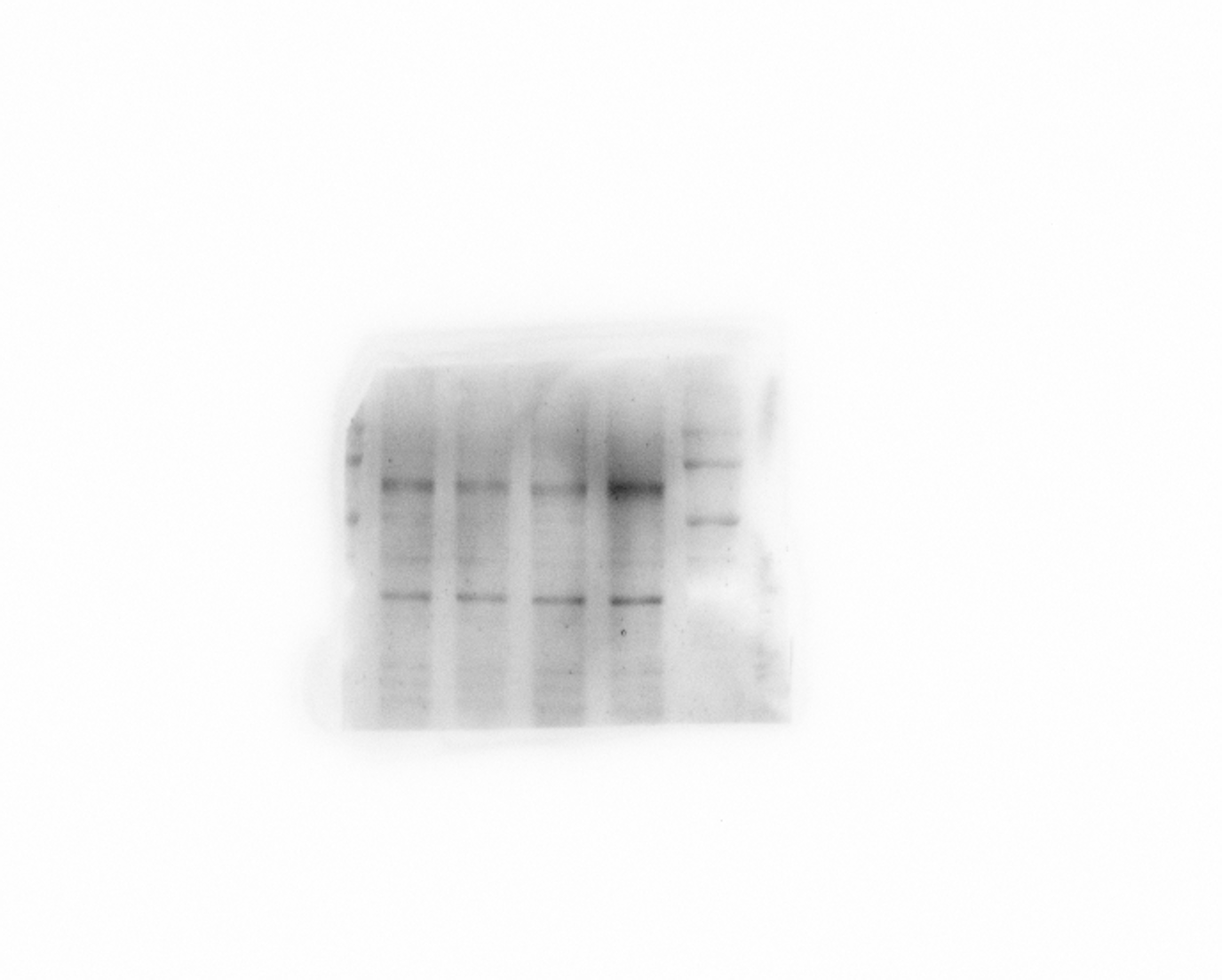

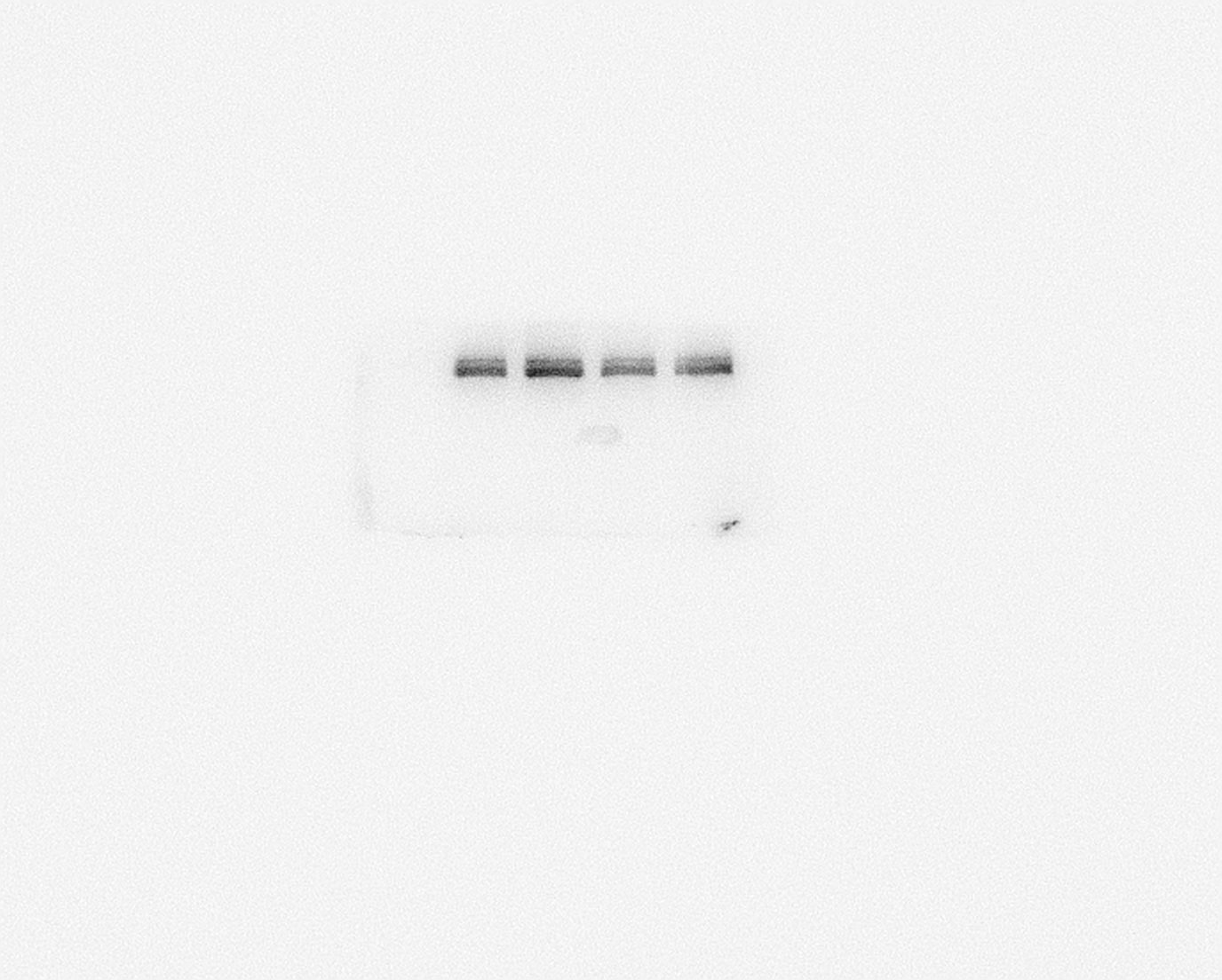


Figure 6D-ATF4 Figure 6D-GAPDH Figure 6D-GRP78 Figure 6D-IRE1α Figure 6D-PARP1


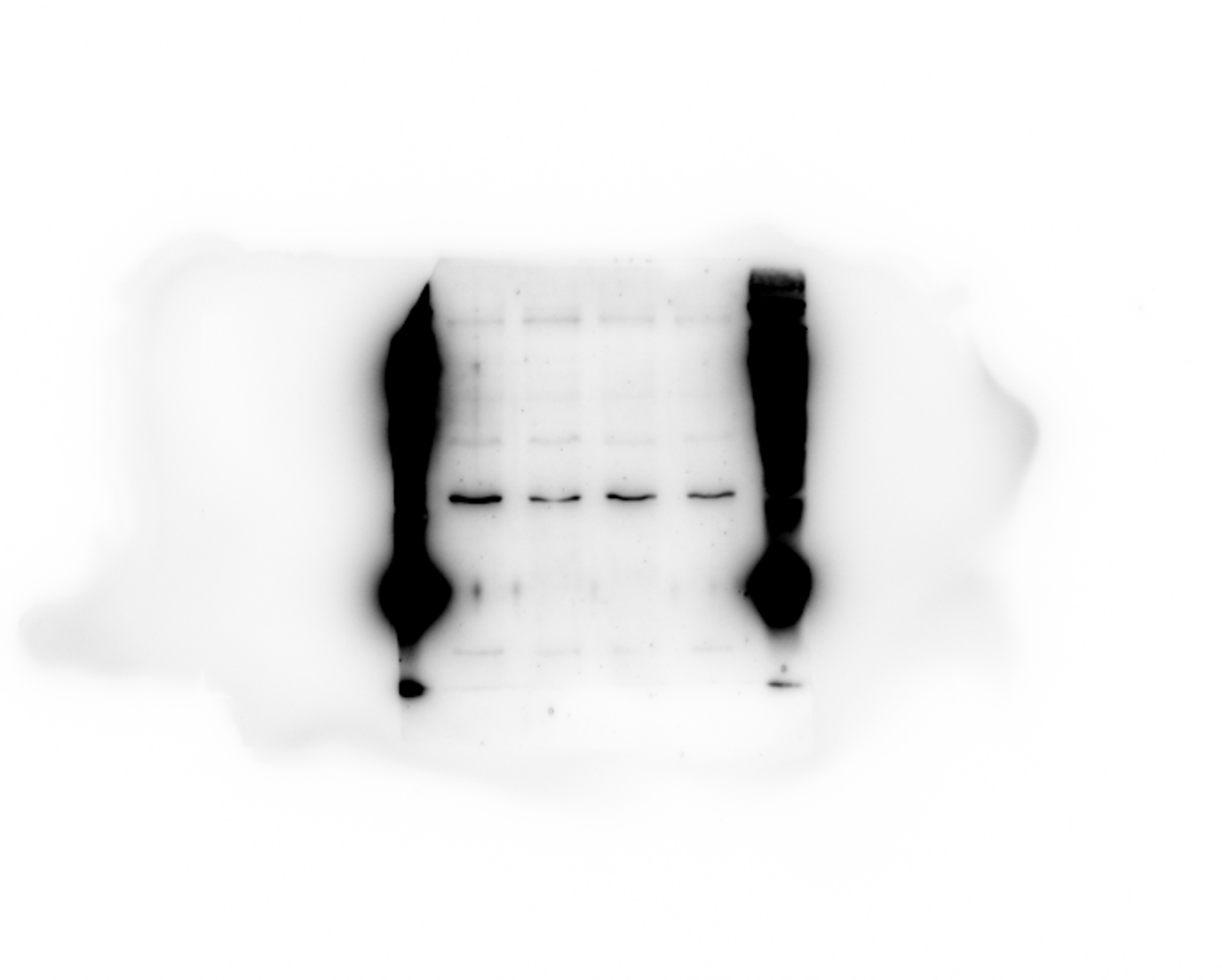

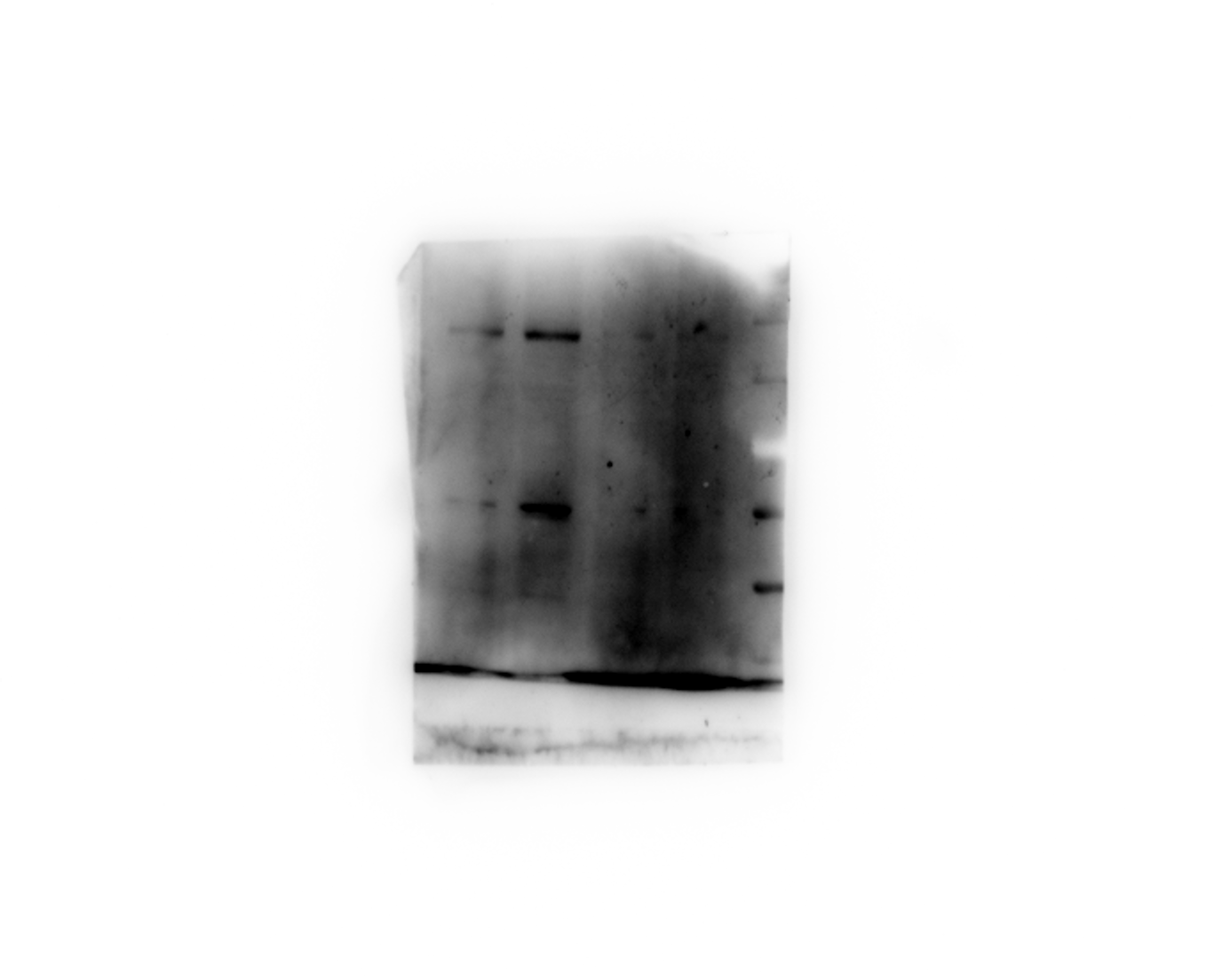

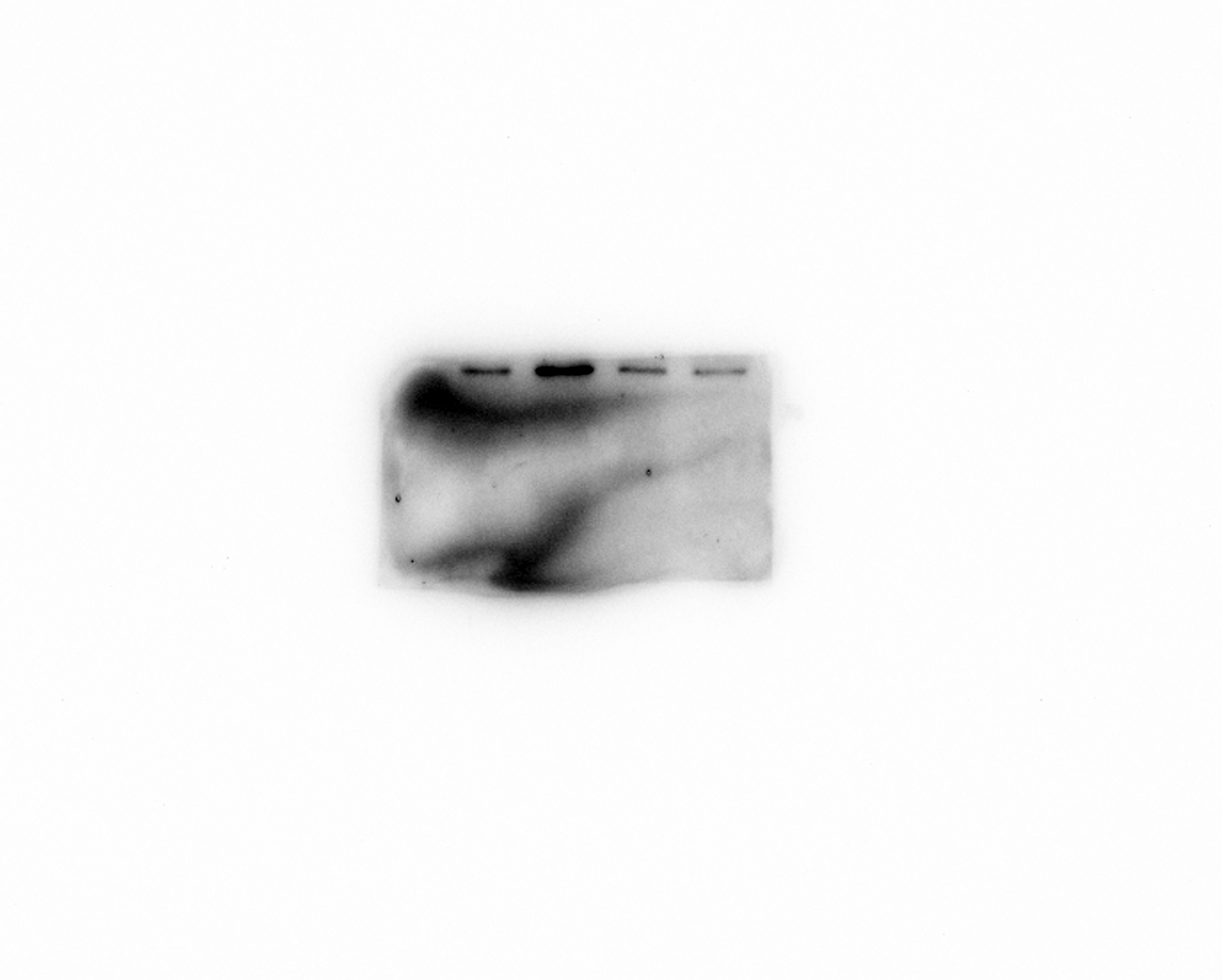

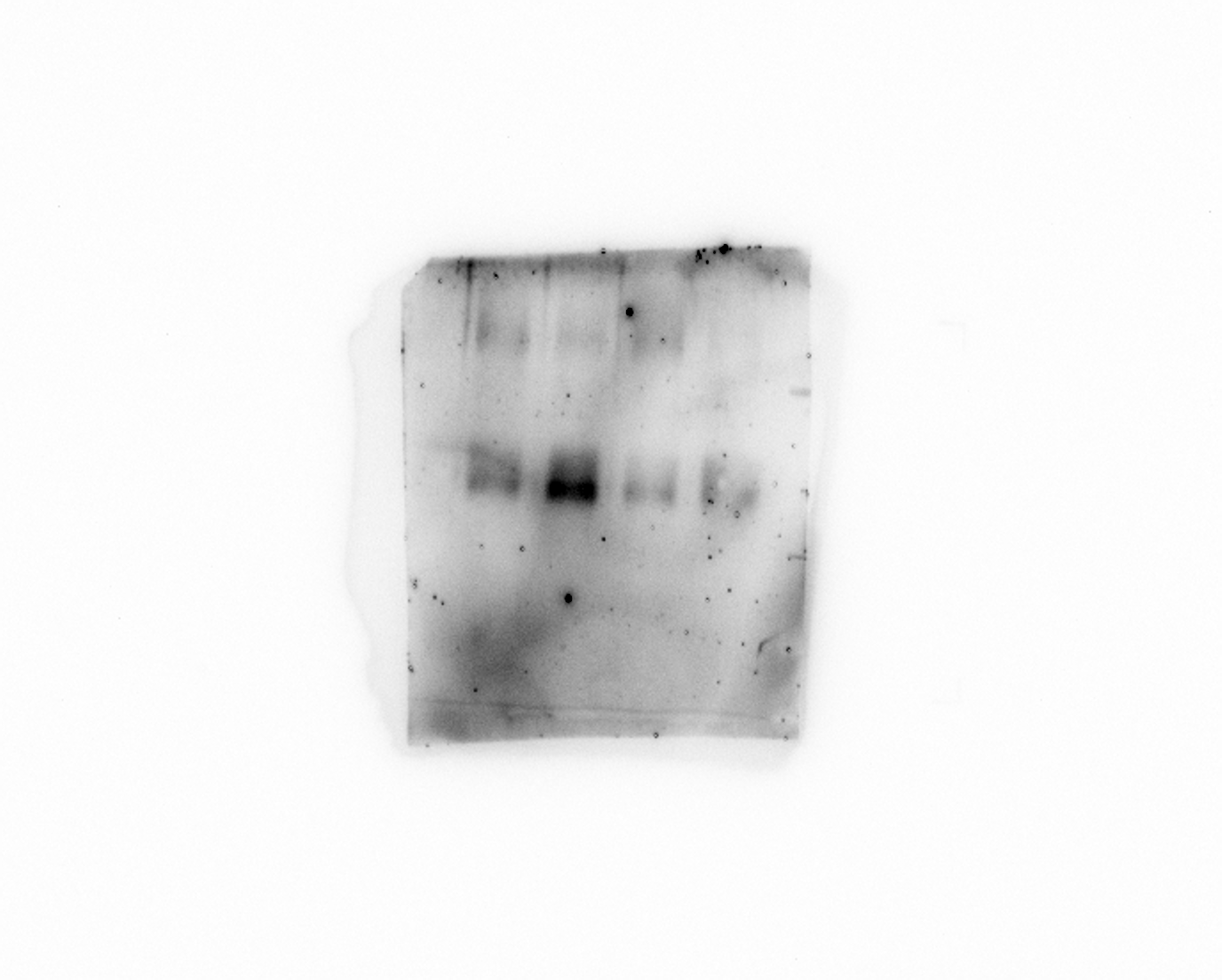


Figure 6D-PERK Figure 6D-pIRE1α Figure 6D-pPERK Figure 6E-YY1 PARylation


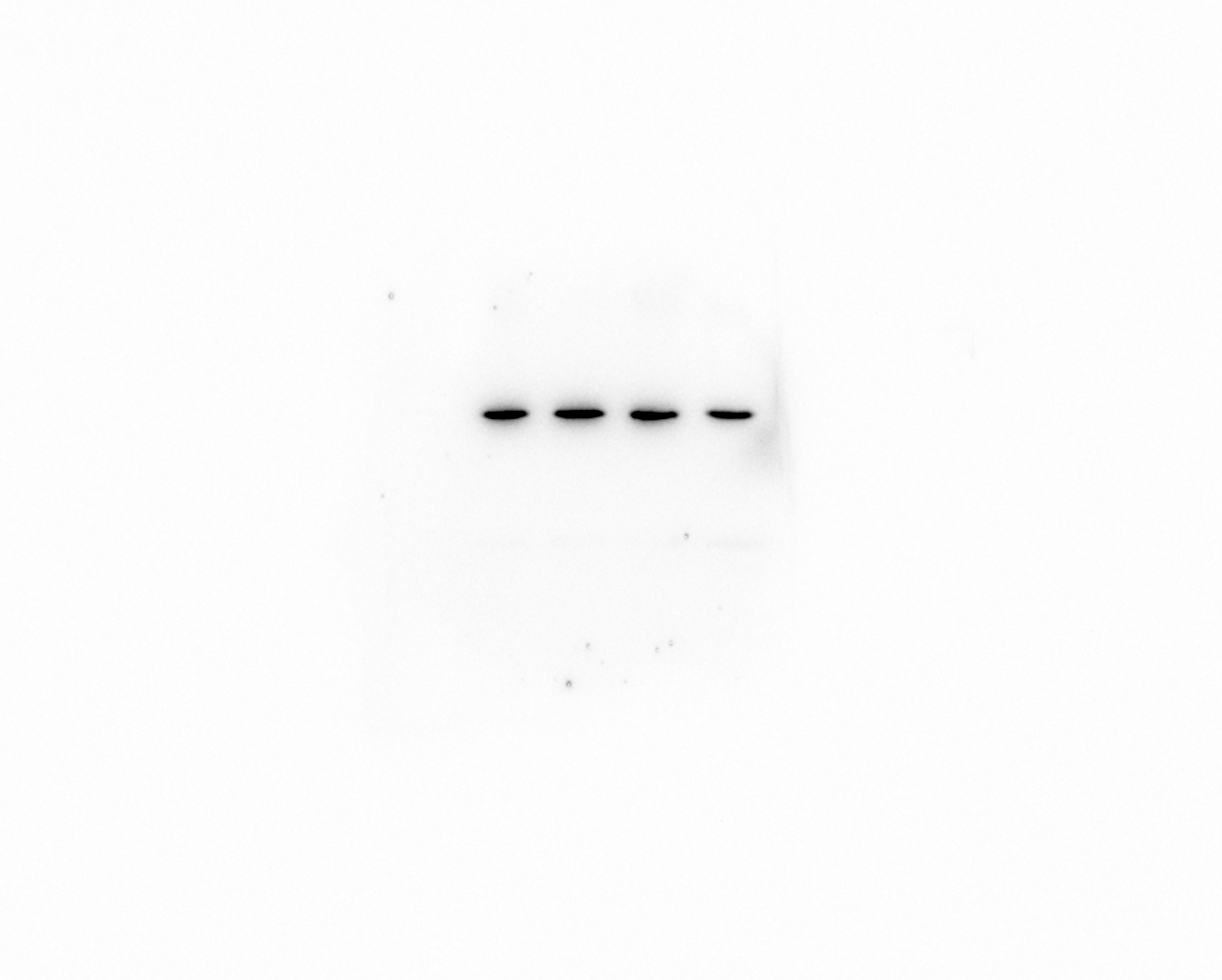

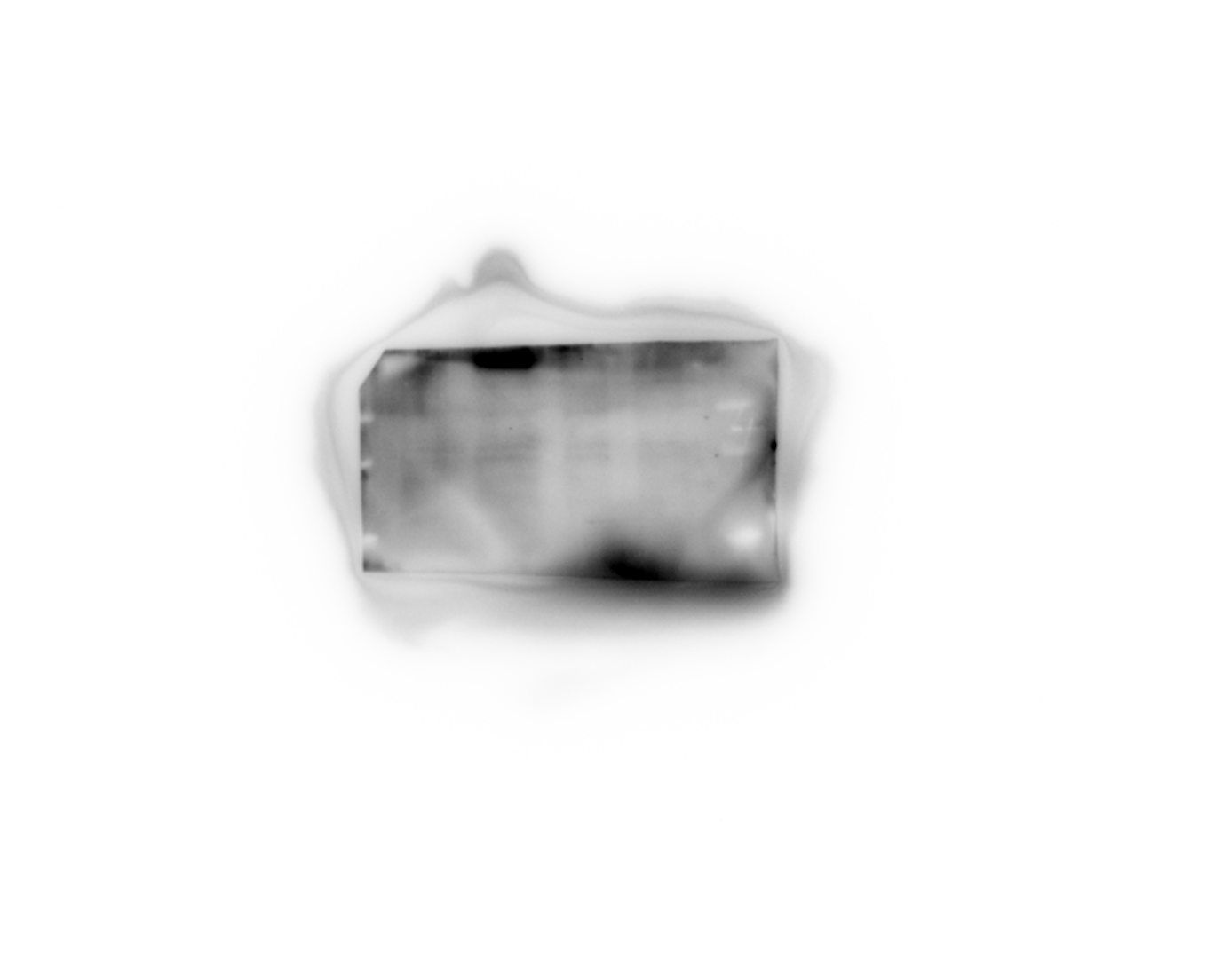

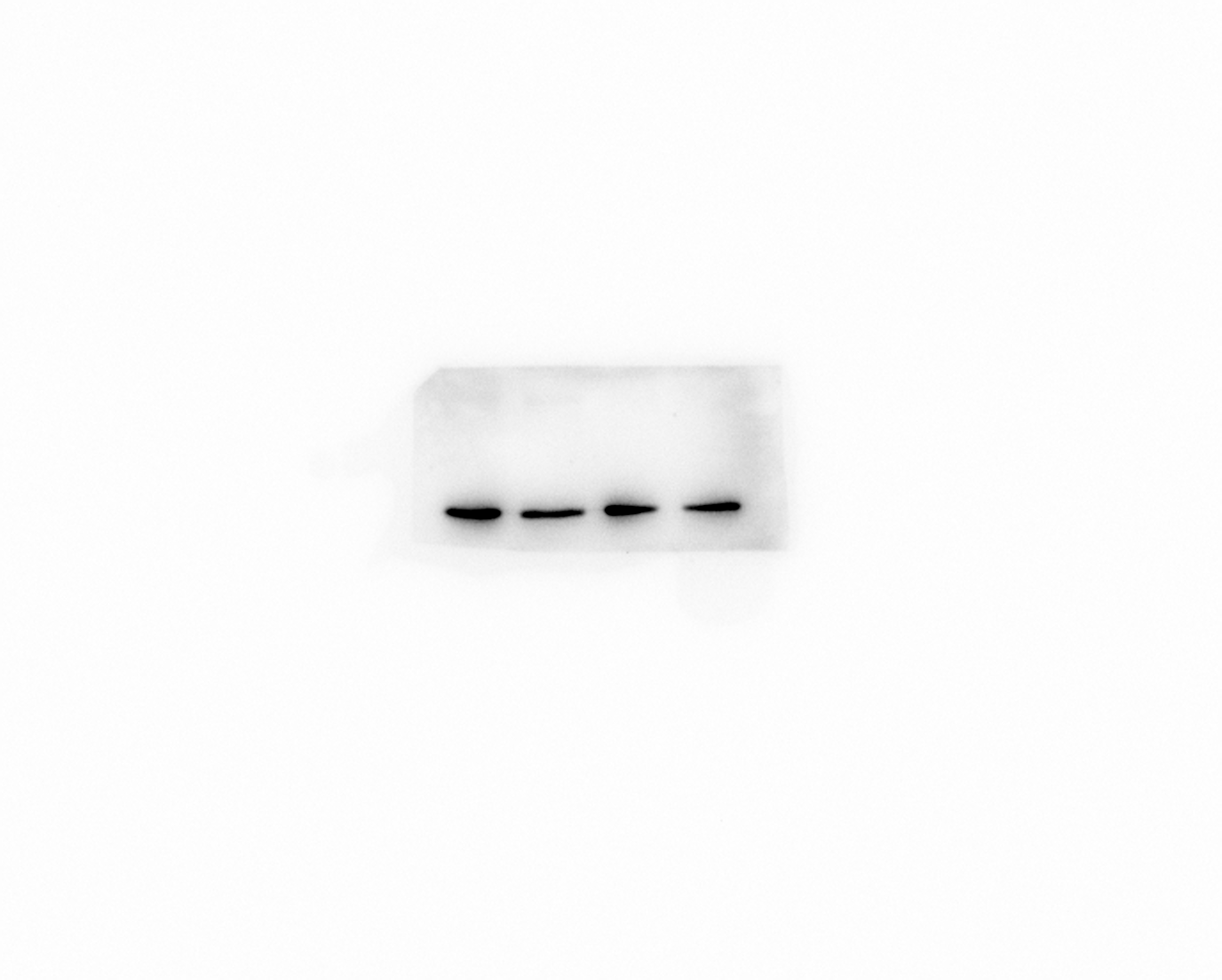

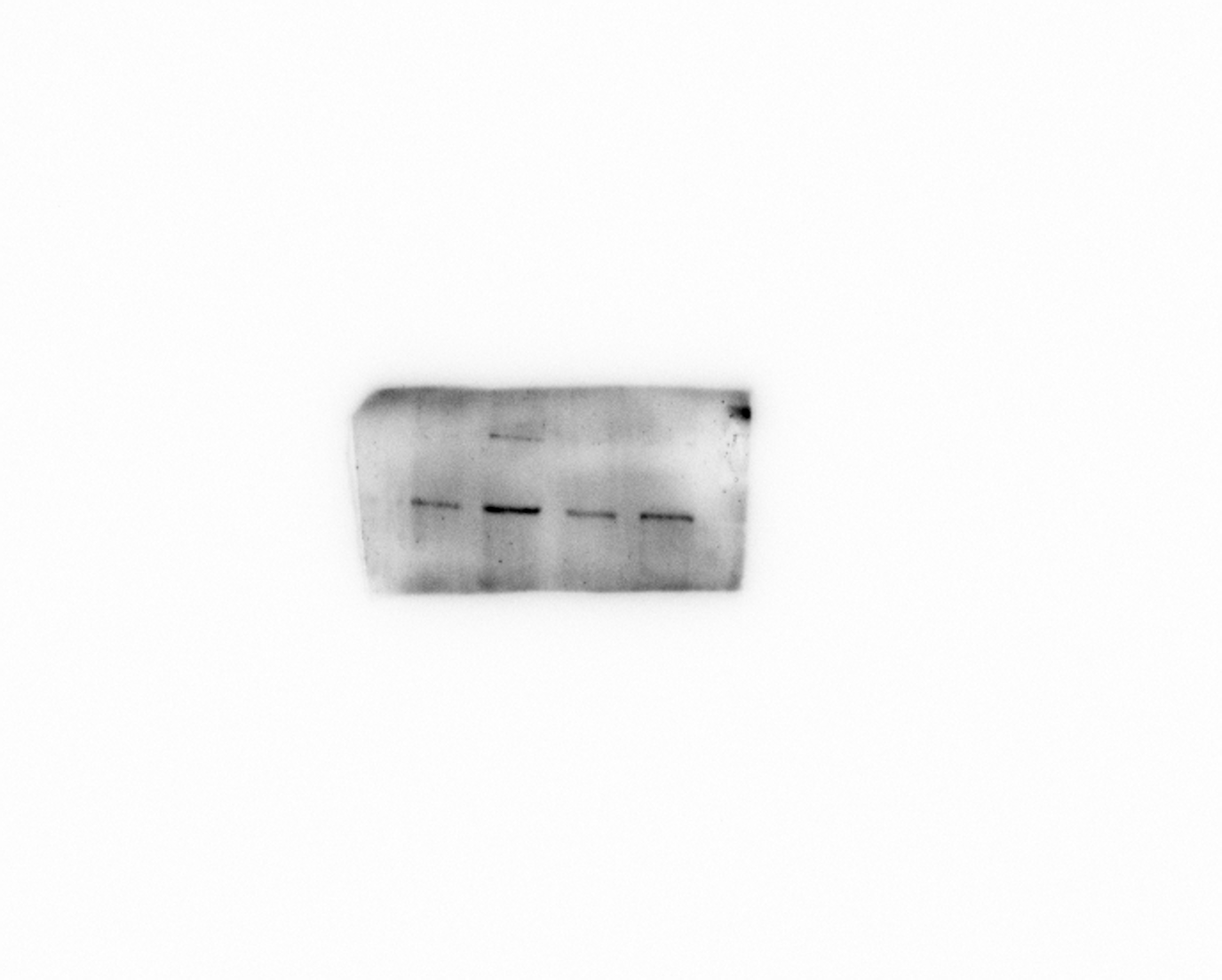

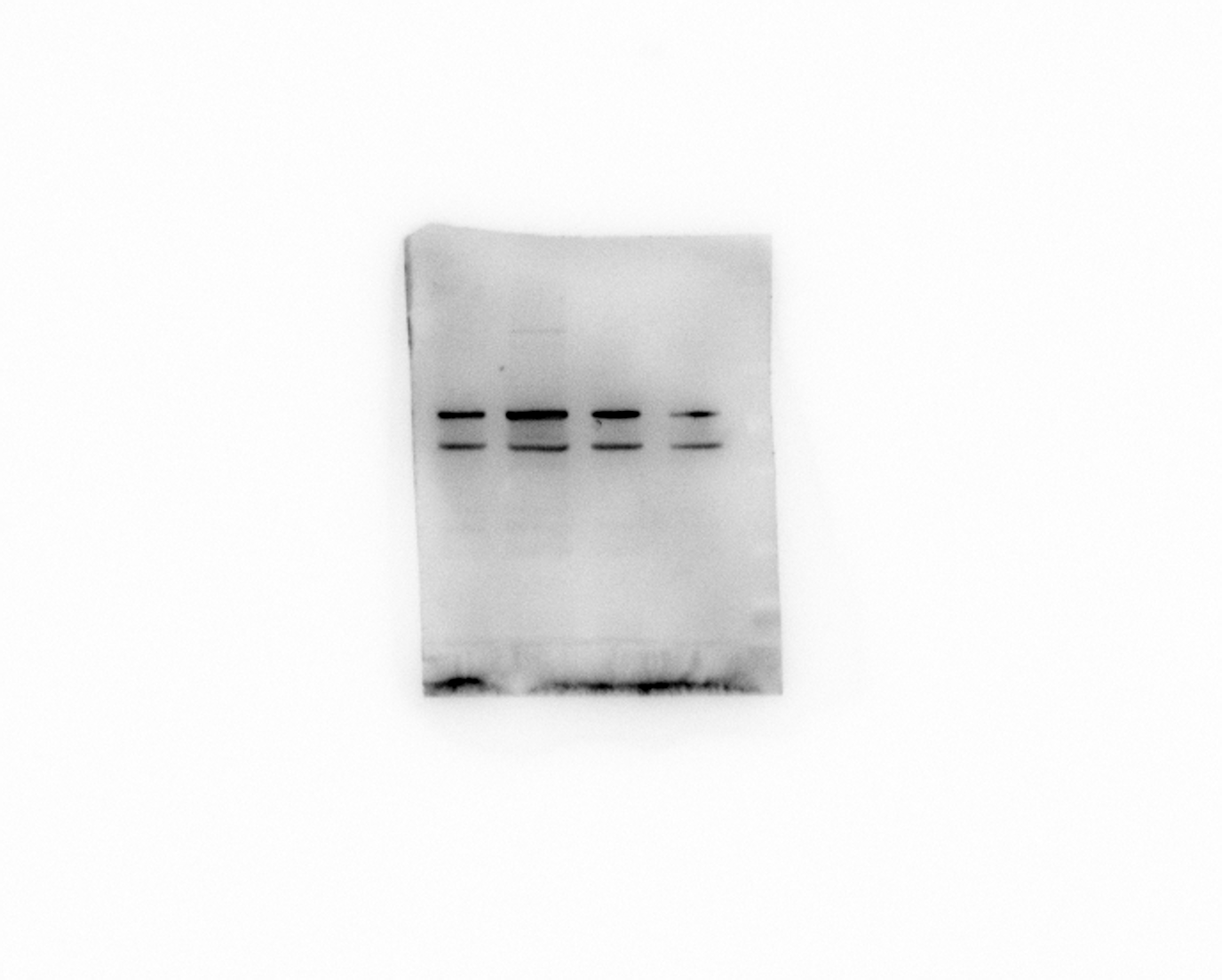


Figure 6E-YY1 Figure 6F-ATF4 Figure 6F-GAPDH Figure 6F-GRP78 Figure 6F-IRE1α


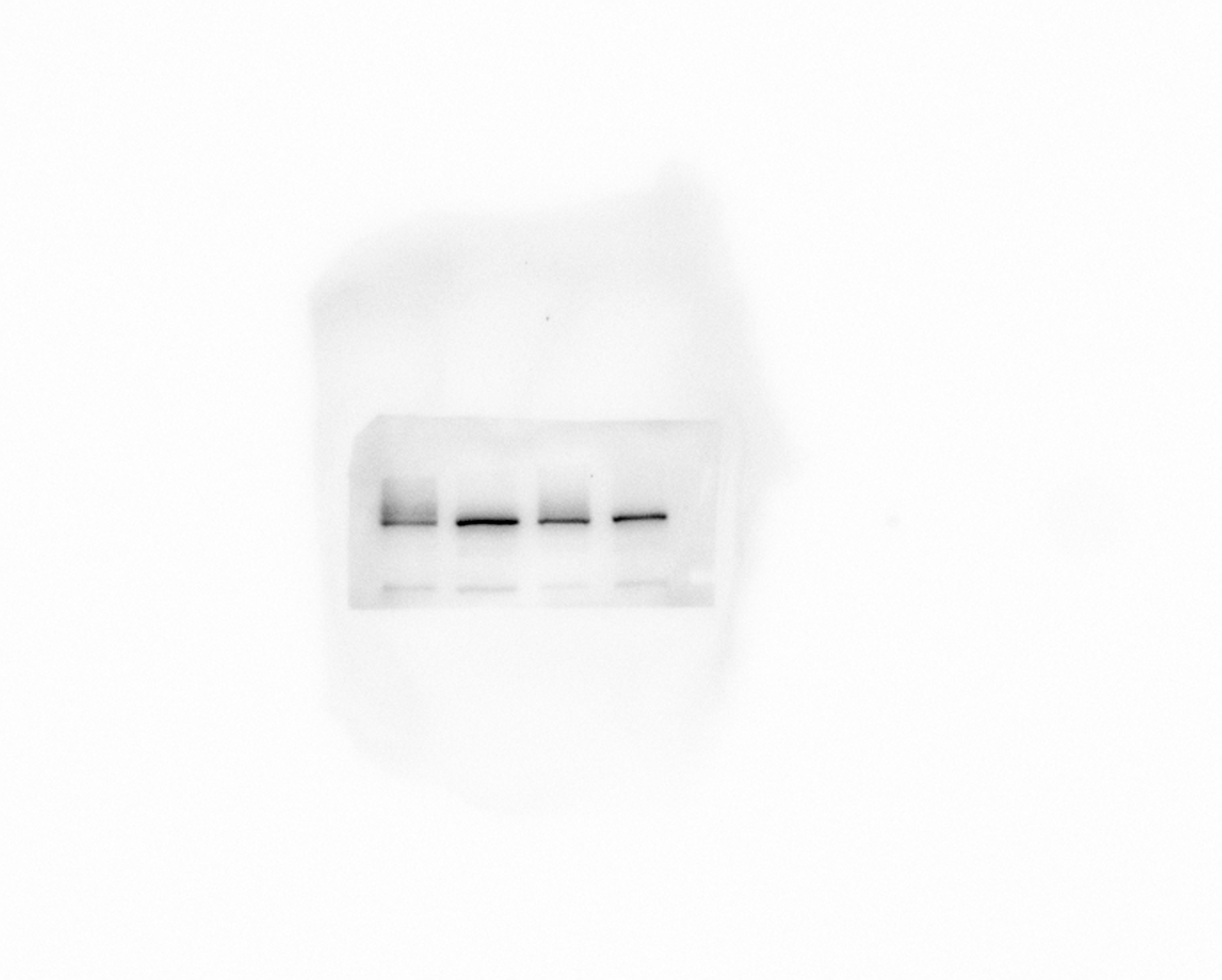

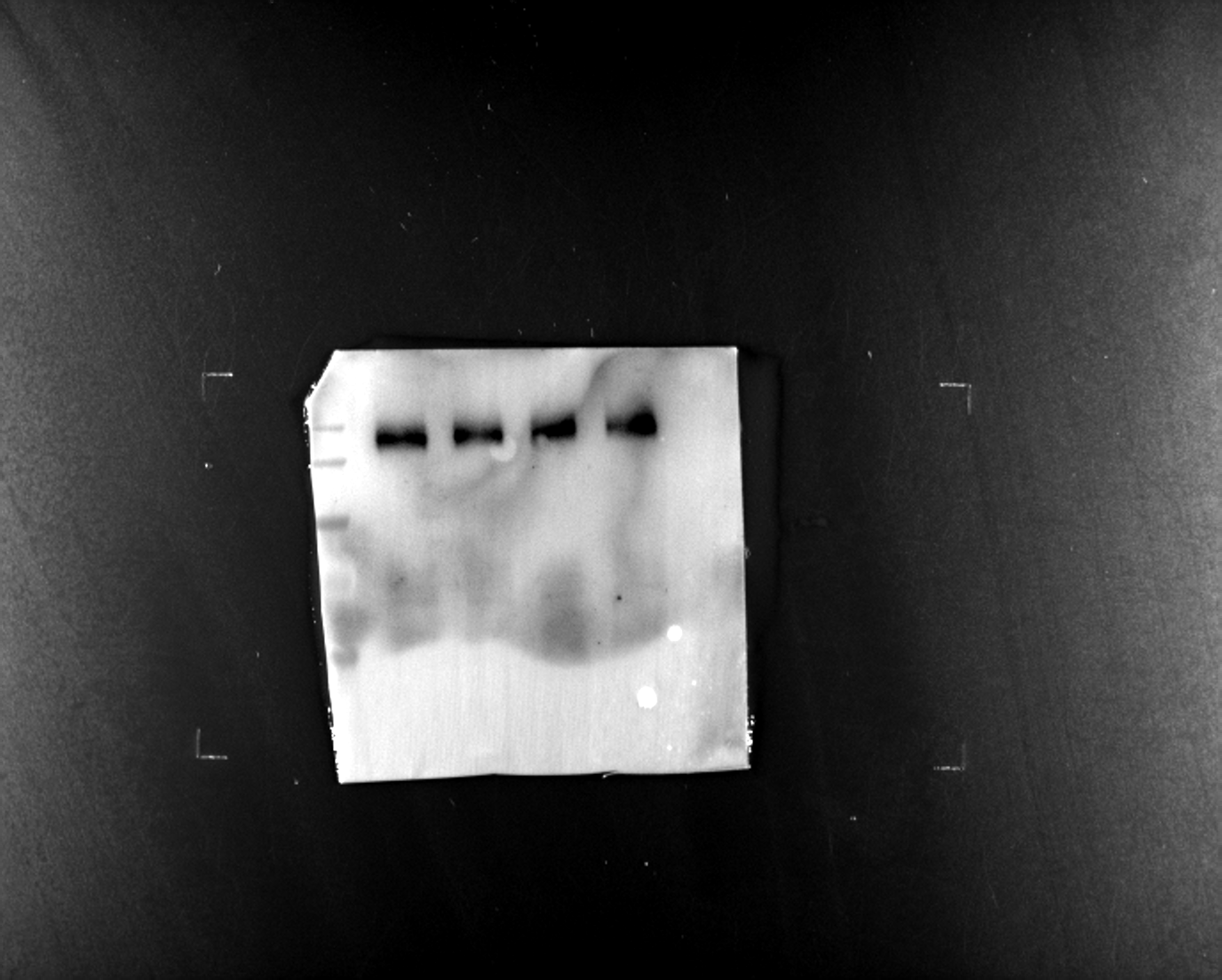

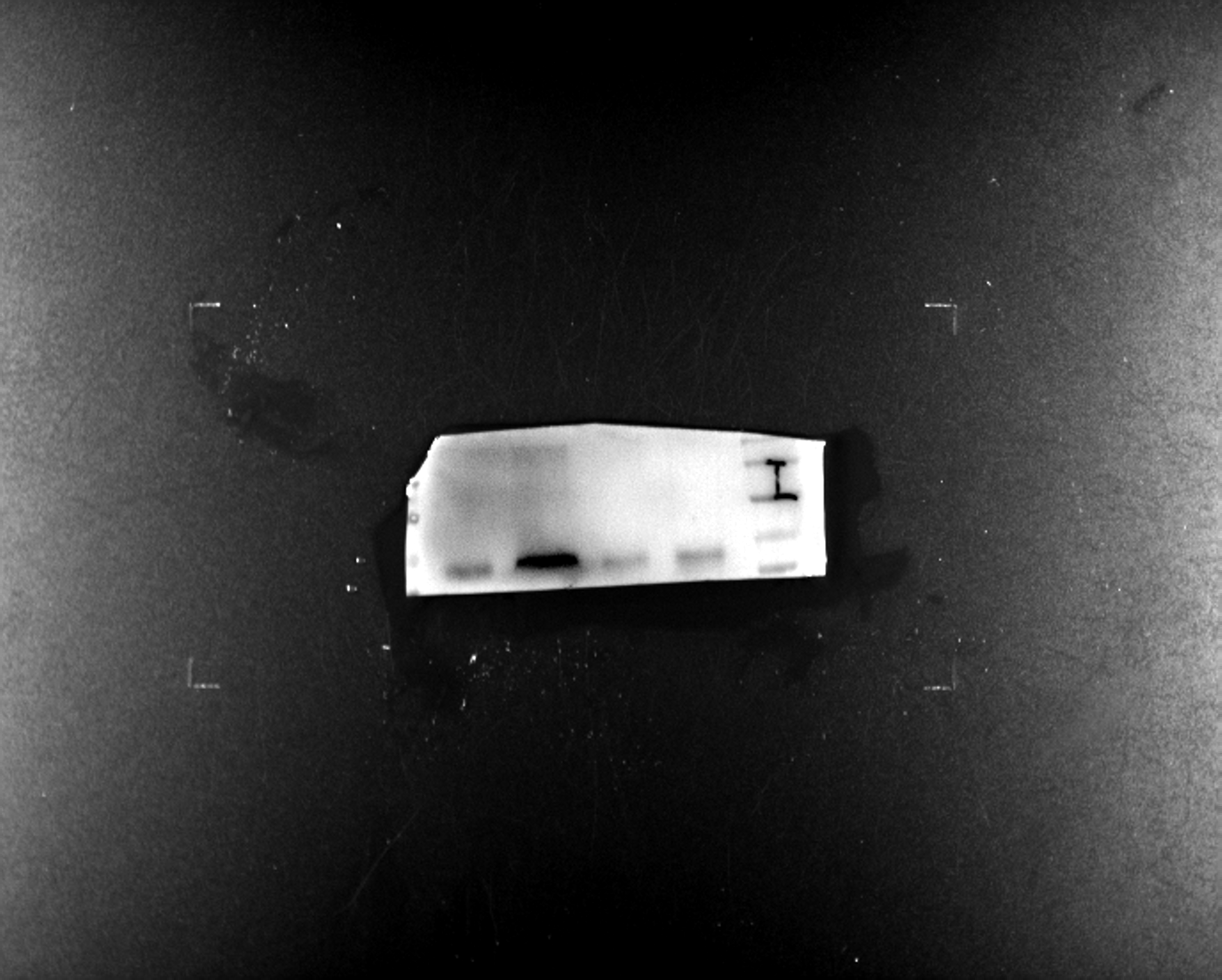

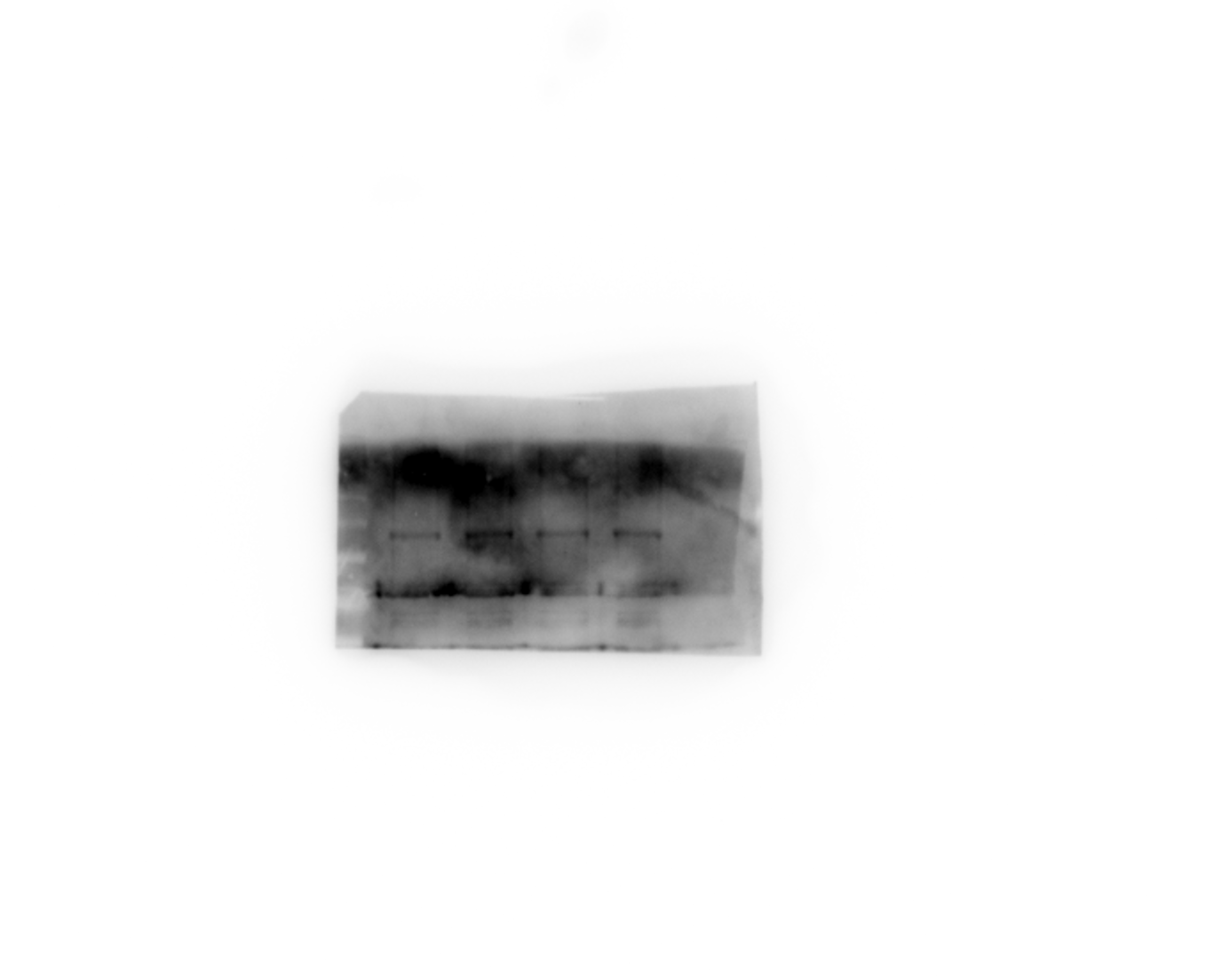


Figure 6F-PARP1 Figure 6F-PERK Figure 6F-pIRE1α Figure 6F-pPERK
